# Enhanced Emitting Dipole Orientation Based on Asymmetric Iridium(III) Complexes for Efficient Saturated‐Blue Phosphorescent OLEDs

**DOI:** 10.1002/advs.202402349

**Published:** 2024-08-13

**Authors:** Kefei Shi, Chengcheng Wu, He Zhang, Kai‐Ning Tong, Wei He, Wansi Li, Zhaoyun Jin, Sinyeong Jung, Siqi Li, Xin Wang, Shaolong Gong, Yuewei Zhang, Dongdong Zhang, Feiyu Kang, Yun Chi, Chuluo Yang, Guodan Wei

**Affiliations:** ^1^ Tsinghua‐Berkeley Shenzhen Institute (TBSI) Tsinghua University Shenzhen 518055 China; ^2^ Institute of Materials Research Tsinghua Shenzhen International Graduate School Tsinghua University Shenzhen 518055 China; ^3^ Hubei Key Lab on Organic and Polymeric Optoelectronic Materials Department of Chemistry Wuhan University Wuhan 430072 China; ^4^ Laboratory of Flexible Electronics Technology Tsinghua University Beijing China; ^5^ Key Laboratory of Organic Optoelectronics and Molecular Engineering of Ministry of Education, Department of Chemistry Tsinghua University Beijing China; ^6^ Department of Materials Science and Engineering Department of Chemistry and Center of Super‐Diamond and Advanced Films (COSDAF) City University of Hong Kong Hong Kong SAR 999077 China; ^7^ Shenzhen Key Laboratory of New Information Display and Storage Materials College of Materials Science and Engineering Shenzhen University Shenzhen 518060 China

**Keywords:** iridium, molecular orientation, OLED, outcoupling, phosphorescence

## Abstract

Three novel asymmetric Ir(III) complexes have been rationally designed to optimize their emitting dipole orientations (EDO) and enhance light outcoupling in blue phosphorescent organic light‐emitting diodes (OLEDs), thereby boosting their external quantum efficiency (*EQE*). Bulky electron‐donating groups (EDGs), namely: carbazole (Cz), di‐tert‐butyl carbazole (tBuCz), and phenoxazine (Pxz) are incorporated into the tridentate dicarbene pincer chelate to induce high degree of packing anisotropy, simultaneously enhancing their photophysical properties. Angle‐dependent photoluminescence (ADPL) measurements indicate increased horizontal transition dipole ratios of 0.89 and 0.90 for the Ir(III) complexes **Cz‐dfppy‐CN** and **tBuCz‐dfppy‐CN**, respectively. Analysis of the single crystal structure and density functional theory (DFT) calculation results revealed an inherent correlation between molecular aspect ratio and EDO. Utilizing the newly obtained emitters, the blue OLED devices demonstrated exceptional performance, achieving a maximum *EQE* of 30.7% at a Commission International de l'Eclairage (CIE) coordinate of (0.140, 0.148). Optical transfer matrix‐based simulations confirmed a maximum outcoupling efficiency of 35% due to improved EDO. Finally, the tandem OLED and hyper‐OLED devices exhibited a maximum *EQE* of 44.2% and 31.6%, respectively, together with good device stability. This rational molecular design provides straightforward guidelines to reach highly efficient and stable saturated blue emission.

## Graphical abstract

Bulky and electron‐donating substituents on the asymmetric Ir(III) complexes have greatly improved the emitting dipole orientation (EDO) and their blue OLED devices have CIE(x,y) color coordinates of (0.140, 0.148) and high external quantum efficiency of 30.7%.

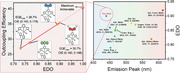

## 1 Introduction

Organic light‐emitting diodes (OLEDs) have been widely utilized in the display technology of consumer electronics due to their practical advantages, such as high contrast ratio, flexibility, and compact device structures.^[^ ^1^, ^2^ ^]^ In order to achieve an energy‐efficient display that is suitable for portable electronic applications, significant efforts have been made to improve their external quantum efficiency (*EQE*), which reflects the combined contribution of several processes, as described by the following equation:^[^ ^3^ ^]^

(1) $$\;\eta_{\mathrm{EQE}}\equiv \eta_{\mathrm{int}}\times \;\eta_{\mathrm{out}}=\;\chi \times \eta_{r}\times q_{\mathrm{eff}}\times \eta_{\mathrm{out}}$$

Here, *χ* is the charge balance factor, *η*_r_ is the exciton utilization ratio, *q*_eff_ is the effective photoluminescence quantum yield (PLQY) of the emitter, and *η*_out_ represents the outcoupling efficiency. Generally, phosphorescent emitters, such as Ir(III),^[^ ^4^, ^5^, ^6^, ^7^, ^8^ ^]^ Pd(II),^[^ ^9^, ^10^ ^]^ Pt(II),^[^ ^11^, ^12^, ^13^ ^]^ and Au(III)^[^ ^14^, ^15^, ^16^ ^]^ complexes, facilitate efficient triplet harvesting through spin‐orbit coupling (SOC) that induced by the heavy metal atom.^[^ ^17^, ^18^, ^19^ ^]^ Moreover, high PLQY was achieved by the rational design of molecular structures and excited‐state engineering.^[^ ^20^, ^21^ ^]^ Additionally, an unitary charge balance factor *χ* can be guaranteed by the optimization of carrier injection and transportation channels. Together with high PLQY and full utilization of all excitons, the theoretical internal quantum efficiency (*η*_int_) is close to 100%. Unfortunately, triplet‐triplet annihilation (TTA) and triplet‐polaron quenching (TPQ) could afford the additional channels for energy dissipation.^[^ ^22^ ^]^ This issue is particularly severe for blue phosphorescence OLEDs (PhOLEDs), where long‐lifetime high‐energy triplet excitons could inevitably cause notable photo‐degradation and formation of hot excitons, leading to relatively inferior *EQE* and poor device stability.^[^ ^23^ ^]^ Hence, there exists a significant imperative to enhance device performance through fully harvesting of both the singlet and triplet excitons.

Apart from external outcoupling approaches that involve in modifications of device structures, such as corrugated structures,^[^ ^24^, ^25^ ^]^ microlenses,^[^ ^26^, ^27^ ^]^ scattering layers^[^ ^28^, ^29^ ^]^ and substrate engineering,^[^ ^30^, ^31^ ^]^ the horizontal alignment of transition dipole moments (TDMs) has been found to have a significant impact on reducing light dissipation at organic thin film interfaces, thus increasing the ratio of extracted light without additional complicated optical structures.^[^ ^32^ ^]^ Extensive research on Ir(III) complexes has indicated that the emitting dipole orientation (EDO) is influenced by various factors including molecular aspect ratio,^[^ ^33^, ^34^ ^]^ chemical asymmetry,^[^ ^35^, ^36^ ^]^ host environment,^[^ ^37^ ^]^ and deposition techniques.^[^ ^38^ ^]^ Initially, it was believed that heteroleptic Ir(III) complexes, due to the structural differences between the chromophoric and the ancillary chelates, may exhibit better EDO than homoleptic counterparts, as they exhibit a more pronounced geometric and chemical asymmetry.^[^ ^39^, ^40^, ^41^ ^]^ For heteroleptic Ir(III) complexes comprising two chromophoric and one ancillary chelates, it has been observed that a preferred EDO is achieved when the angle between the TDMs and the C_2_ axis of the molecule is close to 90 degrees, and this angle can be controlled by incorporating bulky substituents on the chromophoric chelates.^[^ ^35^, ^42^, ^43^, ^44^ ^]^ Moreover, the structure of ancillary chelate is known to influence the EDO, although the specific mechanism underlying this effect remains unclear.^[^ ^45^ ^]^ Recently, Kim et al. demonstrated that the EDO of homoleptic complexes can also be manipulated by incorporating bulky aromatic appendages on the chelates.^[^ ^46^ ^]^ They found that the extension of chelates can affect the angle between TDMs and the C_3_ axis of the complex, thus influencing the EDO. Furthermore, Schmidt et al. and Jung et al. proposed that the aspect ratio and electrostatic surface potential also play important roles in forming the well‐aligned homoleptic complexes.^[^ ^34^, ^36^ ^]^

In recent years, our group has developed a series of charge‐neutral asymmetric Ir(III) complexes bearing a tridentate bis‐N‐heterocyclic carbene (bis‐NHC) pincer chelate (2,6‐bisimidazolylidene benzene).^[^ ^47^, ^48^, ^49^, ^50^, ^51^, ^52^ ^]^ Their outstanding performance has been demonstrated in the preparation of efficient blue emitters^[^ ^53^, ^54^ ^]^ and phosphorescence sensitizers of OLED devices.^[^ ^55^, ^56^ ^]^ Compared to typical tris‐bidentate Ir(III) complexes, these asymmetric Ir(III) complexes may display a higher degree of geometric and chemical anisotropy. This inherent molecular advantage could further enhance the emitting dipole orientations of Ir(III) complexes.

**Figure 1:**
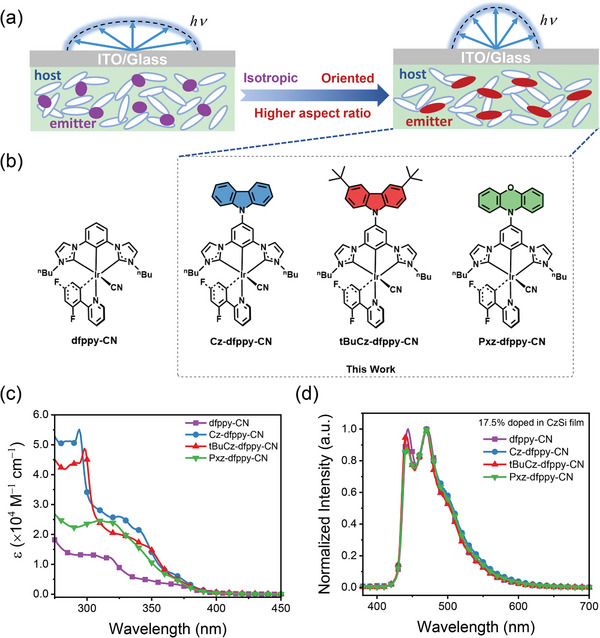
a) Illustration of the impact of molecular orientation on light outcoupling of OLED device. b) Chemical structures of the studied Ir(III) complexes. c) UV–vis absorption spectra recorded in dichloromethane (DCM) solution. d) PL spectra of the Ir(III) complexes doped in host material CzSi on fused silica substrate (conc. 17.5 wt%).

Herein, we rationally designed and synthesized three novel asymmetric Ir(III) complexes, namely **Cz‐dfppy‐CN**, **tBuCz‐dfppy‐CN**, and **Pxz‐dfppy‐CN**. These complexes feature different electron‐donating groups (EDGs) on the tridentate dicarbene pincer chelate. As is depicted in **Figure** **1**a, this chemical modification is to enlarge the aspect ratio of the molecular structures for these newly designed complexes, thus inducing the preferred emitting dipole orientation of the emitters. Our newly synthesized emitters exhibited enhanced horizontal transition dipole ratio (Θ_//_), with values of 0.73 for **dfppy‐CN** versus 0.89 and 0.90 for **Cz‐dfppy‐CN** and **tBuCz‐dfppy‐CN**, respectively. Additionally, **tBuCz‐dfppy‐CN** also exhibited a superior photoluminescence quantum yield (PLQY) of 0.95, as well as a short excited‐state lifetime of 2.13 µs. Owing to the favorable transition dipole orientation and high PLQY of the emitters, the PhOLED devices showed outstanding performance. Specifically, the PhOLED device using **tBuCz‐dfppy‐CN** as the emitter exhibited a saturated blue emission peak at 445 nm and a CIE color coordinate of (0.140, 0.148), with a superior maximum external quantum efficiency (EQE_max_) of 30.7%. Additionally, optical simulations of the PhOLED devices provide quantitative support for the discussion of enhanced outcoupling efficiency resulting from improvd EDO alignment. Furthermore, **tBuCz‐dfppy‐CN** was used as emitter for tandem OLED devices and sensitizer for hyper‐OLED devices. The tandem device exhibited a high EQE of 44.2% as well as small efficiency roll‐off. The hyper‐OLED showed narrowband emission with CIE coordinate of (0.120, 0.136), together with a maximum EQE of 31.4% and an operational lifetime to half of initial luminance (LT_50_) of 57.54 h at an initial brightness of 1000 cd m^‒2^. Therefore, this work has provided a rational strategy to reach highly efficient deep blue emission with operational stability, opening up a promising window to move forward blue phosphorescent OLEDs for high‐end display and lighting industry.

## 2 Results and Discussion

### 2.1 Materials Synthesis and Structure Determination

Figure 1b illustrates the molecular structures of these newly obtained Ir(III) complexes, in comparison with the control complex **dfppy‐CN**.^[^ ^47^ ^]^ The different electron‐donating groups (EDGs) on the dicarbene pincer chelates were highlighted by different colors. The detailed procedures of organic synthesis can be found in the supplementary information (Scheme S2, Supporting Information). The chemical composition of the Ir(III) complexes are characterized by ^1^H, ^19^F, and ^13^C nuclear magnetic resonance (NMR) spectra and high‐resolution electron‐spray ionization mass (ESI‐MS) spectra (Figures S25–S45, Supporting Information). To verify their stereochemical structures, the single crystal of **tBuCz‐dfppy‐CN** was examined by X‐ray diffraction analysis, to which the molecular structure is presented in Figure S1 (Supporting Information). Detailed information regarding the crystal determination, as well as selected bond lengths and angles, can be found in Table S1 and S2 (Supporting Information), respectively. Specifically, the bond lengths of Ir‐C_NHC_ are ≈2.06 Å and the C_NHC_‐Ir‐C_NHC_ bite angle is found to be 155.2°, which is close to that of similar crystal structures reported in literature.^[^ ^47^ ^]^ Notably, the dihedral angle between the di‐*tert*‐butyl carbazole (tBuCz) moiety and the dicarbene pincer chelate is calculated to be 63.12°, as depicted in Figure S2 (Supporting Information).

### 2.2 Photophysical Properties

The UV–vis absorption spectra of the studied Ir(III) complexes in CH_2_Cl_2_ and their photoluminescence (PL) emission spectra in thin films are presented in Figure 1c,d. The detailed photophysical data are summarized in **Table** **1**. In all four complexes, two absorption bands were observed at *ca*. 320–340 nm and 360–380 nm. The higher energy absorption bands were attributed to an admixture of spin allowed ligand‐centered ππ^*^ (^1^LC) transition and ligand‐to‐ligand charge transfer (^1^LLCT) transition, while the lower energy bands were believed to originate from the spin‐allowed metal‐to‐ligand charge transfer (^1^MLCT). Notably, the functional Ir(III) complexes exhibited increased extinction coefficient compared to **dfppy‐CN**, which could be attributed to the presence of intense narrow absorption peak at 294 nm (ε = 5.5 × 10^4^ M^−1^ cm^−1^) for **Cz‐dfppy‐CN** and 298 nm (ε = 4.9 × 10^4^ M^−1^ cm^−1^) for **tBuCz‐dfppy‐CN**. These absorption peaks were induced by π‐π^*^ transitions of Cz and tBuCz.^[^ ^57^, ^58^ ^]^ Additionally, a significant increase in the extinction coefficient of the absorption band ≈325 nm was observed for the three newly synthesized Ir(III) complexes compared with that of **dfppy‐CN**. This might originate from the enhanced state mixing of ligand‐centered and metal‐to‐ligand charge transfer facilitated by the introduction of EDGs, as predicted by the DFT calculation. The emission spectra of all four complexes measured in doped thin film (Figure 1d) exhibited similar well‐resolved vibronic bands, with two main peak maximums located at ca. 440 and 470 nm. Time‐resolved PL decay profiles (Figures S4 and S5, Supporting Information) were measured and the fitting results showed lifetimes of 2.72, 1.15, 2.13, and 5.66 µs for **dfppy‐CN**, **Cz‐dfppy‐CN, tBuCz‐dfppy‐CN**, and **Pxz‐dfppy‐CN**, respectively. Additionally, a significant improvement of PLQY was also observed for **tBuCz‐dfppy‐CN** (0.95) and **Cz‐dfppy‐CN** (0.88), outperforming that of **dfppy‐CN** (0.83). This originates from the reduced host guest interaction and enhanced state mixing of LC and MLCT excitations,^[^ ^59^ ^]^ as predicted by DFT calculation results (Figure S10 and Table S3, Supporting Information).

**Table 1:** Summarized photophysical properties of the studied Ir(III) complexes.

Complex	Medium	Absorption^a)^ λ_max_ [nm] [ε]	Emission				
			λ_max_ [nm]	τ_obs_ [µs]	Φ_PL_ ^b)^	k_r_ [×10^5^ s^−1^]	
dfppy‐CN	DCM	269(2.04), 317(1.23)	441, 468	2.87	0.67	2.33	
	17.5wt% in CzSi		444, 471	2.72	0.83	3.05	
Cz‐dfppy‐CN	DCM	294(5.51), 325(2.60)	439, 468	4.31	0.60	1.39	
	17.5wt% in CzSi		443, 470	1.15	0.88	7.22	
tBuCz‐dfppy‐CN	DCM	298(4.87), 330(1.99)	439, 467	3.84	0.74	1.92	
	17.5wt% in CzSi		441, 469	2.13	0.95	4.46	
Pxz‐dfppy‐CN	DCM	270(2.94), 310(2.45)	439, 469	1.14	0.023	0.20	
	17.5wt% in CzSi		442, 470	5.66	0.60	0.48	

^a)^ Recorded at a conc. of 2 × 10^−5^ M in DCM at R.T., extinction coefficient (ε) is in the unit of (10^4^ M^−1^ cm^−1^);

^b)^ *PLQY* (Φ_PL_) measured with Hamamatsu Quantaurus‐QY Absolute PL quantum yields measurement system.

### 2.3 Investigation of Molecular Orientation

**Figure 2:**
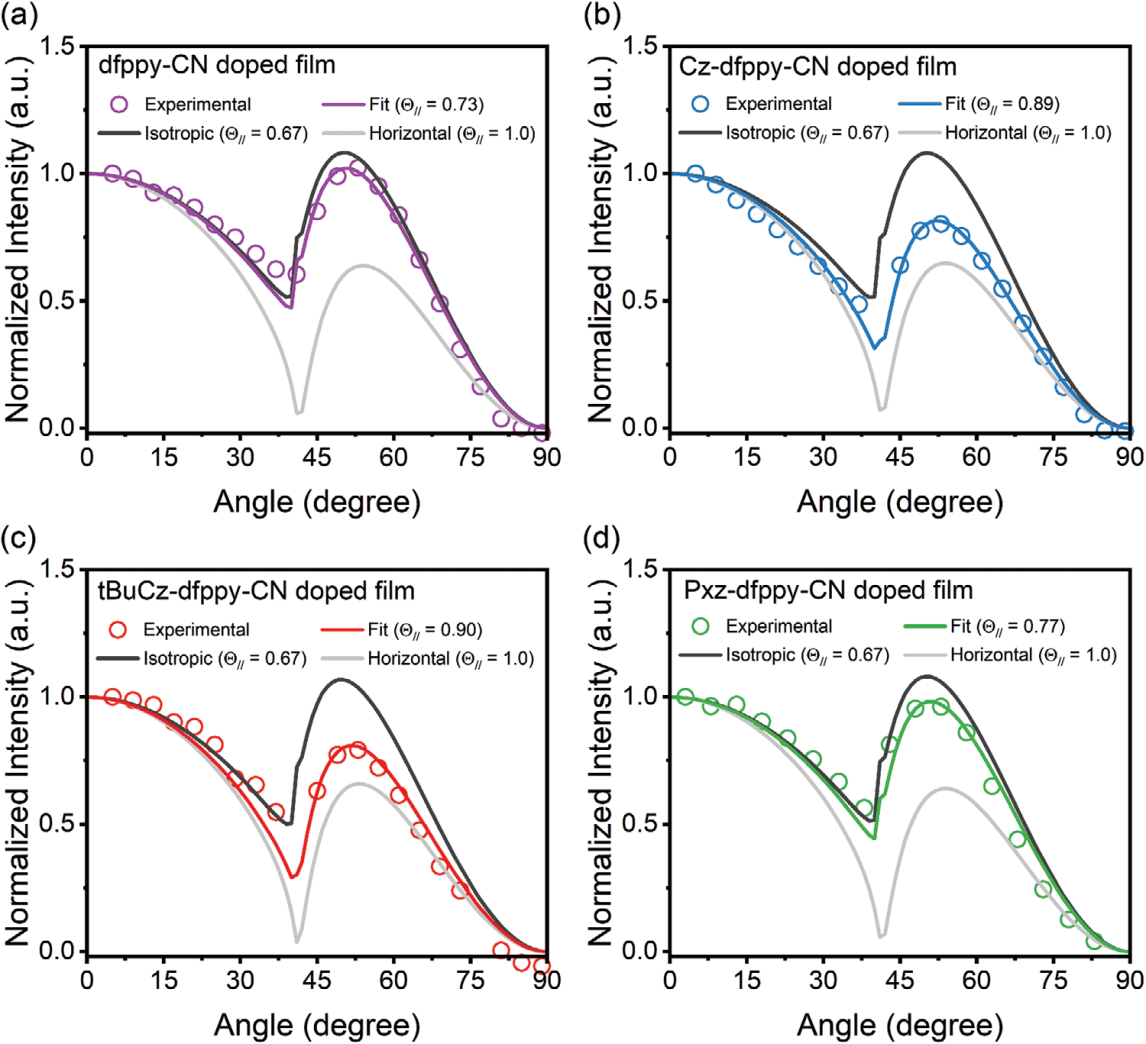
Angle dependent PL (ADPL) measurement shows the experimental and fitting result of horizontal dipole ratio (Θ_//_). From (a) to (d) are the respective Ir(III) complexes doped in CzSi films with a concentration of 17.5 wt%.

To characterize the molecular orientation of the Ir(III) complexes in host matrix, the horizontal dipole ratio (Θ_//_) was measured by angle‐dependent PL (ADPL) measurement of doped thin films.^[^ ^60^, ^61^ ^]^ The experimental data and fitting results of the *p*‐polarized PL intensity is depicted in **Figure** **2**, and the fitting values of Θ_//_ have been summarized in **Table** **2**. The Θ_//_ for **dfppy‐CN**, **Cz‐dfppy‐CN**, **tBuCz‐dfppy‐CN**, and **Pxz‐dfppy‐CN** were found to be 0.73, 0.89, 0.90, and 0.77, respectively. It is noteworthy that the Θ_//_ values for **Cz‐dfppy‐CN** and **tBuCz‐dfppy‐CN** are among the highest that ever reported in literatures for phosphors exhibiting favorable EDO.^[^ ^46^, ^62^, ^63^, ^64^ ^]^

**Table 2:** Summary of molecular aspect ratio (*γ*) and horizontal dipole ratio (Θ_//_).

Complex	Aspect Ratio [*γ*]	EDO [Θ_//_]
dfppy‐CN	1.16	0.73
Cz‐dfppy‐CN	1.44	0.89
tBuCz‐dfppy‐CN	1.49	0.90
Pxz‐dfppy‐CN	1.30	0.77

**Figure 3:**
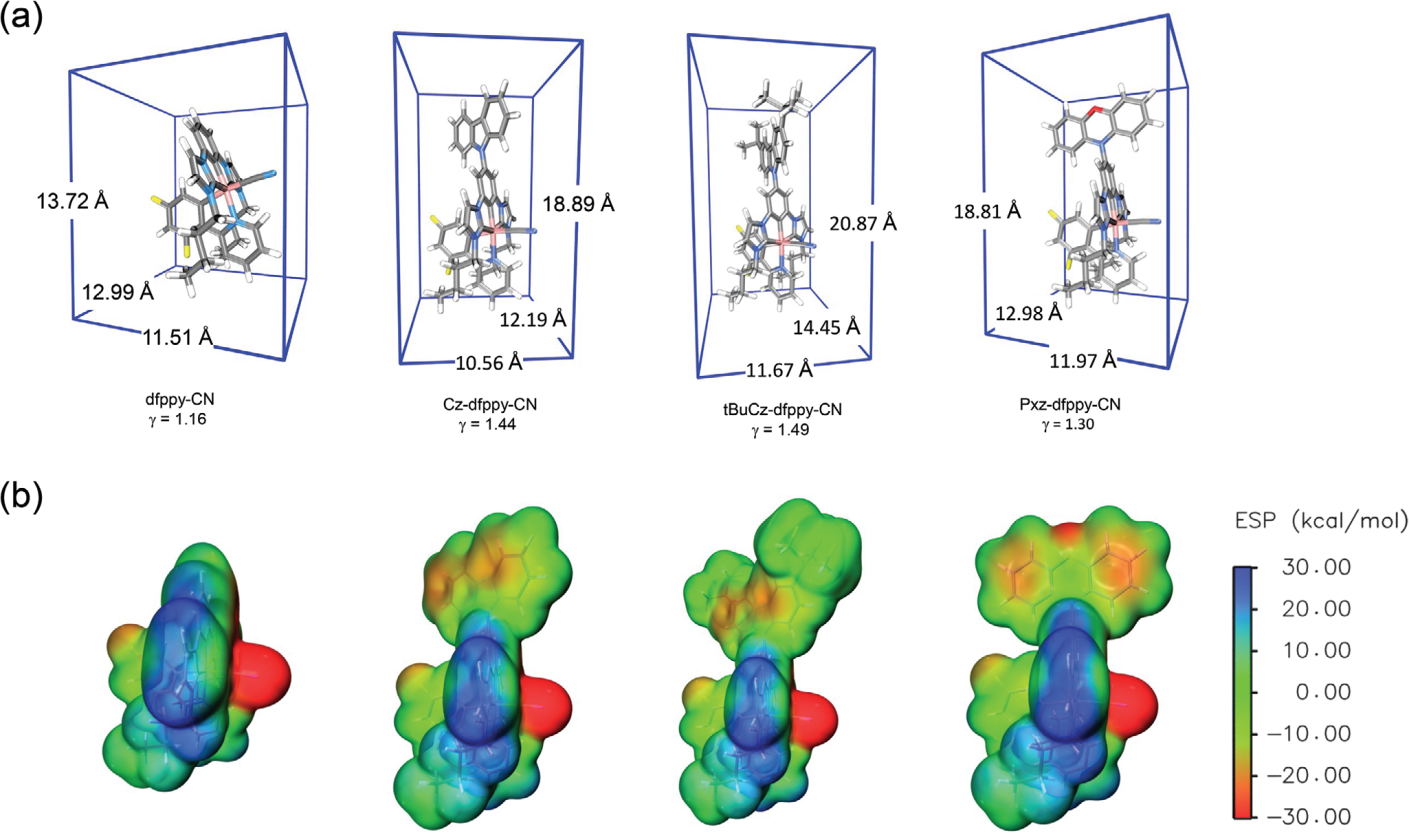
a) Size of the smallest bounding boxes of the Ir(III) complexes, calculated with optimized ground state (S_0_) geometries based on single crystal X‐ray structural data. Listed below are the aspect ratios (*γ*) of each molecule. b) Electrostatic potential distribution of the studied Ir(III) complexes at ground state (S_0_).

**Figure** **3**a illustrates the optimized geometry of these Ir(III) complexes in their ground state (S_0_), enclosed by their smallest bounding boxes. In this work, the aspect ratio (*γ*) was defined according to Schmidt et al., where $\gamma \;=\sqrt{a\cdot b}\;/c$, with *c* representing the shortest axis of the smallest bonding box, *a* and *b* representing the other 2 dimensions.^[^ ^36^ ^]^ The aspect ratios (*γ*) of the molecular structures are calculated to be 1.16, 1.44, 1.49, and 1.30 for **dfppy‐CN**, **Cz‐dfppy‐CN**, **tBuCz‐dfppy‐CN**, and **Pxz‐dfppy‐CN**, respectively. This trend shows that the incorporation of EDGs on the dicarbene pincer chelates has effectively increased the molecular geometric anisotropy. Notably, the observed trend of aspect ratio aligns well with the value of Θ_//_ determined by ADPL measurement, as summarized in Table 2, and a linear correlation between aspect ratio and EDO was observed (Figure S16, Supporting Information). Though the linear correlation between aspect ratio and EDO is significant for our asymmetric [3 + 2 + 1] Ir(III) complexes in the same host matrix, and has been observed in both homoleptic^[^ ^34^, ^36^ ^]^ and heteroleptic complexes,^[^ ^64^ ^]^ it is important to note that other factors such as inter‐molecular and dipole interactions between dopant and host could affect the orientation as well.^[^ ^16^, ^65^ ^]^

To explore the impact of inter‐molecular interactions on molecular orientation, the distribution of electrostatic surface potential (ESP) of all four complexes was obtained from their optimized structures and visualized as isovalue surface plots in Figure 3b. The maps of electrostatic potential revealed that, for all complexes, the ESP minima (indicated by the red region) were located near the nitrogen atom of the cyano ligand, while the ESP maxima were situated near the two nitrogen atoms in the NHC moiety. All four complexes exhibit high degree of chemical asymmetry, which is believed to contribute to the high Θ_//_ values of these asymmetric Ir(III) complexes.^[^ ^34^, ^36^, ^66^ ^]^ To investigate the potential interactions between dopant Ir(III) complexes and host molecules, the ESP of CzSi was also calculated based on its ground state geometry. The map of ESP for CzSi, as well as the studied Ir(III) complexes, were summarized in Figure S17 (Supporting Information). The tert‐butyl benzene moiety of CzSi host molecule exhibits positive ESP, while the monodentate cyano ligand group of as‐obtained asymmetric Ir(III) complexes exhibit negative ESP, resulting in strong van der Waals interaction between host and dopant molecules. The active area of the tridentate ligands are substantially increased after the bulky EDGs are incorporated, which facilitates their preferential orientation.^[^ ^44^ ^]^

In addition, the dihedral angles between the EDGs and the central benzene unit of dicarbene pincer chelate were investigated based on the optimized geometries of the Ir(III) complexes, which are depicted in Figure S9 (Supporting Information). The dihedral angle between Cz/tBuCz moiety and dicarbene pincer chelate is calculated to be ≈60°, agreeing with the angle of 63° obtained from the single crystal data discussed earlier (Figure S2, Supporting Information). However, the dihedral angle between Pxz and the dicarbene pincer chelate was found to be close to 90°, which may lead to the less pronounced aspect ratio of **Pxz‐dfppy‐CN**.

### 2.4 Electrochemistry and Thermal Stability

**Figure 4:**
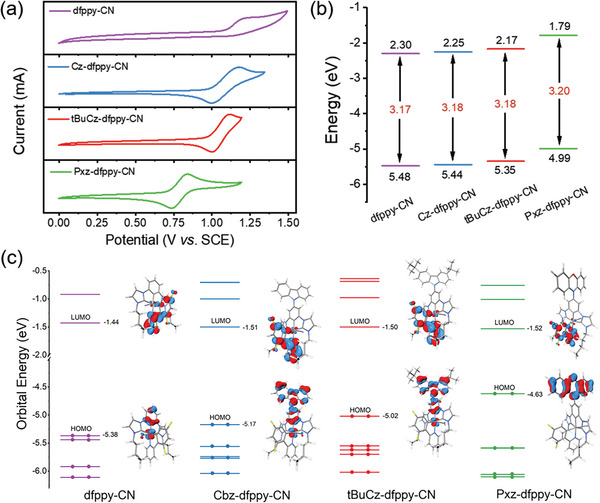
a) Cyclic voltammetry (CV) diagrams showing the oxidation peak potentials. b) Estimated HOMO and LUMO levels. c) Selected molecular orbitals of ground state (S_0_) from DFT calculation of the studied Ir(III) complexes.

The electrochemical properties of the complexes were investigated using cyclic voltammetry (CV), as shown in **Figure** **4**a. The oxidation peak potentials decreased in the order of **dfppy‐CN**, **Cz‐dfppy‐CN**, **tBuCz‐dfppy‐CN**, and **Pxz‐dfppy‐CN**. This trend could be attributed to the increase of electron‐donating ability of EDGs incorporated. Notably, all three Ir(III) complexes with EDGs on the dicarbene pincer chelates exhibited reversible oxidation peak except for **dfppy‐CN**, which displayed an irreversible process. This observation indicates that the introduced donor group may improve the electrochemical stability of the emitters. The HOMO and LUMO levels of the complexes were next estimated using the oxidation potential and absorption onset, as depicted in Figure 4b and summarized in **Table** **3**. The HOMO levels of three newly obtained Ir(III) complexes were found to be higher than that of **dfppy‐CN** (−5.38 eV). Furthermore, as the electron‐donating ability increased, the HOMO levels became shallower. Similarly, the LUMO levels follow the identical decending trend of HOMO levels, as the optical band gap estimated by absorption onset remain relatively constant among all complexes. This observation aligns well with the previously discussed emission properties, where the structures and emission peak wavelengths remained unchanged among all Ir(III) complexes.

**Table 3:** Oxidation potentials, estimated HOMO and LUMO energy levels, and decomposition temperatures of the studied Ir(III) complexes.

Complex	E_ox_ ^a)^ [V]	HOMO^b)^ [eV]	LUMO^c)^ [eV]	E_g_ ^d)^ [eV]	T_d_ ^e^ ^)^ [°C]
dfppy‐CN	1.10	−5.52	−2.40	3.17	394
Cz‐dfppy‐CN	1.09	−5.44	−2.26	3.18	402
tBuCz‐dfppy‐CN	1.05	−5.35	−2.16	3.18	426
Pxz‐dfppy‐CN	0.79	−4.99	−1.78	3.20	408

^a)^ The oxidation potential data were recorded in CH_2_Cl_2_ with 0.1 M nBu_4_NPF_6_ as electrolyte;

^b)^ E_HOMO_ levels were estimated from electrochemical potentials, i.e., E_HOMO_ = −e[E_pa_ +(4.8 − Fc^+^/Fc)] or E_HOMO_ = −e[E_1/2Ox_ + (4.8 − Fc^+^/Fc)], where Fc^+^/Fc = 0.46 V in CH_2_Cl_2_;

^c)^ E_LUMO_ = E_HOMO_ + Eg;

^d)^ Energy gap is achieved from the onset of the absorption, where Eg = 1240/ λ_absorp. onset_;

^e)^ Decomposition temperature of 5% weight loss.

Thermogravimetry analysis (TGA) was carried out to investigate the thermal stability of the complexes, as depicted in Figure S8 (Supporting Information). The corresponding decomposition temperatures (*T*_d_) were summarized in Table 3. The temperature to show a 5% weight loss occurred at 394, 402, 426, and 408 °C for **dfppy‐CN** and **Cz‐dfppy‐CN**, **tBuCz‐dfppy‐CN** and **Pxz‐dfppy‐CN**, respectively. Notably, the last three obtained Ir(III) complexes were found to exhibit higher *T*_d_ in comparison to **dfppy‐CN**. This finding suggests that the introduced EDGs have improved the thermal durability of the emitters, which is crucial for their endurance against heating during the vacuum thermal deposition of emitter in giving thin films.

### 2.5 Computational Studies

To gain a deeper understanding of the photophysical properties of these newly synthesized Ir(III) complexes, density functional theory (DFT) and time‐dependent density functional theory (TD‐DFT) calculations were performed. The frontier molecular orbitals of the optimized ground state (S_0_) are presented in Figure 4c, and the selected molecular orbitals around HOMO and LUMO are presented in Figures S11–S14 (Supporting Information). The calculated HOMO energy levels exhibit a trend similar to those estimated by the electrochemical method, confirming the effects of EDGs in alleviating the HOMO energies. Conversely, their calculated LUMO energy levels showed only a slight variation upon chemical modification. This is because that their LUMO orbitals are primarily localized on the bidentate C^N chelate, which remain unaltered for all four complexes. The simulated absorption spectra of these Ir(III) complexes are depicted in Figure S10 (Supporting Information). For **dfppy‐CN**, **Cz‐dfppy‐CN**, and **tBuCz‐dfppy‐CN**, the computed low energy absorption bands at ca. 370 nm are primarily attributed to HOMO−1 → LUMO transitions. However, the calculated absorption band of **Pxz‐dfppy‐CN** originates from the mixed HOMO−2 → LUMO and HOMO−1 → LUMO transitions, to which their contributions are approx. identical. Moreover, the LUMO orbitals of these Ir(III) complexes are mainly spreading over the π^*^ orbitals of the bidentate C^N chelate, while the HOMO−1 orbitals are primarily localized on the dicarbene pincer chelate and Ir(III) metal based *d* orbitals. Therefore, their lowest energy absorption tail at ca. 360−400 nm can be attributed to a mixture of ^1^MLCT[d(Ir) → π^*^(C^N)] and ^1^LLCT[π(C^C^C) → π^*^(C^N)] charge transfer transitions. In contrast, the computed higher energy absorption band at ca. 325 nm is predominantly contributed by HOMO−2 → LUMO transition for **dfppy‐CN**, HOMO−4 → LUMO transition for **Cz‐dfppy‐CN** and **tBuCz‐dfppy‐CN** and HOMO−3 → LUMO transition for **Pxz‐dfppy‐CN**. To study the luminescence mechanism of the Ir(III) complexes, natural transition orbital (NTO) analysis was conducted to pinpoint the nature of S_0_→T_1_ transitons,^[^ ^67^ ^]^ and the results were depicted in Figure S15 (Supporting Information). Across all four complexes, the NTO pair showed similar distribution, indicating that the S_0_→T_1_ predominatly originates from the admixture of ligand‐centered (LC) ππ^*^ transition and metal‐to‐ligand charge transfer (MLCT). This aligns well with the structured vibronic emission bands of the PL and EL spectra, as structureless emission is typically observed in Ir(III) complex showing strong MLCT and LLCT characteristics.^[^ ^53^, ^68^ ^]^ The fraction of MLCT was estimated by the difference between the contribution of the iridium core in the hole and electron NTO.^[^ ^21^, ^69^ ^]^ The contribution of MLCT was calculated as 14.21%, 13.34%, 13.25%, and 12.77% for **dfppy‐CN**, **Cz‐dfppy‐CN**, **tBuCz‐dfppy‐CN,** and **Pxz‐dfppy‐CN** respectively.

### 2.6 Electroluminescence

**Figure 5:**
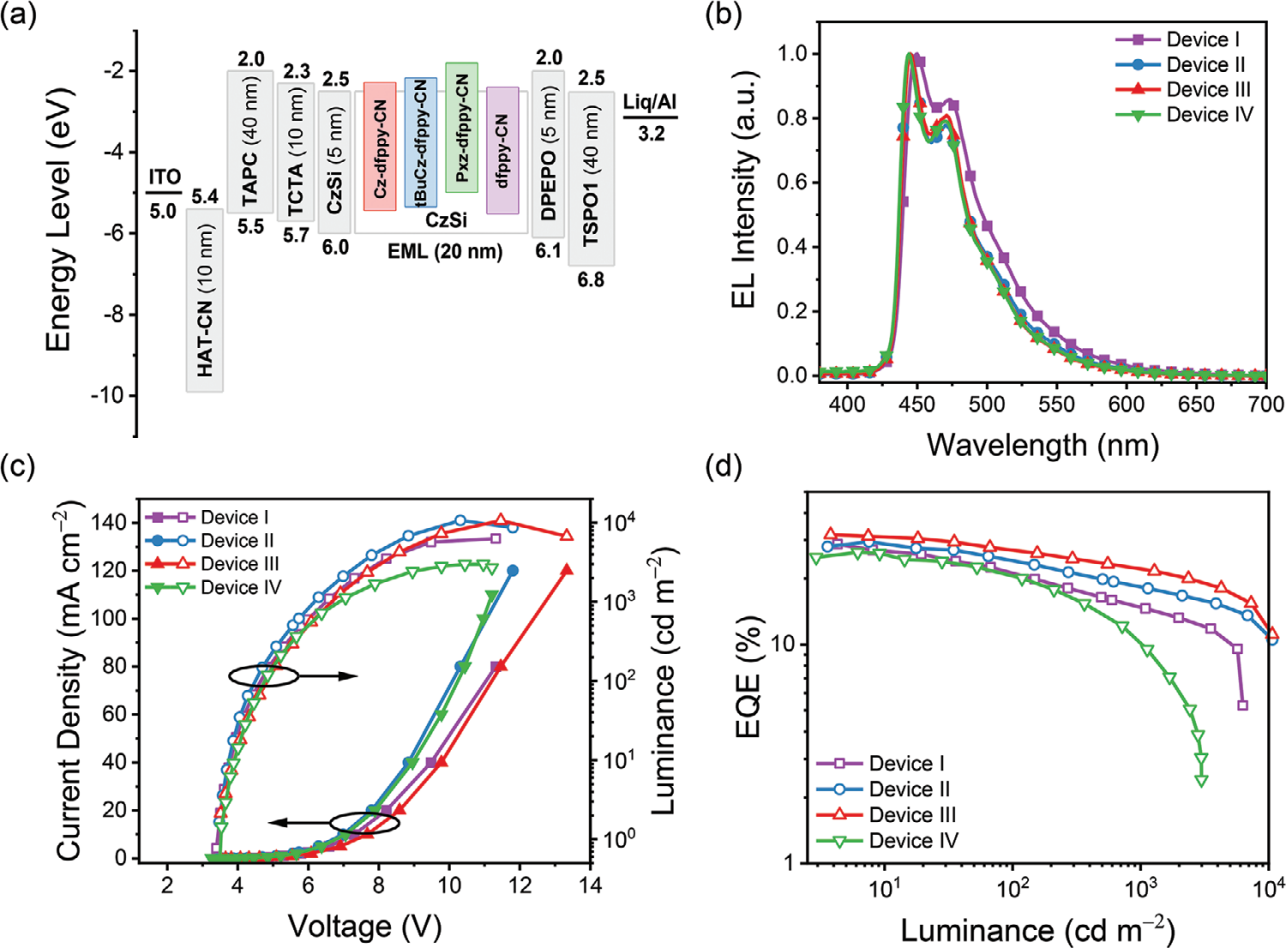
a) Device structure of the studied PhOLED devices. b) Electroluminescence (EL) spectra. c) *J−V−L* characteristics. d) EQE versus Luminance plot. Device I: 17.5 wt% of dfppy‐CN; Device II: 17.5 wt% of Cz‐dfppy‐CN; Device III: 17.5 wt% of tBuCz‐dfppy‐CN; Device IV: 15 wt% of Pxz‐dfppy‐CN.

Utilizing the merits of these novel asymmetric Ir(III) complexes, such as high PLQY, ideal thermal stability and preferred EDO, blue PhOLED devices were fabricated. The acronyms and chemical structures of materials used in the PhOLED device are shown in Scheme S1 (Supporting Information), and the device structure is depicted in **Figure** **5**a. Specifically, 9‐(4‐*tert*‐butylphenyl)−3,6‐bis(triphenylsilyl)−9H‐carbazole (CzSi) was chosen as the host material for its high morphological stability and high triplet energy.^[^ ^70^ ^]^ Additionally, a thin layer of bis[2‐(diphenylphosphino)phenyl]ether oxide (DPEPO) was inserted between the emission layer (EML) and the electron transporting layer (ETL) to block excessive hole transportation. The EL spectra, J‐V‐L characteristics, and EQE versus luminance plot are depicted in Figure 5b–d, while their numerical performance data are summarized in **Table** **4**. Device optimization data with all emitter doping concentrations has been summarized in Table S8 (Supporting Information), and their performance were graphically illustrated in Figures S18–S21 (Supporting Information). The electroluminescence (EL) spectra of these devices exhibited well‐resolved vibronic bands similar to PL spectra (Figure 1a) of the emitters in thin films. Compared with Device I (**dfppy‐CN**), Device II‐IV displayed a slight blue‐shift in the emission peaks and smaller full width half maximum (FWHM) compared to Device I (**dfppy‐CN**), which may be attributed to the reduced intermolecular π‐π stacking that mitigated host‐guest interactions resulting from the incorporation of bulky Cz/tBuCz substituents.^[^ ^59^ ^]^ As a result, Device II‐IV displayed smaller CIE_y_ coordinates compared with that of Device I (**dfppy‐CN**). Both Cz‐dfppy‐CN and tBuCz‐dfppy‐CN based device displayed optimal device performance at 17.5% doping concentration. With increasing fraction of emitters in EML, the turn‐on voltage decreased and the EQE increased significantly, as the increasing doping concentration of Ir(III) complexes facilitates the capture of injected excitons. In addition, the FWHM of the EL spectra increased slightly due to dopant aggregation at high doping concentration.^[^ ^71^ ^]^ However, the device performance dropped after the doping concentration reached 20%. This might be attributed to the increased triplet‐triplet annihilation (TTA) and triplet polaron quenching (TPQ). Notably, the maximum current efficiency (CE), power efficiency (PE), and EQE of 36.7 cd A^−1^, 32.6 Im W^−1^ and 30.7% were achieved for Device III (**tBuCz‐dfppy‐CN**), which are exceptionally high for saturated‐blue PhOLED with the CIE coordinate of (0.140, 0.148). Furthermore, Device II (**Cz‐dfppy‐CN**) showed a smaller CIE_y_ coordinate of 0.135 while showing a EQE_max_ of 29.5% at the same time. Compared with Device I (**dfppy‐CN**), Device II‐III exhibited smaller efficiency roll‐off, partly due to the shorter excited‐state lifetime of doped **Cz‐dfppy‐CN** and **tBuCz‐dfppy‐CN**. The larger efficiency roll‐off and inferior EQE of Device IV might be attributed to the longer excited‐state lifetime and low PLQY of **Pxz‐dfppy‐CN**. Additionally, it has also been reported in thermally activated delayed fluorescence (TADF) materials that the Pxz moiety may impose adverse impact on the stability of both the emitters and associated devices.^[^ ^72^, ^73^, ^74^ ^]^ The undesired photophysical properties of **Pxz‐dfppy‐CN**, together with the inherent instability of Pxz moiety, might explain the inferior performance of Device IV.

**Table 4:** EL performance of phosphorescence OLED devices using the studied Ir(III) complexes, along with tandem and hyper‐OLED performance.

Dopant in CzSi (Device)	V_on_ ^a)^ [V]	L_max_ [cd m^−2^]	max. EQE/CE/PE [%/cd A^−1^/Im W^−1^]	EQE/CE/PE @1000 cd m^−2^	λ_EL_ [nm]	FWHM^b)^ [nm]	CIE^c)^ [x, y]
17.5% dfppy‐CN (Device I)	3.4	6285	25.8/40.4/33.9	15.4/22.2/10.8	450/473	56.6	0.145, 0.178
17.5% Cz‐dfppy‐CN (Device II)	3.4	10 677	29.5/37.8/32.2	18.4/23.3/11.9	445/471	49.5	0.143, 0.135
17.5% tBuCz‐dfppy‐CN (Device III)	3.5	10 709	30.7/36.7/32.6	21.2/25.2/11.2	445/471	49.1	0.140, 0.148
15% Pxz‐dfppy‐CN (Device IV)	3.5	2985	24.3/28.4/23.1	10.2/12.1/5.6	444/471	48.8	0.143, 0.142
Tandem (Device V)	8.0	3230	44.2/61.4/17.9	40.0/53.9/10.3	466	26.5	0.136, 0.184
10% tBuCz‐dfppy‐CN: 1% ν‐DABNA (Device VI)	2.7	4196	31.4/26.1/23.4	21.7/19.2/9.3	470	17.1	0.120, 0.136

^a)^ Recorded at the luminance of 1 cd m^−2^;

^b)^ Full width half maximum;

^c)^ Recorded at the current density of 10 mA cm^−2^.

**Figure 6:**
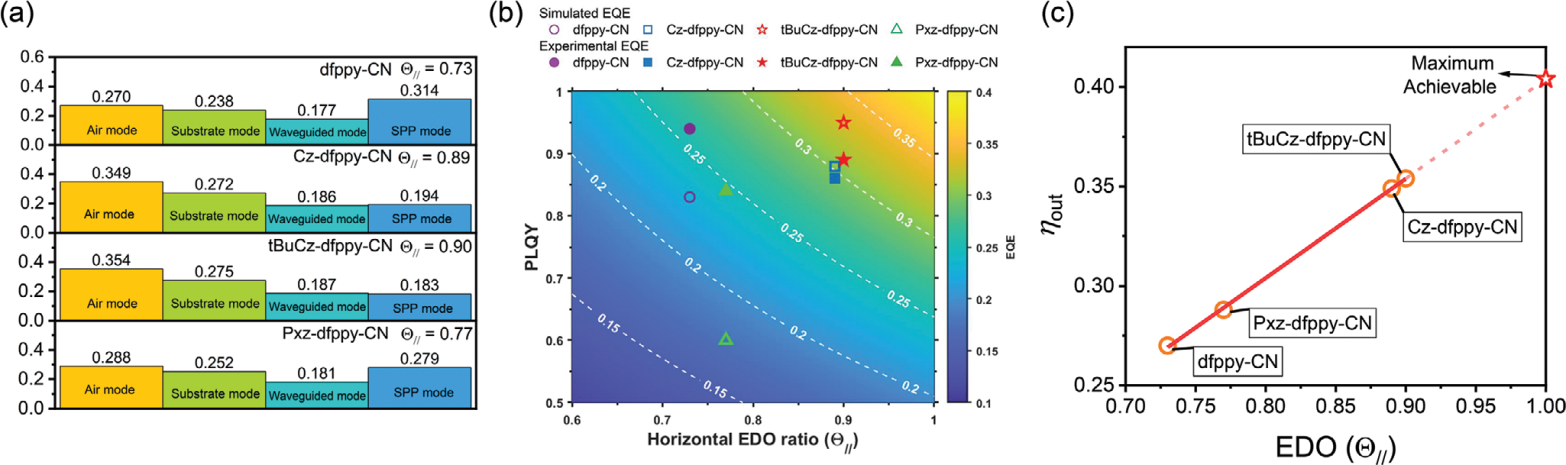
a) Simulated outcoupling efficiency (air mode) and the probability of light dissipated to other modes. b) Simulated maximum achievable EQE values presented as a contour plot, with a color bar representing the estimated EQE values. c) Calculated outcoupling efficiency (*η*_out_) plotted against Θ_//_, the dashed line is the projection to the maximum achievable value of *η*_out_.

To further quantify the impact of enhanced EDO on outcoupling efficiency and EQE, modal power dissipation analysis was conducted using the classical dipole model and transfer matrix method.^[^ ^75^, ^76^, ^77^ ^]^ The optical model incorporated Θ_//_ values are obtained from ADPL measurement. The optical constants of all materials used were obtained using variable angle spectroscopic ellipsometry (VASE) and were presented in Figure S7 (Supporting Information). The outcoupling efficiency (*η*_out_), as is represented by air mode in **Figure** **6**a, was calculated to be 0.270, 0.349, 0.354, and 0.288 for Device I‐IV, respectively. These results indicate that the improvement of molecular packing orientation has led to an increase in the outcoupling efficiency. Furthermore, theoretically achievable values of *EQE* were calculated and visualized as contour map shown in Figure 6b. The experimental *EQE*s and the corresponding simulated values were marked out. The result showed an ideal match between experimental and simulated *EQE*s for Devie II (**Cz‐dfppy‐CN)** and Device III (**tBuCz‐dfppy‐CN**). In Figure 6c the calculated *η*_out_ was also plotted against Θ_//_ and was projected to Θ_//_ = 1, which is the case where all emitters possess horizontally arranged EDO. The theoretical maximum *η*_out_ for the studied device structure is predicted to be over 0.40, showing the potential for further improvement. Overall, considering both experimental and simulation data, a significant improvement in *EQE* was observed for Device II‐III compared with Device I, which can be primarily attributed to the enhancement in both molecular packing orientation and photophysical properties of the emitters.

To demonstrate the excellent electroluminescence performance of the obtained Ir(III) complexes, tandem, and hyper OLED devices were also fabricated using **tBuCz‐dfppy‐CN** as the emitter. The numerical data of tandem and hyper‐OLED performance was summarized in Table 4, and the EL spectra, efficiency roll‐off, and *J‐V‐L* characteristics were depicted in Figure S23 (Supporting Information). Their energy level configuration is depicted in Figure S22 (Supporting Information). The tandem OLED device consists of two light‐emitting units separated by a 10 nm thick of Bphen:Yb (1 wt%) layer as the intermediate connector. Due to the microcavity effect of the tandem device, the original structured peak profile was modulated to a structureless peak profile centered at 466 nm, and the FWHM was narrowed down to 26.5 nm, leading to a CIE coordinate of (0.136, 0.184). Also due to the theoretically doubled current efficiency (CE), the tandem device demonstrated a high maximum EQE of 44.2%, as well as a small efficiency roll‐off of <10%. Furthermore, **tBuCz‐dfppy‐CN** was applied as a phosphorescence sensitizer to the terminal emitter ν‐DABNA in making the hyper‐OLED device. The device structure of hyper‐OLED is similar to that of PhOLEDs (Device I‐IV), except that the unstable DPEPO hole blocking layer was removed, and the SiCzCz:SiTrzCz2 cohost was introduced in the EML to achieve lower turn‐on voltage as well as improved device stability.^[^ ^59^ ^]^ Low concentration (1 wt%) of terminal emitter ν‐DABNA was doped in EML to mitigate Dexter energy transfer (DET) that may populate longlife triplet state of ν‐DABNA. Alternatively, the shortened phosphorescence lifetime and high *k*_r_ of hyper‐OLED have been facilitated by the efficient Förster resonant energy transfer (FRET) process.^[^ ^78^, ^79^ ^]^ The resulting hyper‐OLED devices demonstrated a narrowband emission at a peak wavelength of 470 nm with small FWHM of 17.1 nm, leading to a reduced CIE coordinate of (0.120, 0.136). Moreover, this hyper‐OLED device exhibited a high maximum EQE of 31.4% as well as a converted operational lifetime LT_50_ of 57.54 h at the initial luminescence of 1000 cd m^−2^ (Figure S24, Supporting Information).

## 3 Conclusion

To summarize, we synthesized a series of asymmetric Ir(III) complexes featuring bulky electron‐donating group on the dicarbene pincer chelate. The newly obtained complexes **Cz‐dfppy‐CN** and **tBuCz‐dfppy‐CN** showed a significant improvement in emitting dipole orientation compared with their parent complex **dfppy‐CN**, with horizontal transition dipole ratio ranging from 0.89 to 0.9. This improvement can be partially attributed to the increase in molecular aspect ratio and the inherent chemical (packing) anisotropy of the studied asymmetric Ir(III) complexes. Though the decisive factors for preferential molecular orientation require further investigation, the blue PhOLED device bearing **tBuCz‐dfppy‐CN** as the emitter achieved an impressive maximum *EQE* of 30.7% while maintaining a saturated‐blue emission with a CIE(x,y) color coordinate of (0.140, 0.148). The tandem blue OLED and hyper‐OLED devices featuring **tBuCz‐dfppy‐CN** also exhibited high efficiency, low roll‐off as well as enhanced device operational lifetime. This work offers potential guidance for future investigation of device applications of asymmetric Ir(III) complexes that display preferential emitting dipole orientations.

## Conflict of Interest

The authors declare no conflict of interest.

## Supporting information

Supporting Information

## Data Availability

Research data are not shared.
