# Supplementary material for: Enhanced Emitting Dipole Orientation Based on Asymmetric Iridium(III) Complexes for Efficient Saturated‐Blue Phosphorescent OLEDs
Source: Adv Sci (Weinh). 2024 Aug 13;11(38):2402349. doi: 10.1002/advs.202402349 (PMC11481260; doi:10.1002/advs.202402349)
Supplement: Supplementary file 1 — Supporting Information [file ADVS-11-2402349-s001.docx]

**Supporting Information**

**Enhanced Emitting Dipole Orientation Based on Asymmetric Iridium(III) Complexes for Efficient Saturated-Blue Phosphorescent OLEDs**

*Kefei Shi^#^, Chengcheng Wu^#^, He Zhang, Kai-Ning Tong, Wei He, Wansi Li, Zhaoyun Jin, Sinyeong Jung, Siqi Li, Xin Wang, Shaolong Gong, Yuewei Zhang, Dongdong Zhang, Feiyu Kang, Yun Chi^*^, Chuluo Yang^*^ and Guodan Wei**

# K. Shi and C. Wu contributed equally to this work.

K. Shi, C. Wu, W. Li, S. Jung, G. Wei

Tsinghua-Berkeley Shenzhen Institute (TBSI), Tsinghua University, Shenzhen 518055, China

K. Shi, C. Wu, K.-N. Tong, W. He, Z. Jin, S. Jung, S. Li, X. Wang, F. Kang, G. Wei

Institute of Materials Research, Tsinghua Shenzhen International Graduate School, Tsinghua University, Shenzhen 518055, China

H. Zhang, S. Gong

Hubei Key Lab on Organic and Polymeric Optoelectronic Materials, Department of Chemistry, Wuhan University, Wuhan 430072, China

Y. Zhang

*Laboratory of Flexible Electronics Technology, Tsinghua University, Beijing, China*

*D. Zhang*

*Key Laboratory of Organic Optoelectronics and Molecular Engineering of Ministry of Education, Department of Chemistry, Tsinghua University, Beijing, China*

Y. Chi

*Department of Materials Science and Engineering, Department of Chemistry, and Center of Super-Diamond and Advanced Films (COSDAF), City University of Hong Kong, Hong Kong SAR 999077, China*

C. Yang

Shenzhen Key Laboratory of New Information Display and Storage Materials, College of Materials Science and Engineering, Shenzhen University, Shenzhen 518060, China

Correspondence:

weiguodan@sz.tsinghua.edu.cn (G. Wei);

clyang@szu.edu.cn (C. Yang);

yunchi@cityu.edu.hk (Y. Chi)

Table of Contents

Table of Contents 1

List of Figures and Tables 2

1. X-ray crystallography 5

2. Photophysical Measurements 9

3. Thermogravimetric Measurement 14

4. Computational Details 15

5. Device Optimization 31

6. Experimental Section 36

7. Material Synthesis 38

8. NMR Spectra 49

9. ES-MS of Ir(III) complexes 58

Reference 61

List of Figures and Tables

**Scheme S1.** Acronyms, IUPAC names and molecular structures of the materials used in the studied OLED devices. 31

**Scheme S2.** General synthesis procedure of the [3+2+1] Ir(III) complexes. (i) Cs_2_CO_3_, (R = Cz, tBuCz and Pxz), DMF, 150 ℃ 48h; (ii) CuO, K_2_CO_3_, DMSO, 150 ℃; (iii) MeCN, 90 ℃; (iv) [Ir(cod)Cl]_2_, MeCN, 90 ℃; (v) dfppy, propionic acid, 150 ℃; (vi) AgCN, DMF, 100 ℃. 38

**Table S1.** Crystal determination data of complex **tBuCz-dfppy-CN.** 5

**Table S2.** Selected bond lengths and bond angles of complex **tBuCz-dfppy-CN.** 6

**Table S3.** Calculated first 15th singlet excited state energies, the associated oscillator strengths (*f*) and the nature of the transitions at the optimized ground state (S_0_) geometries of the four Ir(III) complexes in the Toluene by TD-B3LYP. The values in the parentheses are the % contributions of the specified configuration state function (CSF). 17

**Table S4.** Cartesian coordinates of the optimized ground-state (S0) and excited state (T1) geometry of **dfppy-CN**. 24

**Table S5.** Cartesian coordinates of the optimized ground-state (S0) and excited state (T1) geometry of **Cz-dfppy-CN.** 25

**Table S6.** Cartesian coordinates of the optimized ground-state (S0) and excited state (T1) geometry of **tBuCz−dfppyCN**. 27

**Table S7.** Cartesian coordinates of the optimized ground-state (S0) and excited state (T1) geometry of **Pxz-dfppy-CN**. 28

**Table S8.** Summarized Ph-OLED device optimization results. 33

**Figure S1.** Structure diagram of **tBuCz-dfppy-CN**. Thermal ellipsoids are shown at 30% probability level. Hydrogen atoms and solvent molecules were omitted for clarity. 7

**Figure S2.** Dihedral angle between di-*tert*-butyl carbazole (tBuCz) moiety and dicarbene pincer chelate obtained from the single crystal X-ray structural data of **tBuCz-dfppy-CN**. 8

**Figure S3.** PL spectra of the Ir(III) complexes in solvent of different polarity. 9

**Figure S4.** Transient PL decay profile of the Ir(III) complexes measured in CH_2_Cl_2_. 10

**Figure S5.** Transient PL decay profile of the Ir(III) complexes as co-deposited thin film (17.5 wt% doped in CzSi). 11

**Figure S6.** PL spectra of the Ir(III) complexes measured in toluene at 77 K and 300 K. 12

**Figure S7.** Refractive index of organic materials used in OLED device that were measured by variable angle spectroscopic ellipsometry (VASE). Red line represents refractive index (*n*) and blue line represents extinction coefficient (*k*). EML stands for emission layer (**tBuCz-dfppy-CN** doped in CzSi). Ellipsometry data *Ψ* and *Δ* were fitted using isotropic general oscillator model provided by CompleteEase^®^ program manufactured by J. A. Woollam. 13

**Figure S8.** The thermogravimetric analysis (TGA) curves of the studied Ir(III) complexes. 14

**Figure S9.** Optimized ground state S_0_ geometry of **Cz-dfppy-CN**, **tBuCz-dfppy-CN** and **Pxz-dfppy-CN**, and the dihedral angle between the electron-donating group and dicarbene pincer chelate. 16

**Figure S10.** Simulated UV-vis absorption spectra of the studied Ir(III) complexes computed by TDDFT/CPCM using toluene as solvent. The red vertical lines refer to the unbroadened oscillator strengths of the singlet-singlet transitions, and the black line is the fitting line of UV-Vis absorption (FWHM 0.03 eV). 19

**Figure S11.** Selected molecular orbitals of **dfppy-CN** at optimized ground state geometry (isovalue = 0.04). 20

**Figure S12.** Selected molecular orbitals of **Cz-dfppy-CN** at optimized ground state geometry (isovalue = 0.04). 20

**Figure S13.** Selected molecular orbitals of **tBuCz-dfppy-CN** at optimized ground state geometry (isovalue = 0.04). 21

**Figure S14.** Selected molecular orbitals of **Pxz-dfppy-CN** at optimized ground state geometry (isovalue = 0.04). 21

**Figure S15.** Natural transition orbital (NTO) pairs of S_0_→T_1_ transition of the studied Ir(III) complexes. 22

**Figure S16.** Correlation between the calculated aspect ratio and the emitting dipole orientation (EDO). 22

**Figure S17.** Maps of electrostatic potential (ESP) of the Ir(III) complexes and the host material CzSi. 23

**Figure S18.** (a) EL spectra; (b) J-V-L characteristics; (c) EQE vs. luminance plot of Ph-OLED featuring **dfppy-CN**. 32

**Figure S19.** (a) EL spectra; (b) J-V-L characteristics; (c) EQE vs. luminance plot of Ph-OLED featuring **Cz-dfppy-CN**. 32

**Figure S20.** (a) EL spectra; (b) J-V-L characteristics; (c) EQE vs. luminance plot of Ph-OLED featuring **tBuCz-dfppy-CN**. 32

**Figure S21.** (a) EL spectra; (b) J-V-L characteristics; (c) EQE vs. luminance plot of Ph-OLED featuring **Pxz-dfppy-CN**. 33

**Figure S22.** Device strucutre of (a) hyper-OLED (Device VI) and (b) tandem OLED (Device V). 34

**Figure S23.** (a) EL spectra, (b) J-V-L characteristics and (c) Efficiency vs. Luminance plot of tandem device (Device V) and hyper device (Device VI). 34

**Figure S24.** Operational lifetime of hyper-OLED using **tBuCz-dfppy-CN** as the sensitizer. 35

**Figure S25.** ^1^H NMR (400 MHz. CDCl3) of compound **Cz-dfppy-CN.** 49

**Figure S26.** ^19^F NMR (376 MHz, CDCl3) of compound **Cz-dfppy-CN.** 49

**Figure S27.** ^1^H NMR (400 MHz, CDCl3) of compound **Cz-dfppy-Br.** 50

**Figure S28.** ^1^H NMR (400 MHz. CDCl3) of compound **tBuCz-dfppy-CN.** 50

**Figure S29.** ^19^F NMR (471 MHz, CDCl3) of compound **tBuCz-dfppy-CN.** 51

**Figure S30.** ^1^H NMR (400 MHz, CDCl3) of compound **tBuCz-dfppy-CN.** 51

**Figure S31.** ^1^H NMR (400 MHz, CDCl3) of compound **Pxz-dfppy-CN.** 52

**Figure S32.** ^19^F NMR (471 MHz, CDCl3) of compound **Pxz-dfppy-CN.** 52

**Figure S33.** ^1^H NMR (400 MHz, CDCl3) of compound **Pxz-dfppy-Br.** 53

**Figure S34.** ^1^H NMR (400 MHz, DMSO−*d_6_*) of compound 9−(3,5−dibromophenyl)−9H−carbazole. 53

**Figure S35.** ^1^H NMR (400 MHz, DMSO−*d_6_*) of compound 9−(3,5−di(1H−imidazol−1−yl)phenyl)−9H−carbazole. 54

**Figure S36.** ^1^H NMR (400 MHz, DMSO−*d_6_*) of compound 1,1'−(5−(9H−carbazol−9−yl)−1,3−phenylene)bis(3−butyl−1H−imidazol−3−ium) dibromide (**Cz−pbib**). 54

**Figure S37.** ^1^H NMR (400 MHz, DMSO−*d_6_*) of compound 3,6−di−tert−butyl−9−(3,5−dibromophenyl)−9H−carbazole. 55

**Figure S38.** ^1^H NMR (400 MHz, DMSO−*d_6_*) of compound 3,6−di−tert−butyl−9−(3,5−di(1H−imidazol−1−yl)phenyl)−9H−carbazole. 55

**Figure S39.** ^1^H NMR (400 MHz, DMSO−*d_6_*) of compound 1,1'−(5−(3,6−di−tert−butyl−9H−carbazol−9−yl)−1,3−phenylene)bis(3−butyl−1H−imidazol−3−ium) (**tBuCz−pbib**). 56

**Figure S40.** ^1^H NMR (400 MHz, DMSO−*d_6_*) of compound 10−(3,5−dibromophenyl)−10H−phenoxazine. 56

**Figure S41.** ^1^H NMR (400 MHz, DMSO−*d_6_*) of compound 10−(3,5−di(1H−imidazol−1−yl)phenyl)−10H−phenoxazine. 57

**Figure S42.** ^1^H NMR (400 MHz, DMSO−*d_6_*) of compound 1,1'−(5−(10H−phenoxazin−10−yl)−1,3−phenylene)bis(3−butyl−1H−imidazol−3−ium) dibromide (**Pxz−pbib**). 57

**Figure S43.** ESI−MS of compound **Cz-dfppy-CN.** 58

**Figure S44.** ESI−MS of compound **tBuCz-dfppy-CN.** 59

**Figure S45.** ESI−MS of compound **Pxz-dfppy-CN.** 60

# X-ray crystallography

**Table S1.** Crystal determination data of complex **tBuCz-dfppy-CN.**

| **Complex** | **tBuCz-dfppy-CN** |
| --- | --- |
| Empirical formula | C_53_H_55_Cl_3_F_2_IrN_7_ |
| Formula weight | 1126.59 |
| Temperature/K | 193.00 |
| Crystal system | triclinic |
| Space group | P-1 |
| a/Å | 8.2761(5) |
| b/Å | 17.5567(11) |
| c/Å | 17.7849(10) |
| α/° | 99.102(3) |
| β/° | 96.197(4) |
| μ/mm^−1^ | 4.606 |
| F(000) | 1136.0 |
| Crystal size/mm^3^ | 1 × 0.5 × 0.2 |
| Radiation | GaKα (λ = 1.34138) |
| 2Θ (min to max)/° | 4.436 to 108.27 |
| μ/mm^−1^ | 4.606 |
| F(000) | 1136.0 |
| Crystal size/mm^3^ | 1 × 0.5 × 0.2 |
| Radiation | GaKα (λ = 1.34138) |
| Index ranges | −9 ≤ h ≤ 9, −20 ≤ k ≤ 21, −21 ≤ l ≤ 21 |
| Reflections collected | 28727 |
| Independent reflections | 9190 [R_int_ = 0.0487, R_sigma_ = 0.0506] |
| Data/restraints/parameters | 9190/6/603 |
| Goodness−of−fit on F^2^ | 1.047 |
| Final R indexes [I>=2σ (I)] | R_1_ = 0.0390, wR_2_ = 0.0906 |
| Final R indexes [all data] | R_1_ = 0.0504, wR_2_ = 0.0973 |
| Largest diff. peak/hole / e Å^−3^ | 0.84/−1.39 |

[a] R_1_= Σ||Fo| – |Fc|| / Σ|Fo|; [b] wR_2_= [Σw(|Fo| – |Fc|)2/ Σw|Fo|2]^1/2^

**Table S2.** Selected bond lengths and bond angles of complex **tBuCz-dfppy-CN.**

| **Complex** | **Bond Length/Angle** | **tBuCz-dfppy-CN** |
| --- | --- | --- |
| Bond Length (Å) | Ir1−C1 | 2.067(5) |
|  | Ir1−C4 | 1.958(5) |
|  | Ir1−C10 | 2.062(5) |
|  | Ir1−C21 | 2.048(4) |
|  | Ir1−N5 | 2.153(4) |
|  | Ir1−C32 | 2.046(5) |
| Bond Angles (degree) | C1−Ir1−N5 | 101.25(17) |
|  | C4−Ir1−N5 | 171.43(16) |
|  | C4−Ir1−C21 | 93.19(18) |
|  | C4−Ir1−C1 | 77.19(19) |
|  | C4−Ir1−C10 | 78.02(19) |
|  | C4−Ir1−C32 | 93.56(18) |
|  | C21−Ir1−N5 | 78.38(17) |
|  | C21−Ir1−C10 | 89.96(18) |
|  | C21−Ir1−C1 | 91.59(17) |
|  | C10−Ir1−N5 | 103.30(17) |
|  | C10−Ir1−C1 | 155.21(18) |
|  | C32−Ir1−N5 | 94.88(16) |
|  | C32−Ir1−C13 | 173.24(18) |
|  | C32−Ir1−C10 | 91.07(18) |
|  | C32−Ir1−C1 | 90.28(17) |


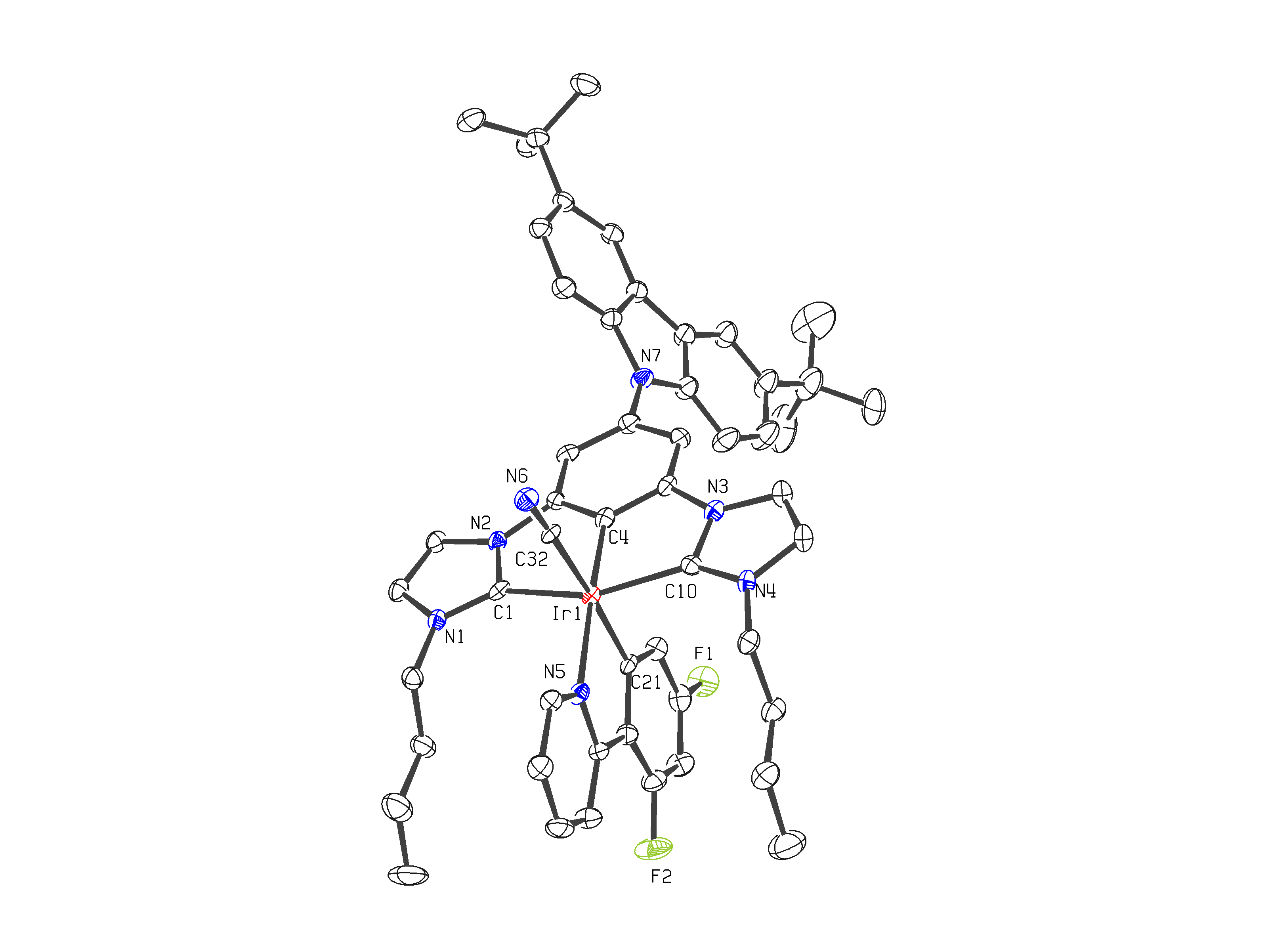


**Figure S1.** Structure diagram of **tBuCz-dfppy-CN**. Thermal ellipsoids are shown at 30% probability level. Hydrogen atoms and solvent molecules were omitted for clarity.


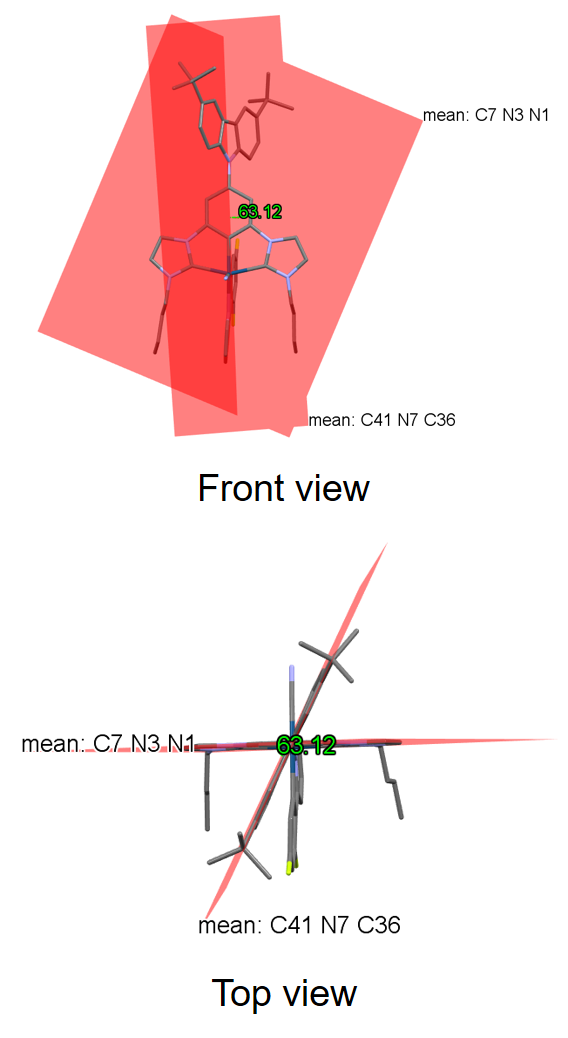


**Figure S2.** Dihedral angle between di-*tert*-butyl carbazole (tBuCz) moiety and dicarbene pincer chelate obtained from the single crystal X-ray structural data of **tBuCz-dfppy-CN**.

# Photophysical Measurements


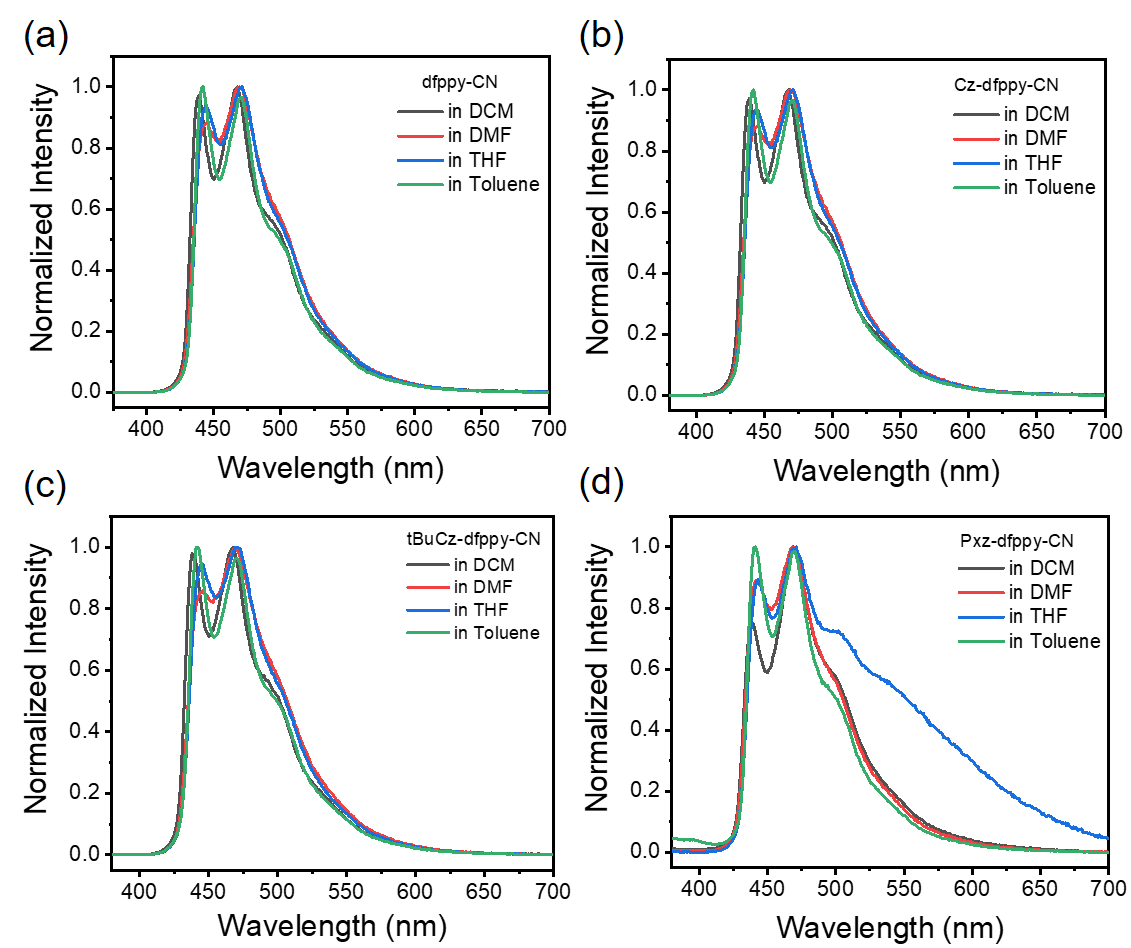


**Figure S3.** PL spectra of the Ir(III) complexes in solvent of different polarity.


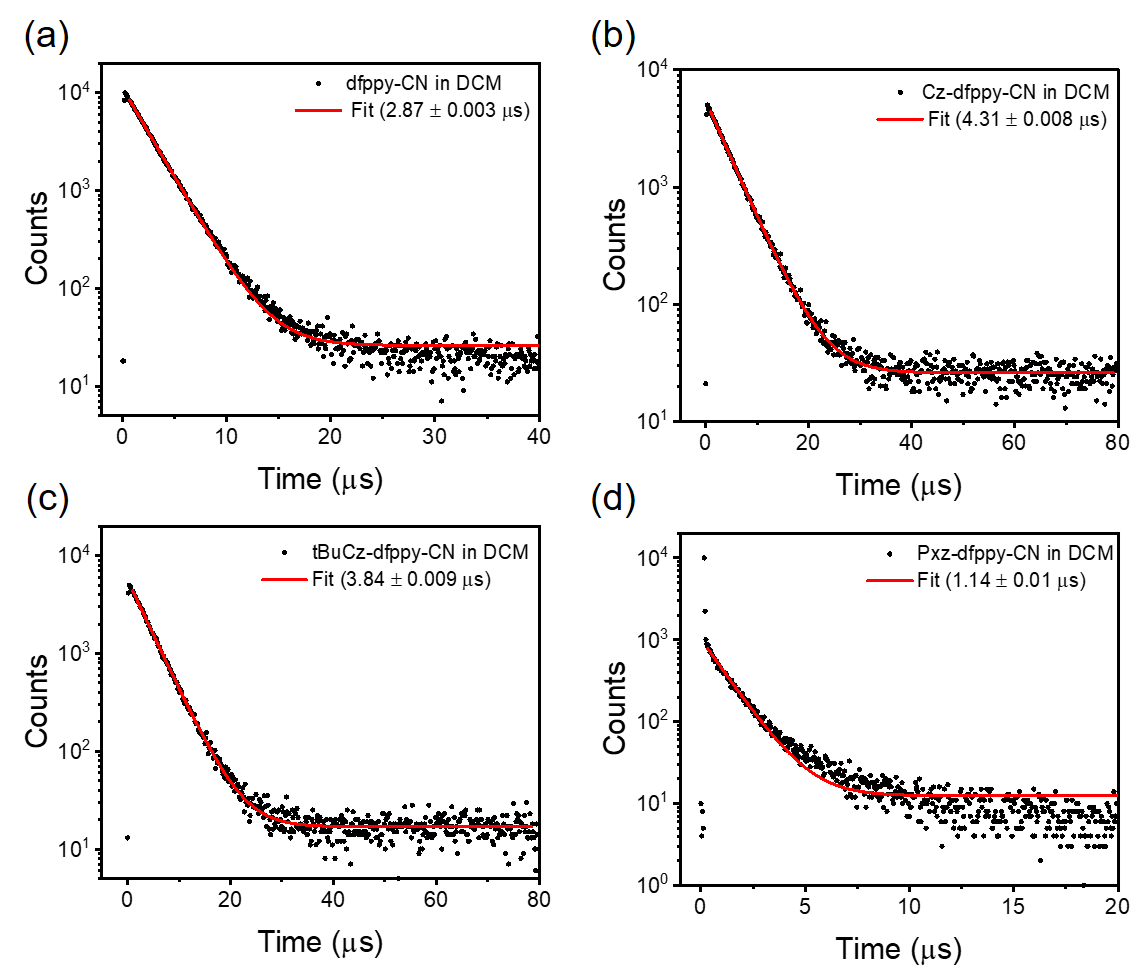


**Figure S4.** Transient PL decay profile of the Ir(III) complexes measured in CH_2_Cl_2_.


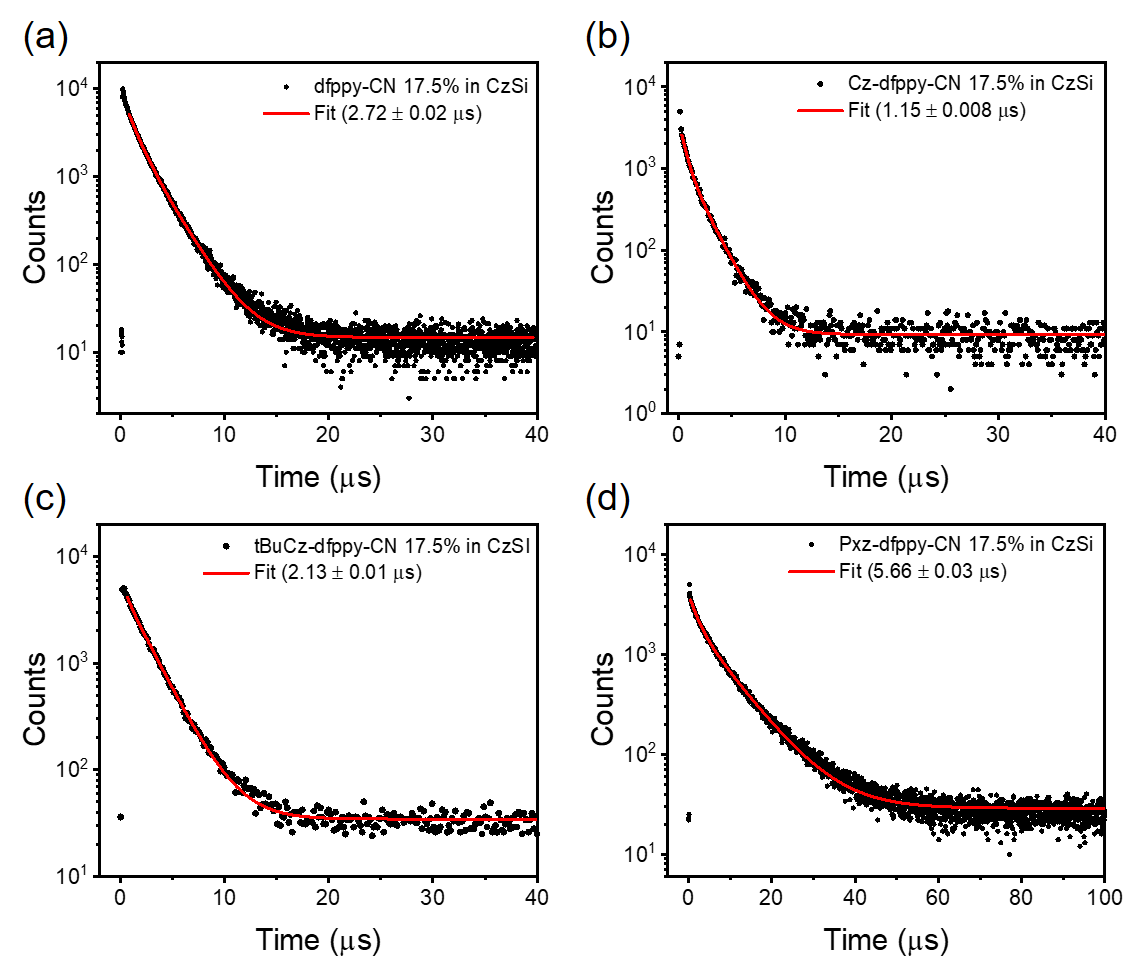


**Figure S5.** Transient PL decay profile of the Ir(III) complexes as co-deposited thin film (17.5 wt% doped in CzSi).


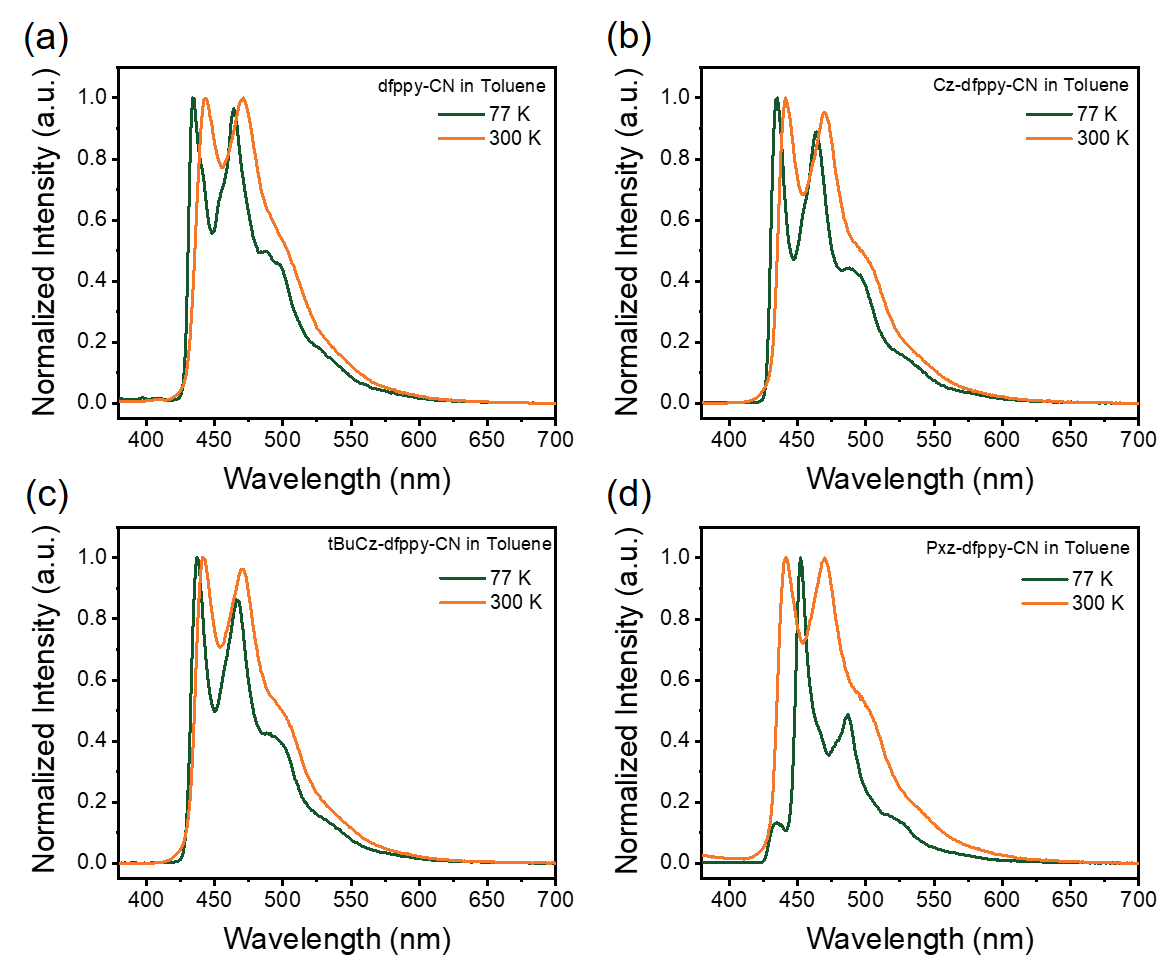


**Figure S6.** PL spectra of the Ir(III) complexes measured in toluene at 77 K and 300 K.

**Figure S7.** Refractive index of organic materials used in OLED device that were measured by variable angle spectroscopic ellipsometry (VASE). Red line represents refractive index (*n*) and blue line represents extinction coefficient (*k*). EML stands for emission layer (**tBuCz-dfppy-CN** doped in CzSi). Ellipsometry data *Ψ* and *Δ* were fitted using isotropic general oscillator model provided by CompleteEase^®^ program manufactured by J. A. Woollam.

# Thermogravimetric Measurement

**Figure S8.** The thermogravimetric analysis (TGA) curves of the studied Ir(III) complexes.

# Computational Details

All calculations were performed with the Gaussian 16 program suite.^[1]^ By density functional theory (DFT)^[2-3]^, the ground-state (S_0_) geometries of the Ir(III) complexes were fully optimized in toluene with the hybrid B3LYP functional,^[4-6]^ in conjunction with the conductor-like polarizable continuum model (CPCM).^[7-8]^ At the same level of theory, time-dependent density functional theory (TDDFT)^[9-10]^ calculations were performed on the optimized S0 geometries to compute the singlet-singlet transitions in the absorption spectra of the Ir(III) complexes. In order to investigate the emissive states of the complexes, the geometries of the lowest-lying triplet excited states (T1) have been optimized with the unrestricted UB3LYP/CPCM method. The vibrational frequencies of all the stationary points have been computed and all of them were verified as minima (NIMAG = 0) on the potential energy surface. The Cartesian coordinates of the optimized geometries of both the S0 and T1 states were given in Tables S4–S11. In all the calculations, the LanL2DZ basis set with effective core potential (ECP)^[11-13]^ was employed for Ir with f-type polarization functions (ζ= 0.938),^[14]^ whereas the 6-31G(d,p) basis set^[15-17]^ was applied to all other elements (C, H, and N). A pruned (99,590) grid was used for numerical integration. The electrostatic surface potential (ESP) analysis was conducted ESP evaluation algorithm employed by Multiwfn.^[18-19]^


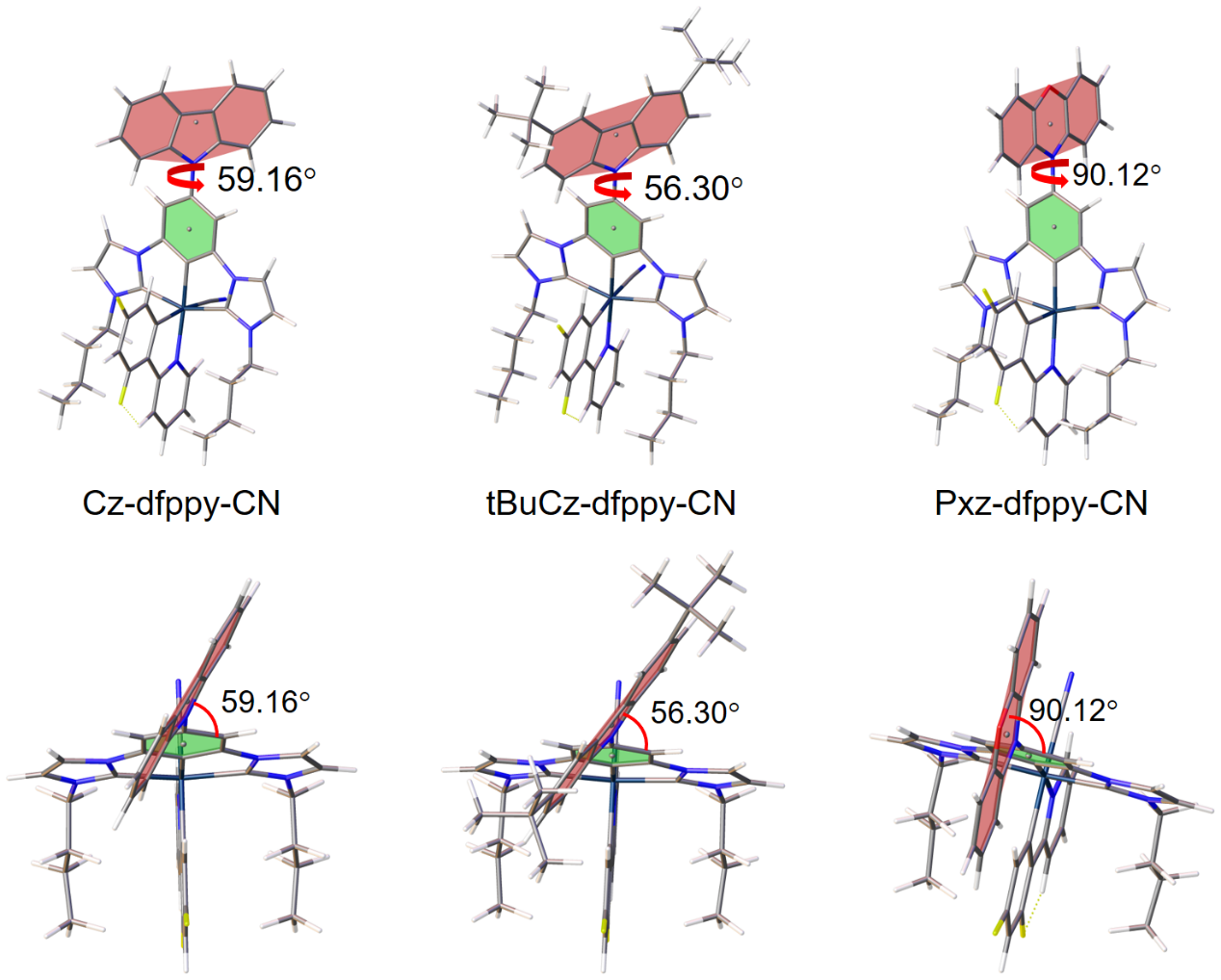


**Figure S9.** Optimized ground state S_0_ geometry of **Cz-dfppy-CN**, **tBuCz-dfppy-CN** and **Pxz-dfppy-CN**, and the dihedral angle between the electron-donating group and dicarbene pincer chelate.

**Table S3.** Calculated first 15th singlet excited state energies, the associated oscillator strengths (*f*) and the nature of the transitions at the optimized ground state (S_0_) geometries of the four Ir(III) complexes in the Toluene by TD-B3LYP. The values in the parentheses are the % contributions of the specified configuration state function (CSF).

| **dfppy-CN** | **Energy (eV)** | **λ (nm)** | **Oscillator Strength (*f*)** | **Major contributions** |
| --- | --- | --- | --- | --- |
| **S1** | 3.254 | 381.02 | 0.0000 | H −> L (98.5%) |
| **S2** | 3.3101 | 374.56 | 0.0181 | H−1 −> L (96.8%) |
| **S3** | 3.7809 | 327.92 | 0.1138 | H−2 −> L (92.0%) |
| **S4** | 3.8385 | 323.00 | 0.0002 | H −> L+1 (98.7%) |
| **S5** | 3.8814 | 319.43 | 0.0257 | H−1 −> L+1 (94.7%) |
| **S6** | 4.0917 | 303.01 | 0.0702 | H−3 −> L (74.1%)  H−2 −> L+1 (21.2%) |
| **S7** | 4.1768 | 296.84 | 0.1040 | H −> L+2 (94.4%) |
| **S8** | 4.2069 | 294.72 | 0.0000 | H−4 −> L (97.6%) |
| **S9** | 4.2491 | 291.79 | 0.0332 | H−5 −> L (50.9%)  H−2 −> L+1 (29.3%)  H−3 −> L (11.5%) |
| **S10** | 4.3044 | 288.04 | 0.0384 | H−1 −> L+2 (75.8%)  H −> L+3 (14.8%)  H−5 −> L (5.4%) |
| **S11** | 4.3802 | 283.06 | 0.1815 | H−2 −> L+1 (44.6%)  H−5 −> L (31.5%)  H−3 −> L (9.1%)  H−3 −> L+1 (6.0%) |
| **S12** | 4.4392 | 279.29 | 0.0399 | H−1 −> L+3 (97.4%) |
| **S13** | 4.5599 | 271.9 | 0.0579 | H −> L+3 (74.1%)  H−1 −> L+2 (12.7%) |
| **S14** | 4.6138 | 268.72 | 0.0210 | H−6 −> L (60.8%)  H−2 −> L+2 (16.8%)  H−3 −> L+1 (12.7%) |
| **S15** | 4.6277 | 267.92 | 0.0199 | H−2 −> L+2 (79.9%)  H−6 −> L (12.8%) |
| **Cz-dfppy-CN** | **Energy (eV)** | **λ (nm)** | **Oscillator Strength (*f*)** | **Major contributions** |
| **S1** | 3.1676 | 391.41 | 0.0001 | H −> L (88.2%)  H−3 −> L (11.2%) |
| **S2** | 3.3577 | 369.25 | 0.0235 | H−1 −> L (97.0%) |
| **S3** | 3.7277 | 332.60 | 0.0022 | H −> L+1 (57.1%)  H−3 −> L (30.1%)  H−3 −> L+1 (5.6%) |
| **S4** | 3.7597 | 329.77 | 0.0058 | H−3 −> L (54.5%)  H −> L+1 (33.1%)  H −> L (6.7%) |
| **S5** | 3.821 | 324.48 | 0.1731 | H−4 −> L (88.5%) |
| **S6** | 3.8392 | 322.94 | 0.0575 | H −> L+2 (94.5%) |
| **S7** | 3.8726 | 320.16 | 0.0420 | H −> L+3 (93.2%) |
| **S8** | 3.9334 | 315.21 | 0.0322 | H−1 −> L+1 (94.6%) |
| **S9** | 3.9581 | 313.24 | 0.0003 | H−2 −> L (99.6%) |
| **S10** | 4.1312 | 300.12 | 0.0751 | H−5 −> L (70.9%)  H−4 −> L+1 (22.7%) |
| **S11** | 4.1554 | 298.37 | 0.0182 | H−1 −> L+2 (51.0%)  H −> L+4 (43.7%) |
| **S12** | 4.266 | 290.63 | 0.0000 | H−6 −> L (95.7%) |
| **S13** | 4.275 | 290.02 | 0.3519 | H −> L+4 (41.8%)  H−1 −> L+2 (34.7%)  H−4 −> L+1 (6.7%)  H−5 −> L (5.1%)  H−7 −> L (5.1%) |
| **S14** | 4.3033 | 288.11 | 0.0043 | H−3 −> L+1 (83.5%)  H −> L+1 (8.4%) |
| **S15** | 4.3326 | 286.17 | 0.0167 | H−7 −> L (64.8%)  H−4 −> L+1 (11.0%)  H −> L+4 (5.9%) |
| **tBuCz-dfppy-CN** | **Energy (eV)** | **λ (nm)** | **Oscillator Strength (*f*)** | **Major contributions** |
| **S1** | 3.0788 | 402.7 | 0.0002 | H −> L (90.1%)  H−3 −> L (9.1%) |
| **S2** | 3.3513 | 369.96 | 0.0239 | H−1 −> L (96.3%) |
| **S3** | 3.6319 | 341.38 | 0.0011 | H −> L+1 (60.2%)  H−3 −> L (27.4%) |
| **S4** | 3.6636 | 338.42 | 0.0015 | H−3 −> L (54.7%)  H −> L+1 (31.7%)  H −> L (6.2%) |
| **S5** | 3.7384 | 331.65 | 0.0462 | H −> L+2 (95.8%) |
| **S6** | 3.7802 | 327.98 | 0.0996 | H −> L+3 (92.4%) |
| **S7** | 3.814 | 325.08 | 0.1400 | H−4 −> L (88.4%) |
| **S8** | 3.8534 | 321.75 | 0.0001 | H−2 −> L (92.9%)  H−3 −> L (6.2%) |
| **S9** | 3.9262 | 315.79 | 0.0377 | H−1 −> L+1 (94.3%) |
| **S10** | 4.1011 | 302.32 | 0.1068 | H −> L+4 (76.5%)  H−1 −> L+2 (14.9%) |
| **S11** | 4.1281 | 300.34 | 0.0926 | H−5 −> L (68.5%)  H−4 −> L+1 (21.8%) |
| **S12** | 4.213 | 294.29 | 0.0036 | H−3 −> L+1 (83.1%)  H −> L+1 (7.1%)  H−2 −> L+1 (6.2%) |
| **S13** | 4.2399 | 292.42 | 0.2764 | H−1 −> L+2 (70.6%)  H −> L+4 (14.5%) |
| **S14** | 4.2572 | 291.23 | 0.0002 | H−6 −> L (95.9%) |
| **S15** | 4.3204 | 286.97 | 0.0044 | H−7 −> L (62.7%)  H−4 −> L+1 (15.8%)  H−5 −> L (6.5%) |
| **Pxz-dfppy-CN** | **Energy (eV)** | **λ (nm)** | **Oscillator Strength (*f*)** | **Excitation contributions** |
| **S1** | 2.8101 | 441.21 | 0.0001 | H -> L (99.9%) |
| **S2** | 3.2564 | 380.74 | 0.0002 | H -> L+2 (94.6%) |
| **S3** | 3.3511 | 369.98 | 0.0001 | H -> L+1 (96.0%) |
| **S4** | 3.3609 | 368.9 | 0.0232 | H-2 -> L (49.9%)  H-1 -> L (46.8%) |
| **S5** | 3.3654 | 368.41 | 0.0000 | H-1 -> L (50.8%)  H-2 -> L (47.6%) |
| **S6** | 3.6516 | 339.53 | 0.0013 | H -> L+3 (90.7%)  H -> L+8 (6.3%) |
| **S7** | 3.6739 | 337.47 | 0.0396 | H -> L+4 (94.2%) |
| **S8** | 3.8295 | 323.76 | 0.1440 | H-3 -> L (90.6%) |
| **S9** | 3.9366 | 314.95 | 0.0326 | H-2 -> L+1 (49.8%)  H-1 -> L+1 (45.1%) |
| **S10** | 3.9556 | 313.44 | 0.0008 | H-1 -> L+1 (51.8%)  H-2 -> L+1 (46.8%) |
| **S11** | 4.0974 | 302.59 | 0.1538 | H -> L+6 (57.5%)  H -> L+5 (31.4%)  H-4 -> L+4 (6.1%) |
| **S12** | 4.1194 | 300.98 | 0.0880 | H-1 -> L+2 (50.9%)  H-2 -> L+2 (43.2%) |
| **S13** | 4.1331 | 299.98 | 0.1117 | H-5 -> L (71.5%)  H-3 -> L+1 (20.7%) |
| **S14** | 4.1783 | 296.73 | 0.0463 | H-2 -> L+2 (46.6%)  H-1 -> L+2 (39.7%) |
| **S15** | 4.2426 | 292.24 | 0.0193 | H -> L+7 (92.0%) |


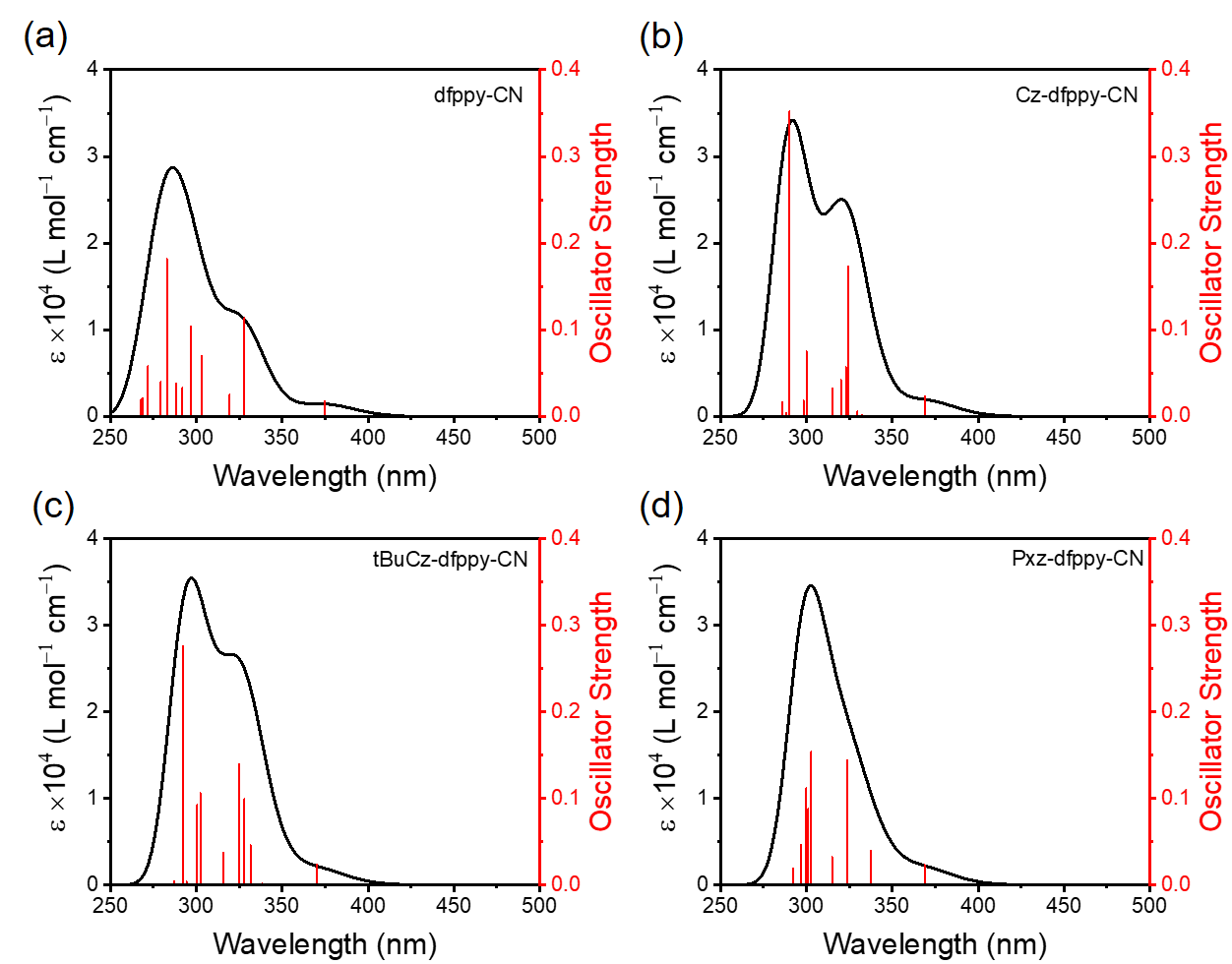


**Figure S10.** Simulated UV-vis absorption spectra of the studied Ir(III) complexes computed by TDDFT/CPCM using toluene as solvent. The red vertical lines refer to the unbroadened oscillator strengths of the singlet-singlet transitions, and the black line is the fitting line of UV-Vis absorption (FWHM 0.03 eV).


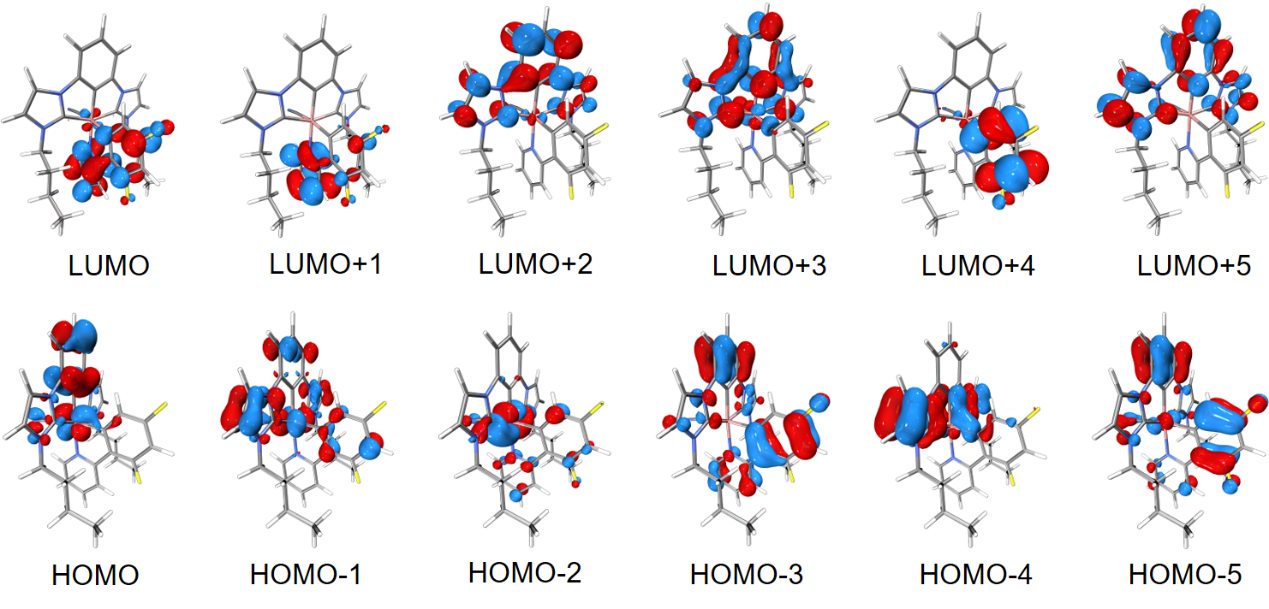


**Figure S11.** Selected molecular orbitals of **dfppy-CN** at optimized ground state geometry (isovalue = 0.04).


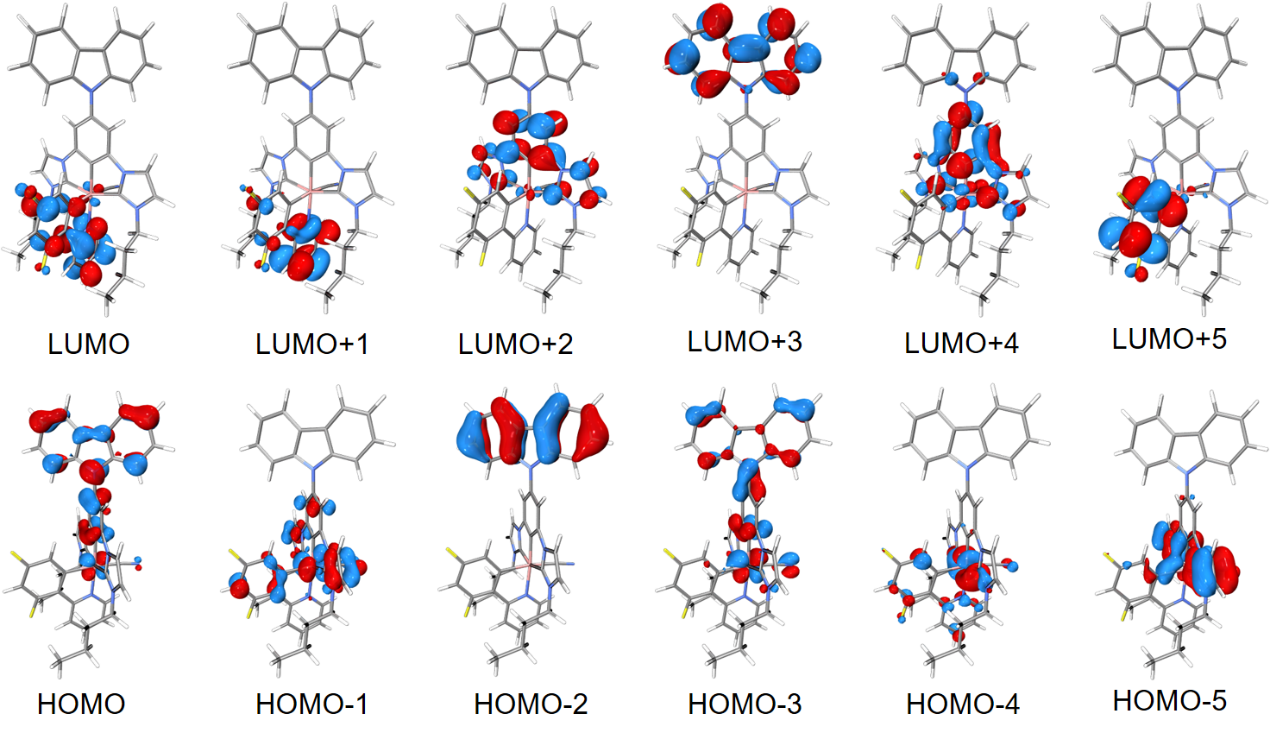


**Figure S12.** Selected molecular orbitals of **Cz-dfppy-CN** at optimized ground state geometry (isovalue = 0.04).


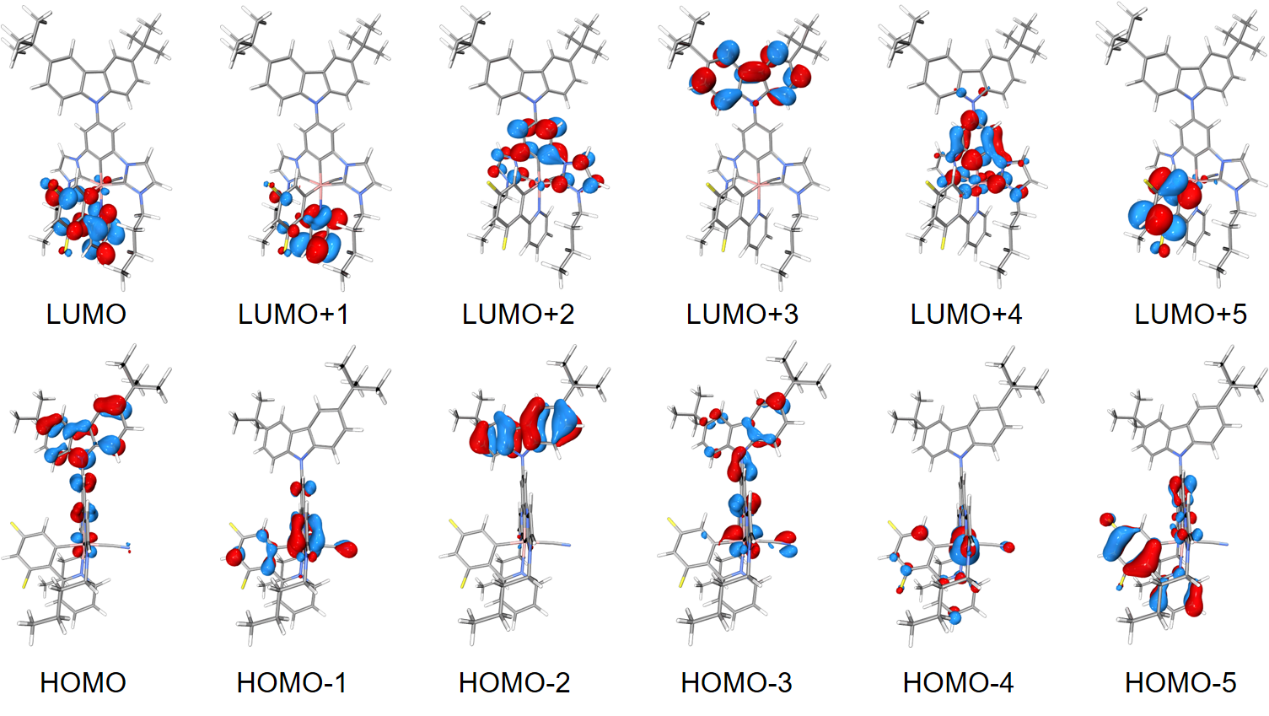


**Figure S13.** Selected molecular orbitals of **tBuCz-dfppy-CN** at optimized ground state geometry (isovalue = 0.04).


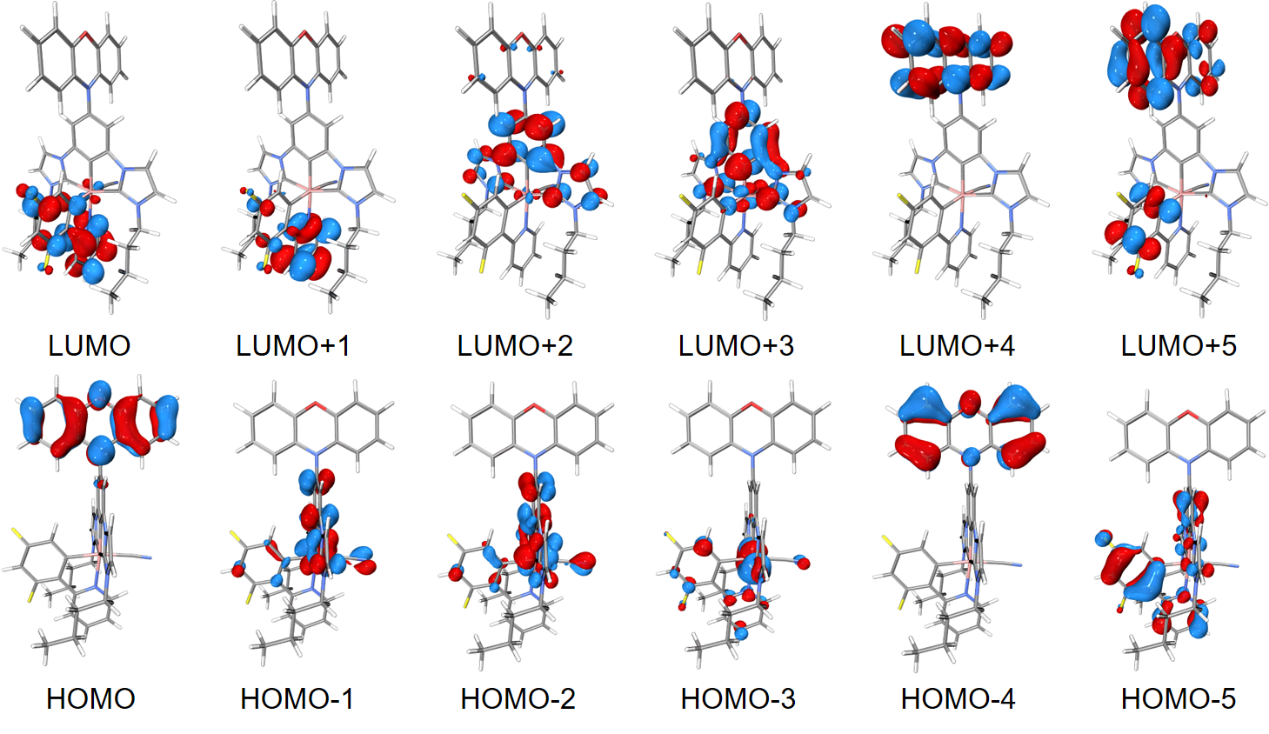


**Figure S14.** Selected molecular orbitals of **Pxz-dfppy-CN** at optimized ground state geometry (isovalue = 0.04).


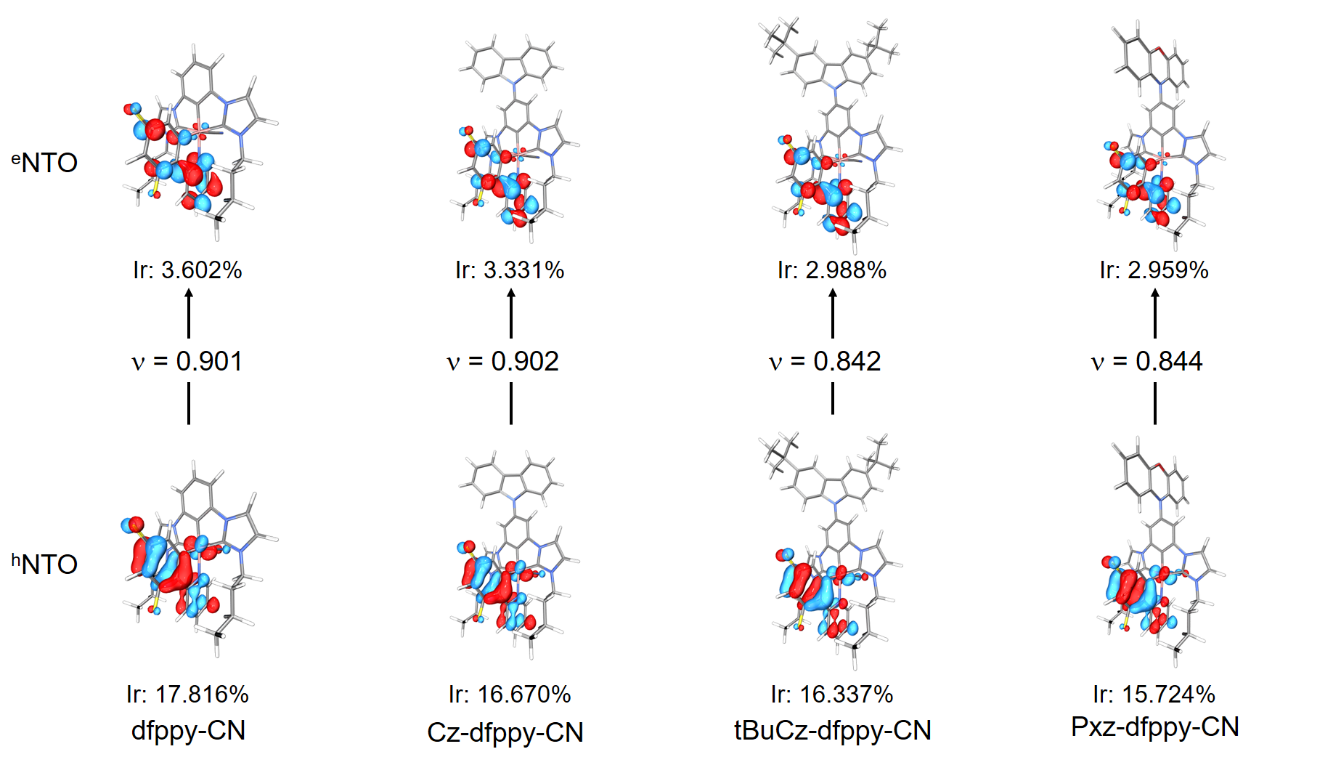


**Figure S15.** Natural transition orbital (NTO) pairs of S_0_→T_1_ transition of the studied Ir(III) complexes.

**Figure S16.** Correlation between the calculated aspect ratio and the emitting dipole orientation (EDO).


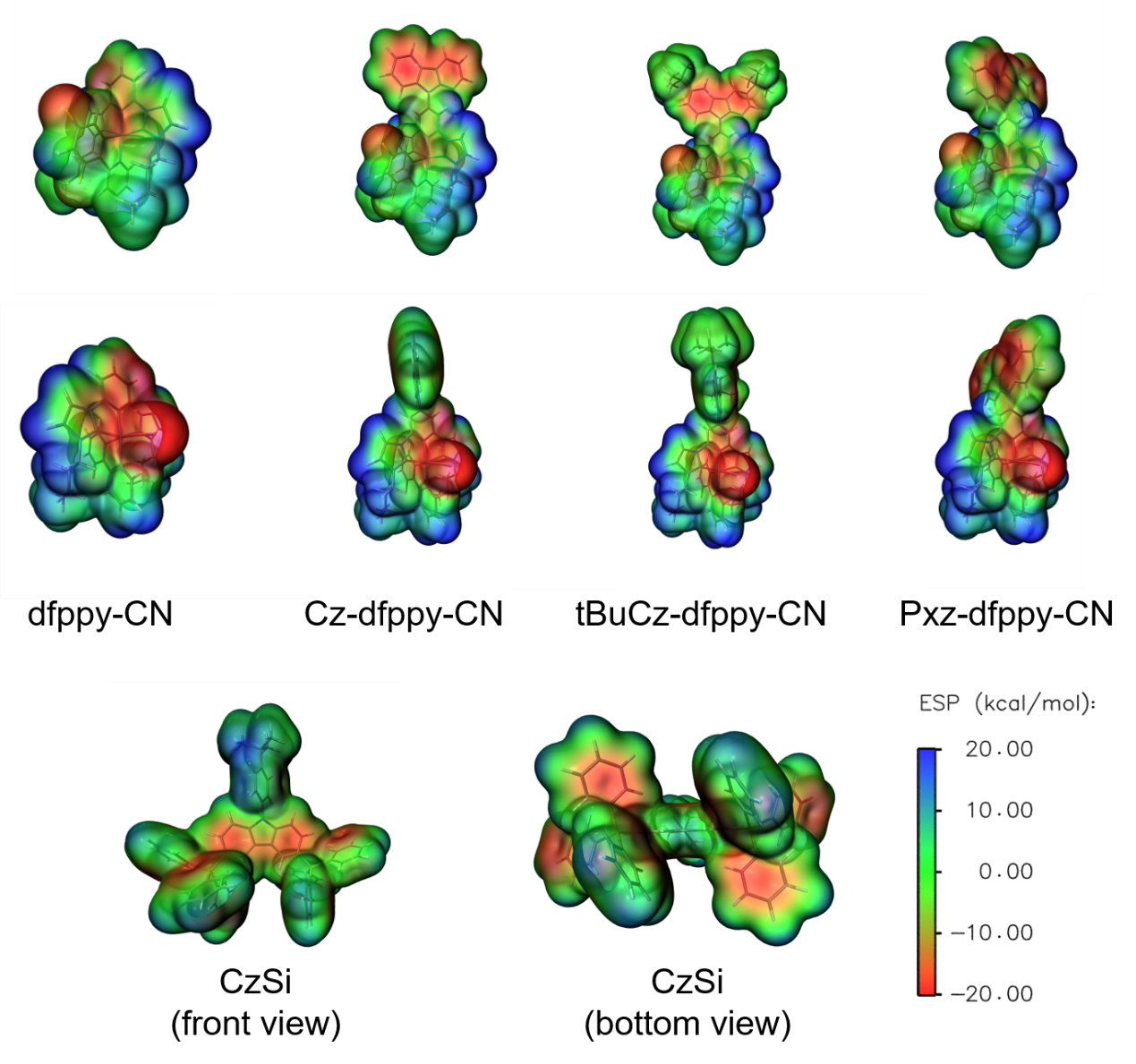


**Figure S17.** Maps of electrostatic potential (ESP) of the Ir(III) complexes and the host material CzSi.

**Table S4.** Cartesian coordinates of the optimized ground-state (S0) and excited state (T1) geometry of **dfppy-CN**.

| Ground state of **dfppy-CN** (S_0_) | Excited state of **dfppy-CN** (T_1_) |
| --- | --- |

| Ir | −0.75379 | −5.2E−06 | −0.63938 | Ir | 0.810572 | −3.3E−06 | −0.6793 |
| --- | --- | --- | --- | --- | --- | --- | --- |
| N | 1.334158 | −1.4E−05 | −1.24529 | N | −1.28206 | 3.28E−05 | −1.31517 |
| F | −0.22649 | 4.54E−05 | 4.802182 | F | 0.276977 | −1.7E−05 | 4.75797 |
| F | 3.727715 | 1.12E−05 | 2.295798 | F | −3.72127 | 2.85E−05 | 2.268615 |
| N | −2.40853 | 2.303093 | 0.038756 | N | 2.399457 | −2.31833 | 0.049012 |
| N | −0.6037 | 3.230922 | −0.69493 | N | 0.565012 | −3.22219 | −0.64266 |
| N | −0.60371 | −3.23093 | −0.69489 | N | 0.565128 | 3.222191 | −0.64263 |
| N | −2.40853 | −2.30309 | 0.038797 | N | 2.399539 | 2.31826 | 0.049042 |
| C | 0.210391 | 9.2E−06 | 1.182078 | C | −0.22163 | 2.1E−06 | 1.139431 |
| N | −1.67896 | −3.5E−05 | −3.7305 | N | 1.647969 | 2.59E−05 | −3.78264 |
| C | −1.15747 | 2.017461 | −0.47338 | C | 1.151632 | −2.02838 | −0.48079 |
| C | −3.23854 | 1.200871 | 0.3707 | C | 3.227989 | −1.22424 | 0.370797 |
| C | 1.752932 | −2.7E−05 | −2.52444 | C | −1.73135 | 5.13E−05 | −2.60001 |
| H | 0.964717 | −3.3E−05 | −3.26865 | H | −0.95957 | 5.34E−05 | −3.36008 |
| C | −2.59263 | 4.4E−06 | 0.091863 | C | 2.589541 | −4E−05 | 0.07468 |
| C | 2.240261 | −4.5E−06 | −0.22463 | C | −2.22978 | 2.91E−05 | −0.26656 |
| C | −1.15748 | −2.01747 | −0.47335 | C | 1.151704 | 2.028357 | −0.48076 |
| C | 1.638241 | 6.8E−06 | 1.112607 | C | −1.65234 | 1.42E−05 | 1.04177 |
| C | 0.776983 | −3.424 | −1.13216 | C | −0.82911 | 3.388485 | −1.07039 |
| H | 1.019995 | −2.59635 | −1.79952 | H | −1.06842 | 2.531029 | −1.69961 |
| H | 0.82052 | −4.34971 | −1.71347 | H | −0.8857 | 4.296122 | −1.67811 |
| C | 0.776999 | 3.423982 | −1.1322 | C | −0.82923 | −3.38844 | −1.07042 |
| H | 0.820542 | 4.349684 | −1.71351 | H | −0.88586 | −4.29605 | −1.67817 |
| H | 1.020014 | 2.596317 | −1.79954 | H | −1.06852 | −2.53095 | −1.69961 |
| C | −1.40693 | −2.4E−05 | −2.59088 | C | 1.326121 | 9.3E−06 | −2.65654 |
| C | −1.47208 | 4.255914 | −0.32282 | C | 1.404948 | −4.25608 | −0.21836 |
| H | −1.20461 | 5.294959 | −0.42654 | H | 1.104423 | −5.28971 | −0.27221 |
| C | −2.6105 | 3.671159 | 0.133037 | C | 2.560331 | −3.68969 | 0.212639 |
| H | −3.5285 | 4.101733 | 0.496713 | H | 3.461434 | −4.13099 | 0.604446 |
| C | 3.097233 | −3.1E−05 | −2.86845 | C | −3.05967 | 6.44E−05 | −2.9456 |
| H | 3.387036 | −4.3E−05 | −3.91275 | H | −3.34187 | 8.08E−05 | −3.99222 |
| C | −3.23854 | −1.20086 | 0.370722 | C | 3.228033 | 1.22413 | 0.370812 |
| C | −4.52007 | 1.226642 | 0.919076 | C | 4.496995 | −1.24029 | 0.929586 |
| H | −5.03005 | 2.158868 | 1.14001 | H | 5.014948 | −2.16308 | 1.16561 |
| C | −0.39758 | 2.31E−05 | 2.444808 | C | 0.409585 | −7.4E−06 | 2.382574 |
| H | −1.47753 | 2.64E−05 | 2.54112 | H | 1.489401 | −1.7E−05 | 2.473197 |
| C | −1.47209 | −4.25592 | −0.32277 | C | 1.405102 | 4.256042 | −0.21832 |
| H | −1.20464 | −5.29496 | −0.42647 | H | 1.104615 | 5.289685 | −0.27216 |
| C | −4.52007 | −1.22662 | 0.919098 | C | 4.49704 | 1.240133 | 0.929602 |
| H | −5.03006 | −2.15884 | 1.140048 | H | 5.015025 | 2.162899 | 1.165638 |
| C | −2.61051 | −3.67115 | 0.13309 | C | 2.560463 | 3.68961 | 0.212682 |
| H | −3.52852 | −4.10172 | 0.496772 | H | 3.461581 | 4.130873 | 0.604496 |
| C | 1.742196 | 3.462329 | 0.053408 | C | −1.77317 | −3.4465 | 0.130784 |
| H | 1.584623 | 2.55835 | 0.649393 | H | −1.60474 | −2.55062 | 0.735342 |
| H | 1.491006 | 4.312491 | 0.700495 | H | −1.52133 | −4.31288 | 0.756803 |
| C | 3.614794 | −6.5E−06 | −0.51992 | C | −3.60744 | 0.000041 | −0.59244 |
| H | 4.332266 | 1.4E−06 | 0.284517 | H | −4.32512 | 3.74E−05 | 0.214864 |
| C | 0.378417 | 3.21E−05 | 3.593809 | C | −0.35279 | −5.4E−06 | 3.550492 |
| C | −5.14635 | 1.61E−05 | 1.185298 | C | 5.110222 | −9.2E−05 | 1.195604 |
| C | 1.742188 | −3.46233 | 0.053439 | C | −1.77305 | 3.446538 | 0.130808 |
| H | 1.584619 | −2.55835 | 0.649413 | H | −1.60465 | 2.550644 | 0.735344 |
| H | 1.491005 | −4.31249 | 0.700537 | H | −1.5212 | 4.312899 | 0.756851 |
| C | 4.042398 | −1.9E−05 | −1.84134 | C | −4.0382 | 5.86E−05 | −1.90061 |
| H | 5.104355 | −2E−05 | −2.06499 | H | −5.0987 | 6.74E−05 | −2.12991 |
| C | 1.770283 | 2.79E−05 | 3.562985 | C | −1.74461 | 6.9E−06 | 3.514712 |
| H | 2.365513 | 0.000034 | 4.466619 | H | −2.34016 | 9.3E−06 | 4.41926 |
| C | 2.363112 | 1.53E−05 | 2.313403 | C | −2.35055 | 1.61E−05 | 2.273973 |
| C | 3.206524 | −3.54722 | −0.38324 | C | −3.24246 | 3.507572 | −0.29413 |
| H | 3.409294 | −2.7567 | −1.11538 | H | −3.44085 | 2.702656 | −1.01033 |
| H | 3.38414 | −4.5 | −0.89907 | H | −3.43826 | 4.454118 | −0.81586 |
| C | 3.206534 | 3.54721 | −0.38326 | C | −3.24259 | −3.5075 | −0.29414 |
| H | 3.384153 | 4.499984 | −0.89911 | H | −3.43841 | −4.45403 | −0.8159 |
| H | 3.409311 | 2.756683 | −1.11538 | H | −3.44096 | −2.70256 | −1.01033 |
| C | 4.171845 | −3.40472 | 0.796044 | C | −4.19094 | 3.358867 | 0.897815 |
| H | 3.998963 | −4.1858 | 1.544736 | H | −4.01336 | 4.137706 | 1.648526 |
| H | 4.045834 | −2.43698 | 1.292137 | H | −4.0544 | 2.387441 | 1.382416 |
| H | 5.213819 | −3.48031 | 0.469248 | H | −5.23664 | 3.431134 | 0.582163 |
| C | 4.171844 | 3.404733 | 0.796036 | C | −4.19105 | −3.35879 | 0.897808 |
| H | 4.045838 | 2.436995 | 1.29213 | H | −4.05449 | −2.38737 | 1.382422 |
| H | 3.998939 | 4.185806 | 1.544725 | H | −4.01348 | −4.13764 | 1.648507 |
| H | 5.21382 | 3.480336 | 0.469255 | H | −5.23676 | −3.43104 | 0.582163 |
| H | −6.14431 | 2.08E−05 | 1.610985 | H | 6.102778 | −0.00011 | 1.633643 |

**Table S5.** Cartesian coordinates of the optimized ground-state (S0) and excited state (T1) geometry of **Cz-dfppy-CN.**

| Ground state of **tBuCz-dfppy-CN** (S_0_) | Excited state of **tBuCz-dfppy-CN** (T_1_) |
| --- | --- |

| Ir | 1.031494 | 0.37881 | −0.82207 | Ir | −0.99323 | −0.34499 | −0.89429 |
| --- | --- | --- | --- | --- | --- | --- | --- |
| N | 3.201858 | 0.436345 | −0.90288 | N | −3.16602 | −0.39932 | −0.98226 |
| F | 0.260645 | −1.87104 | 4.100273 | F | −0.16529 | 1.573581 | 4.169761 |
| F | 4.693932 | −1.205 | 2.75104 | F | −4.64922 | 1.031467 | 2.831366 |
| N | −0.75595 | 2.351591 | 0.367863 | N | 0.747813 | −2.38307 | 0.221377 |
| N | 1.163092 | 3.328945 | 0.497543 | N | −1.1931 | −3.32273 | 0.334254 |
| N | 1.218778 | −2.55988 | −2.16042 | N | −1.24616 | 2.700815 | −1.93634 |
| N | −0.71564 | −1.84788 | −1.52314 | N | 0.708143 | 1.953648 | −1.40201 |
| C | 1.538781 | −0.44224 | 0.999646 | C | −1.52534 | 0.363672 | 0.998215 |
| N | 0.844484 | 1.711983 | −3.75543 | N | −0.8673 | −1.49906 | −3.90407 |
| N | −5.05576 | −0.08497 | 0.078961 | N | 5.010515 | 0.071601 | 0.075941 |
| C | 0.584209 | 2.185517 | 0.069608 | C | −0.59662 | −2.19417 | −0.0761 |
| C | −1.62773 | 1.28778 | 0.028432 | C | 1.618147 | −1.31681 | −0.06706 |
| C | 3.91005 | 0.913754 | −1.94326 | C | −3.91445 | −0.80688 | −2.04119 |
| H | 3.319958 | 1.282348 | −2.77451 | H | −3.34911 | −1.12889 | −2.90798 |
| C | −0.92409 | 0.248323 | −0.5737 | C | 0.908804 | −0.22359 | −0.6171 |
| C | 3.841965 | −0.0543 | 0.1983 | C | −3.82302 | 0.038495 | 0.190039 |
| C | 0.619046 | −1.49166 | −1.59039 | C | −0.62976 | 1.59156 | −1.50315 |
| C | 2.942355 | −0.54063 | 1.249496 | C | −2.93972 | 0.444552 | 1.241366 |
| C | 2.665532 | −2.68387 | −2.32692 | C | −2.70311 | 2.816553 | −2.06903 |
| H | 3.051853 | −1.68298 | −2.52271 | H | −3.07835 | 1.816259 | −2.2856 |
| H | 2.851527 | −3.29643 | −3.21392 | H | −2.91019 | 3.462306 | −2.92744 |
| C | 2.606844 | 3.556662 | 0.495265 | C | −2.64752 | −3.51823 | 0.325173 |
| H | 2.77858 | 4.629714 | 0.370299 | H | −2.84107 | −4.57277 | 0.107758 |
| H | 3.009603 | 3.047368 | −0.38095 | H | −3.03969 | −2.91464 | −0.49355 |
| C | 0.851434 | 1.224829 | −2.69013 | C | −0.9174 | −1.07813 | −2.81251 |
| C | 0.222276 | 4.189847 | 1.061548 | C | −0.26677 | −4.20926 | 0.891217 |
| H | 0.497869 | 5.153039 | 1.459214 | H | −0.56323 | −5.17097 | 1.277123 |
| C | −0.9867 | 3.577157 | 0.974173 | C | 0.954322 | −3.62304 | 0.816915 |
| H | −1.96825 | 3.905014 | 1.273453 | H | 1.926476 | −3.97348 | 1.12098 |
| C | 5.297477 | 0.933647 | −1.95334 | C | −5.28781 | −0.82191 | −2.04567 |
| H | 5.825254 | 1.327851 | −2.81387 | H | −5.81955 | −1.16094 | −2.92736 |
| C | −1.60638 | −0.90319 | −0.95644 | C | 1.593791 | 0.979799 | −0.90503 |
| C | −2.99652 | 1.198953 | 0.266208 | C | 2.976729 | −1.24128 | 0.166896 |
| H | −3.55922 | 1.982944 | 0.760116 | H | 3.536587 | −2.04537 | 0.627167 |
| C | 0.650325 | −0.89967 | 1.981659 | C | −0.61616 | 0.741242 | 1.984601 |
| H | −0.42199 | −0.84287 | 1.831879 | H | 0.454232 | 0.686086 | 1.818987 |
| C | 0.296313 | −3.56878 | −2.43525 | C | −0.33874 | 3.751633 | −2.09872 |
| H | 0.589914 | −4.49985 | −2.89223 | H | −0.65163 | 4.72356 | −2.44413 |
| C | −2.97946 | −1.03337 | −0.76612 | C | 2.951891 | 1.095792 | −0.68793 |
| H | −3.53547 | −1.91001 | −1.07822 | H | 3.51256 | 1.984343 | −0.94748 |
| C | −0.92235 | −3.11807 | −2.03976 | C | 0.890648 | 3.283455 | −1.76748 |
| H | −1.89584 | −3.5766 | −2.09081 | H | 1.853907 | 3.765774 | −1.77026 |
| C | 3.259832 | 3.029108 | 1.77353 | C | −3.27162 | −3.08494 | 1.65164 |
| H | 2.972898 | 1.979976 | 1.893363 | H | −2.96791 | −2.05209 | 1.844605 |
| H | 2.85292 | 3.566515 | 2.639594 | H | −2.86898 | −3.69973 | 2.467809 |
| C | 5.247123 | −0.05635 | 0.23779 | C | −5.23888 | 0.03502 | 0.214631 |
| H | 5.754747 | −0.44292 | 1.106544 | H | −5.73732 | 0.370539 | 1.112253 |
| C | 1.133533 | −1.43458 | 3.166359 | C | −1.0702 | 1.205448 | 3.219109 |
| C | −5.89059 | 1.905422 | −1.22881 | C | 5.812597 | −2.12372 | −0.91378 |
| H | −4.91431 | 2.221965 | −1.57872 | H | 4.842775 | −2.46214 | −1.25702 |
| C | −3.65837 | 0.029646 | −0.14621 | C | 3.63713 | −0.02668 | −0.1527 |
| C | −6.03403 | 0.783961 | −0.40915 | C | 5.972344 | −0.90754 | −0.24826 |
| C | −7.30669 | 0.342617 | 0.041516 | C | 7.249325 | −0.43184 | 0.137046 |
| C | 3.324507 | −3.28271 | −1.08361 | C | −3.33353 | 3.354086 | −0.78436 |
| H | 3.02242 | −2.68562 | −0.21783 | H | −3.01617 | 2.709696 | 0.040455 |
| H | 2.936421 | −4.29569 | −0.91698 | H | −2.94806 | 4.361362 | −0.57681 |
| C | −8.31615 | 2.18 | −1.13972 | C | 8.22587 | −2.44371 | −0.74227 |
| C | −8.45228 | 1.054946 | −0.33038 | C | 8.380947 | −1.21104 | −0.10892 |
| H | −9.43289 | 0.73105 | 0.006024 | H | 9.365447 | −0.85666 | 0.179649 |
| C | 5.973913 | 0.437555 | −0.83777 | C | −5.97772 | −0.383 | −0.87034 |
| H | 7.058585 | 0.434189 | −0.80338 | H | −7.06213 | −0.37804 | −0.8303 |
| C | −7.04806 | 2.595126 | −1.58458 | C | 6.957124 | −2.88704 | −1.14573 |
| H | −6.96638 | 3.470388 | −2.22197 | H | 6.860065 | −3.8389 | −1.65765 |
| C | 2.493305 | −1.54517 | 3.442976 | C | −2.42849 | 1.304104 | 3.506036 |
| H | 2.858945 | −1.96412 | 4.371218 | H | −2.78747 | 1.66322 | 4.462675 |
| C | −5.67793 | −1.07845 | 0.837602 | C | 5.671758 | 1.167161 | 0.668724 |
| C | −7.07954 | −0.84694 | 0.83705 | C | 7.058209 | 0.883255 | 0.722299 |
| C | 3.363962 | −1.09118 | 2.468795 | C | −3.31739 | 0.924329 | 2.518853 |
| C | 4.851335 | −3.31329 | −1.18482 | C | −4.86242 | 3.374846 | −0.85529 |
| H | 5.21272 | −2.31655 | −1.46426 | H | −5.21799 | 2.389873 | −1.177 |
| H | 5.155197 | −3.9908 | −1.99348 | H | −5.18925 | 4.096575 | −1.61618 |
| C | −7.91943 | −1.7146 | 1.543844 | C | 7.931719 | 1.806213 | 1.299696 |
| H | −8.9933 | −1.55214 | 1.552752 | H | 8.996191 | 1.59948 | 1.348405 |
| C | 4.785305 | 3.150038 | 1.747827 | C | −4.79971 | −3.17062 | 1.626182 |
| H | 5.072118 | 4.209535 | 1.726027 | H | −5.11121 | −4.21997 | 1.533896 |
| H | 5.165278 | 2.707556 | 0.8194 | H | −5.17129 | −2.64878 | 0.737518 |
| C | −5.10782 | −2.14324 | 1.538602 | C | 5.140011 | 2.332693 | 1.222568 |
| H | −4.03488 | −2.30007 | 1.542338 | H | 4.074157 | 2.52433 | 1.232458 |
| C | −7.35857 | −2.78421 | 2.237403 | C | 7.412199 | 2.989604 | 1.823509 |
| C | 5.509923 | −3.74065 | 0.129107 | C | −5.49343 | 3.714534 | 0.497021 |
| H | 5.173957 | −4.73813 | 0.433426 | H | −5.15232 | 4.690122 | 0.8627 |
| H | 5.260199 | −3.04618 | 0.937724 | H | −5.22831 | 2.963046 | 1.246763 |
| H | 6.600249 | −3.76935 | 0.037078 | H | −6.58546 | 3.747053 | 0.427135 |
| C | −5.96742 | −2.99063 | 2.23515 | C | 6.03226 | 3.241935 | 1.79141 |
| H | −5.55114 | −3.82651 | 2.789337 | H | 5.644963 | 4.15791 | 2.225567 |
| C | 5.44093 | 2.456665 | 2.944603 | C | −5.42937 | −2.54306 | 2.872049 |
| H | 5.208856 | 1.386812 | 2.954433 | H | −5.18183 | −1.47919 | 2.936329 |
| H | 5.086211 | 2.881249 | 3.890298 | H | −5.07112 | −3.02815 | 3.787565 |
| H | 6.530046 | 2.562855 | 2.918731 | H | −6.52031 | −2.63219 | 2.85156 |
| H | −9.19666 | 2.742018 | −1.43484 | H | 9.096046 | −3.06145 | −0.93847 |
| H | −7.99933 | −3.46513 | 2.788798 | H | 8.078961 | 3.717415 | 2.274067 |

**Table S6.** Cartesian coordinates of the optimized ground-state (S0) and excited state (T1) geometry of **tBuCz−dfppyCN**.

| Ground state of **tBuCz-dfppy-CN** (S_0_) | Excited state of **tBuCz-dfppy-CN** (T_1_) |
| --- | --- |

| \| Ir \| 1.031494 \| 0.37881 \| −0.82207 \| Ir \| −0.99323 \| −0.34499 \| −0.89429 \| \| --- \| --- \| --- \| --- \| --- \| --- \| --- \| --- \| \| N \| 3.201858 \| 0.436345 \| −0.90288 \| N \| −3.16602 \| −0.39932 \| −0.98226 \| \| F \| 0.260645 \| −1.87104 \| 4.100273 \| F \| −0.16529 \| 1.573581 \| 4.169761 \| \| F \| 4.693932 \| −1.205 \| 2.75104 \| F \| −4.64922 \| 1.031467 \| 2.831366 \| \| N \| −0.75595 \| 2.351591 \| 0.367863 \| N \| 0.747813 \| −2.38307 \| 0.221377 \| \| N \| 1.163092 \| 3.328945 \| 0.497543 \| N \| −1.1931 \| −3.32273 \| 0.334254 \| \| N \| 1.218778 \| −2.55988 \| −2.16042 \| N \| −1.24616 \| 2.700815 \| −1.93634 \| \| N \| −0.71564 \| −1.84788 \| −1.52314 \| N \| 0.708143 \| 1.953648 \| −1.40201 \| \| C \| 1.538781 \| −0.44224 \| 0.999646 \| C \| −1.52534 \| 0.363672 \| 0.998215 \| \| N \| 0.844484 \| 1.711983 \| −3.75543 \| N \| −0.8673 \| −1.49906 \| −3.90407 \| \| N \| −5.05576 \| −0.08497 \| 0.078961 \| N \| 5.010515 \| 0.071601 \| 0.075941 \| \| C \| 0.584209 \| 2.185517 \| 0.069608 \| C \| −0.59662 \| −2.19417 \| −0.0761 \| \| C \| −1.62773 \| 1.28778 \| 0.028432 \| C \| 1.618147 \| −1.31681 \| −0.06706 \| \| C \| 3.91005 \| 0.913754 \| −1.94326 \| C \| −3.91445 \| −0.80688 \| −2.04119 \| \| H \| 3.319958 \| 1.282348 \| −2.77451 \| H \| −3.34911 \| −1.12889 \| −2.90798 \| \| C \| −0.92409 \| 0.248323 \| −0.5737 \| C \| 0.908804 \| −0.22359 \| −0.6171 \| \| C \| 3.841965 \| −0.0543 \| 0.1983 \| C \| −3.82302 \| 0.038495 \| 0.190039 \| \| C \| 0.619046 \| −1.49166 \| −1.59039 \| C \| −0.62976 \| 1.59156 \| −1.50315 \| \| C \| 2.942355 \| −0.54063 \| 1.249496 \| C \| −2.93972 \| 0.444552 \| 1.241366 \| \| C \| 2.665532 \| −2.68387 \| −2.32692 \| C \| −2.70311 \| 2.816553 \| −2.06903 \| \| H \| 3.051853 \| −1.68298 \| −2.52271 \| H \| −3.07835 \| 1.816259 \| −2.2856 \| \| H \| 2.851527 \| −3.29643 \| −3.21392 \| H \| −2.91019 \| 3.462306 \| −2.92744 \| \| C \| 2.606844 \| 3.556662 \| 0.495265 \| C \| −2.64752 \| −3.51823 \| 0.325173 \| \| H \| 2.77858 \| 4.629714 \| 0.370299 \| H \| −2.84107 \| −4.57277 \| 0.107758 \| \| H \| 3.009603 \| 3.047368 \| −0.38095 \| H \| −3.03969 \| −2.91464 \| −0.49355 \| \| C \| 0.851434 \| 1.224829 \| −2.69013 \| C \| −0.9174 \| −1.07813 \| −2.81251 \| \| C \| 0.222276 \| 4.189847 \| 1.061548 \| C \| −0.26677 \| −4.20926 \| 0.891217 \| \| H \| 0.497869 \| 5.153039 \| 1.459214 \| H \| −0.56323 \| −5.17097 \| 1.277123 \| \| C \| −0.9867 \| 3.577157 \| 0.974173 \| C \| 0.954322 \| −3.62304 \| 0.816915 \| \| H \| −1.96825 \| 3.905014 \| 1.273453 \| H \| 1.926476 \| −3.97348 \| 1.12098 \| \| C \| 5.297477 \| 0.933647 \| −1.95334 \| C \| −5.28781 \| −0.82191 \| −2.04567 \| \| H \| 5.825254 \| 1.327851 \| −2.81387 \| H \| −5.81955 \| −1.16094 \| −2.92736 \| \| C \| −1.60638 \| −0.90319 \| −0.95644 \| C \| 1.593791 \| 0.979799 \| −0.90503 \| \| C \| −2.99652 \| 1.198953 \| 0.266208 \| C \| 2.976729 \| −1.24128 \| 0.166896 \| \| H \| −3.55922 \| 1.982944 \| 0.760116 \| H \| 3.536587 \| −2.04537 \| 0.627167 \| \| C \| 0.650325 \| −0.89967 \| 1.981659 \| C \| −0.61616 \| 0.741242 \| 1.984601 \| \| H \| −0.42199 \| −0.84287 \| 1.831879 \| H \| 0.454232 \| 0.686086 \| 1.818987 \| \| C \| 0.296313 \| −3.56878 \| −2.43525 \| C \| −0.33874 \| 3.751633 \| −2.09872 \| \| H \| 0.589914 \| −4.49985 \| −2.89223 \| H \| −0.65163 \| 4.72356 \| −2.44413 \| \| C \| −2.97946 \| −1.03337 \| −0.76612 \| C \| 2.951891 \| 1.095792 \| −0.68793 \| \| H \| −3.53547 \| −1.91001 \| −1.07822 \| H \| 3.51256 \| 1.984343 \| −0.94748 \| \| C \| −0.92235 \| −3.11807 \| −2.03976 \| C \| 0.890648 \| 3.283455 \| −1.76748 \| \| H \| −1.89584 \| −3.5766 \| −2.09081 \| H \| 1.853907 \| 3.765774 \| −1.77026 \| \| C \| 3.259832 \| 3.029108 \| 1.77353 \| C \| −3.27162 \| −3.08494 \| 1.65164 \| \| H \| 2.972898 \| 1.979976 \| 1.893363 \| H \| −2.96791 \| −2.05209 \| 1.844605 \| \| H \| 2.85292 \| 3.566515 \| 2.639594 \| H \| −2.86898 \| −3.69973 \| 2.467809 \| \| C \| 5.247123 \| −0.05635 \| 0.23779 \| C \| −5.23888 \| 0.03502 \| 0.214631 \| \| H \| 5.754747 \| −0.44292 \| 1.106544 \| H \| −5.73732 \| 0.370539 \| 1.112253 \| \| C \| 1.133533 \| −1.43458 \| 3.166359 \| C \| −1.0702 \| 1.205448 \| 3.219109 \| \| C \| −5.89059 \| 1.905422 \| −1.22881 \| C \| 5.812597 \| −2.12372 \| −0.91378 \| \| H \| −4.91431 \| 2.221965 \| −1.57872 \| H \| 4.842775 \| −2.46214 \| −1.25702 \| \| C \| −3.65837 \| 0.029646 \| −0.14621 \| C \| 3.63713 \| −0.02668 \| −0.1527 \| \| C \| −6.03403 \| 0.783961 \| −0.40915 \| C \| 5.972344 \| −0.90754 \| −0.24826 \| \| C \| −7.30669 \| 0.342617 \| 0.041516 \| C \| 7.249325 \| −0.43184 \| 0.137046 \| \| C \| 3.324507 \| −3.28271 \| −1.08361 \| C \| −3.33353 \| 3.354086 \| −0.78436 \| \| H \| 3.02242 \| −2.68562 \| −0.21783 \| H \| −3.01617 \| 2.709696 \| 0.040455 \| \| H \| 2.936421 \| −4.29569 \| −0.91698 \| H \| −2.94806 \| 4.361362 \| −0.57681 \| \| C \| −8.31615 \| 2.18 \| −1.13972 \| C \| 8.22587 \| −2.44371 \| −0.74227 \| \| C \| −8.45228 \| 1.054946 \| −0.33038 \| C \| 8.380947 \| −1.21104 \| −0.10892 \| \| H \| −9.43289 \| 0.73105 \| 0.006024 \| H \| 9.365447 \| −0.85666 \| 0.179649 \| \| C \| 5.973913 \| 0.437555 \| −0.83777 \| C \| −5.97772 \| −0.383 \| −0.87034 \| \| H \| 7.058585 \| 0.434189 \| −0.80338 \| H \| −7.06213 \| −0.37804 \| −0.8303 \| \| C \| −7.04806 \| 2.595126 \| −1.58458 \| C \| 6.957124 \| −2.88704 \| −1.14573 \| \| H \| −6.96638 \| 3.470388 \| −2.22197 \| H \| 6.860065 \| −3.8389 \| −1.65765 \| \| C \| 2.493305 \| −1.54517 \| 3.442976 \| C \| −2.42849 \| 1.304104 \| 3.506036 \| \| H \| 2.858945 \| −1.96412 \| 4.371218 \| H \| −2.78747 \| 1.66322 \| 4.462675 \| \| C \| −5.67793 \| −1.07845 \| 0.837602 \| C \| 5.671758 \| 1.167161 \| 0.668724 \| \| C \| −7.07954 \| −0.84694 \| 0.83705 \| C \| 7.058209 \| 0.883255 \| 0.722299 \| \| C \| 3.363962 \| −1.09118 \| 2.468795 \| C \| −3.31739 \| 0.924329 \| 2.518853 \| \| C \| 4.851335 \| −3.31329 \| −1.18482 \| C \| −4.86242 \| 3.374846 \| −0.85529 \| \| H \| 5.21272 \| −2.31655 \| −1.46426 \| H \| −5.21799 \| 2.389873 \| −1.177 \| \| H \| 5.155197 \| −3.9908 \| −1.99348 \| H \| −5.18925 \| 4.096575 \| −1.61618 \| \| C \| −7.91943 \| −1.7146 \| 1.543844 \| C \| 7.931719 \| 1.806213 \| 1.299696 \| \| H \| −8.9933 \| −1.55214 \| 1.552752 \| H \| 8.996191 \| 1.59948 \| 1.348405 \| \| C \| 4.785305 \| 3.150038 \| 1.747827 \| C \| −4.79971 \| −3.17062 \| 1.626182 \| \| H \| 5.072118 \| 4.209535 \| 1.726027 \| H \| −5.11121 \| −4.21997 \| 1.533896 \| \| H \| 5.165278 \| 2.707556 \| 0.8194 \| H \| −5.17129 \| −2.64878 \| 0.737518 \| \| C \| −5.10782 \| −2.14324 \| 1.538602 \| C \| 5.140011 \| 2.332693 \| 1.222568 \| \| H \| −4.03488 \| −2.30007 \| 1.542338 \| H \| 4.074157 \| 2.52433 \| 1.232458 \| \| C \| −7.35857 \| −2.78421 \| 2.237403 \| C \| 7.412199 \| 2.989604 \| 1.823509 \| \| C \| 5.509923 \| −3.74065 \| 0.129107 \| C \| −5.49343 \| 3.714534 \| 0.497021 \| \| H \| 5.173957 \| −4.73813 \| 0.433426 \| H \| −5.15232 \| 4.690122 \| 0.8627 \| \| H \| 5.260199 \| −3.04618 \| 0.937724 \| H \| −5.22831 \| 2.963046 \| 1.246763 \| \| H \| 6.600249 \| −3.76935 \| 0.037078 \| H \| −6.58546 \| 3.747053 \| 0.427135 \| \| C \| −5.96742 \| −2.99063 \| 2.23515 \| C \| 6.03226 \| 3.241935 \| 1.79141 \| \| H \| −5.55114 \| −3.82651 \| 2.789337 \| H \| 5.644963 \| 4.15791 \| 2.225567 \| \| C \| 5.44093 \| 2.456665 \| 2.944603 \| C \| −5.42937 \| −2.54306 \| 2.872049 \| \| H \| 5.208856 \| 1.386812 \| 2.954433 \| H \| −5.18183 \| −1.47919 \| 2.936329 \| \| H \| 5.086211 \| 2.881249 \| 3.890298 \| H \| −5.07112 \| −3.02815 \| 3.787565 \| \| H \| 6.530046 \| 2.562855 \| 2.918731 \| H \| −6.52031 \| −2.63219 \| 2.85156 \| \| H \| −9.19666 \| 2.742018 \| −1.43484 \| H \| 9.096046 \| −3.06145 \| −0.93847 \| \| H \| −7.99933 \| −3.46513 \| 2.788798 \| H \| 8.078961 \| 3.717415 \| 2.274067 \| |
| --- | --- | --- | --- | --- | --- | --- | --- | --- | --- | --- | --- | --- | --- | --- | --- | --- | --- | --- | --- | --- | --- | --- | --- | --- | --- | --- | --- | --- | --- | --- | --- | --- | --- | --- | --- | --- | --- | --- | --- | --- | --- | --- | --- | --- | --- | --- | --- | --- | --- | --- | --- | --- | --- | --- | --- | --- | --- | --- | --- | --- | --- | --- | --- | --- | --- | --- | --- | --- | --- | --- | --- | --- | --- | --- | --- | --- | --- | --- | --- | --- | --- | --- | --- | --- | --- | --- | --- | --- | --- | --- | --- | --- | --- | --- | --- | --- | --- | --- | --- | --- | --- | --- | --- | --- | --- | --- | --- | --- | --- | --- | --- | --- | --- | --- | --- | --- | --- | --- | --- | --- | --- | --- | --- | --- | --- | --- | --- | --- | --- | --- | --- | --- | --- | --- | --- | --- | --- | --- | --- | --- | --- | --- | --- | --- | --- | --- | --- | --- | --- | --- | --- | --- | --- | --- | --- | --- | --- | --- | --- | --- | --- | --- | --- | --- | --- | --- | --- | --- | --- | --- | --- | --- | --- | --- | --- | --- | --- | --- | --- | --- | --- | --- | --- | --- | --- | --- | --- | --- | --- | --- | --- | --- | --- | --- | --- | --- | --- | --- | --- | --- | --- | --- | --- | --- | --- | --- | --- | --- | --- | --- | --- | --- | --- | --- | --- | --- | --- | --- | --- | --- | --- | --- | --- | --- | --- | --- | --- | --- | --- | --- | --- | --- | --- | --- | --- | --- | --- | --- | --- | --- | --- | --- | --- | --- | --- | --- | --- | --- | --- | --- | --- | --- | --- | --- | --- | --- | --- | --- | --- | --- | --- | --- | --- | --- | --- | --- | --- | --- | --- | --- | --- | --- | --- | --- | --- | --- | --- | --- | --- | --- | --- | --- | --- | --- | --- | --- | --- | --- | --- | --- | --- | --- | --- | --- | --- | --- | --- | --- | --- | --- | --- | --- | --- | --- | --- | --- | --- | --- | --- | --- | --- | --- | --- | --- | --- | --- | --- | --- | --- | --- | --- | --- | --- | --- | --- | --- | --- | --- | --- | --- | --- | --- | --- | --- | --- | --- | --- | --- | --- | --- | --- | --- | --- | --- | --- | --- | --- | --- | --- | --- | --- | --- | --- | --- | --- | --- | --- | --- | --- | --- | --- | --- | --- | --- | --- | --- | --- | --- | --- | --- | --- | --- | --- | --- | --- | --- | --- | --- | --- | --- | --- | --- | --- | --- | --- | --- | --- | --- | --- | --- | --- | --- | --- | --- | --- | --- | --- | --- | --- | --- | --- | --- | --- | --- | --- | --- | --- | --- | --- | --- | --- | --- | --- | --- | --- | --- | --- | --- | --- | --- | --- | --- | --- | --- | --- | --- | --- | --- | --- | --- | --- | --- | --- | --- | --- | --- | --- | --- | --- | --- | --- | --- | --- | --- | --- | --- | --- | --- | --- | --- | --- | --- | --- | --- | --- | --- | --- | --- | --- | --- | --- | --- | --- | --- | --- | --- | --- | --- | --- | --- | --- | --- | --- | --- | --- | --- | --- | --- | --- | --- | --- | --- | --- | --- | --- | --- | --- | --- | --- | --- | --- | --- | --- | --- | --- | --- | --- | --- | --- | --- | --- | --- | --- | --- | --- | --- | --- | --- | --- | --- | --- | --- | --- | --- | --- | --- | --- | --- | --- | --- | --- | --- | --- | --- | --- | --- | --- | --- | --- | --- | --- | --- | --- | --- | --- | --- | --- | --- | --- | --- | --- | --- | --- | --- | --- | --- | --- | --- | --- | --- | --- | --- | --- | --- | --- | --- | --- | --- | --- | --- | --- | --- | --- | --- | --- | --- | --- | --- | --- | --- | --- | --- | --- | --- | --- | --- | --- | --- | --- | --- | --- | --- | --- | --- | --- | --- | --- | --- | --- | --- | --- | --- | --- | --- | --- | --- | --- | --- | --- | --- | --- | --- | --- | --- | --- | --- | --- | --- | --- | --- | --- | --- | --- | --- | --- | --- | --- | --- | --- | --- | --- | --- | --- | --- | --- | --- | --- | --- | --- | --- | --- | --- | --- | --- | --- | --- | --- | --- | --- | --- | --- | --- | --- | --- | --- | --- | --- | --- | --- | --- | --- | --- | --- | --- | --- | --- | --- | --- | --- | --- | --- | --- | --- | --- | --- | --- | --- | --- | --- | --- | --- | --- | --- | --- | --- | --- | --- | --- | --- | --- | --- | --- | --- | --- | --- | --- | --- | --- | --- | --- | --- | --- | --- | --- | --- | --- | --- | --- | --- | --- | --- | --- | --- | --- | --- | --- | --- | --- | --- | --- | --- | --- | --- | --- | --- | --- | --- | --- | --- | --- | --- | --- | --- | --- | --- | --- | --- | --- | --- | --- | --- | --- | --- | --- | --- | --- |

**Table S7.** Cartesian coordinates of the optimized ground-state (S0) and excited state (T1) geometry of **Pxz-dfppy-CN**.

| Ground state of **Pxz-dfppy-CN** (S_0_) | Excited state of **Pxz-dfppy-CN** (T_1_) |
| --- | --- |

| Ir | 1.095157 | −0.70782 | 0.584318 | Ir | −1.08922 | −0.5927 | −0.70002 |
| --- | --- | --- | --- | --- | --- | --- | --- |
| N | 3.264837 | −0.7826 | 0.663692 | N | −3.25853 | −0.66679 | −0.78609 |
| F | 0.351152 | 3.451122 | −2.88472 | F | −0.35666 | 2.912266 | 3.429864 |
| F | 4.77748 | 2.297393 | −1.88552 | F | −4.78013 | 1.918571 | 2.259876 |
| N | −0.6522 | −1.97317 | −1.37543 | N | 0.671817 | −2.16273 | 1.011523 |
| N | 1.278499 | −2.79006 | −1.88456 | N | −1.25268 | −3.06663 | 1.378767 |
| N | 1.236076 | 1.353555 | 3.072839 | N | −1.25293 | 1.863097 | −2.80075 |
| N | −0.68283 | 0.982608 | 2.159075 | N | 0.671545 | 1.352884 | −1.9691 |
| C | 1.61299 | 0.827701 | −0.69076 | C | −1.6117 | 0.697609 | 0.820588 |
| N | 0.885591 | −3.18137 | 2.646055 | N | −0.86569 | −2.67777 | −3.15439 |
| C | 0.681688 | −1.94851 | −1.01203 | C | −0.66379 | −2.08507 | 0.660843 |
| C | −1.54026 | −1.16639 | −0.62252 | C | 1.551419 | −1.23603 | 0.403786 |
| C | 3.966988 | −1.66323 | 1.400729 | C | −3.95822 | −1.41124 | −1.66245 |
| H | 3.372165 | −2.36298 | 1.976522 | H | −3.36137 | −1.99628 | −2.35263 |
| C | −0.85779 | −0.49151 | 0.38688 | C | 0.863056 | −0.40091 | −0.47397 |
| C | 3.911314 | 0.144495 | −0.10157 | C | −3.90791 | 0.109988 | 0.12975 |
| C | 0.654446 | 0.639002 | 2.08471 | C | −0.6639 | 0.993423 | −1.95087 |
| C | 3.017804 | 1.03459 | −0.85014 | C | −3.01718 | 0.86425 | 1.017384 |
| C | 2.679483 | 1.399306 | 3.298686 | C | −2.69757 | 1.940609 | −3.00858 |
| H | 3.07495 | 0.41638 | 3.040111 | H | −3.0877 | 0.92552 | −2.92651 |
| H | 2.848819 | 1.560669 | 4.367204 | H | −2.87268 | 2.287541 | −4.03105 |
| C | 2.724238 | −2.99292 | −1.95597 | C | −2.69719 | −3.28599 | 1.419596 |
| H | 2.904262 | −4.01269 | −2.30834 | H | −2.87131 | −4.35205 | 1.591928 |
| H | 3.109164 | −2.91633 | −0.93843 | H | −3.0868 | −3.03699 | 0.431877 |
| C | 0.902858 | −2.2814 | 1.896443 | C | −0.8901 | −1.91898 | −2.26229 |
| C | 0.354468 | −3.32338 | −2.78259 | C | −0.32326 | −3.73836 | 2.171685 |
| H | 0.645375 | −4.01802 | −3.55379 | H | −0.60814 | −4.55481 | 2.815245 |
| C | −0.86197 | −2.814 | −2.4579 | C | 0.889588 | −3.1736 | 1.935759 |
| H | −1.83631 | −2.98245 | −2.88534 | H | 1.865794 | −3.40613 | 2.327385 |
| C | 5.35435 | −1.67641 | 1.423402 | C | −5.34544 | −1.43011 | −1.68315 |
| H | 5.877166 | −2.40463 | 2.03234 | H | −5.86596 | −2.04544 | −2.40761 |
| C | −1.55657 | 0.376453 | 1.223019 | C | 1.551233 | 0.601797 | −1.15423 |
| C | −2.90871 | −0.9934 | −0.81377 | C | 2.922682 | −1.09273 | 0.61702 |
| H | −3.4648 | −1.50064 | −1.59439 | H | 3.488438 | −1.72063 | 1.295618 |
| C | 0.730109 | 1.659976 | −1.39101 | C | −0.7311 | 1.402502 | 1.651155 |
| H | −0.34308 | 1.536672 | −1.29793 | H | 0.342376 | 1.304071 | 1.535078 |
| C | 0.299467 | 2.137002 | 3.746449 | C | −0.32367 | 2.755842 | −3.33294 |
| H | 0.5778 | 2.777073 | 4.567846 | H | −0.60907 | 3.525437 | −4.03168 |
| C | −2.92512 | 0.583134 | 1.072313 | C | 2.922574 | 0.788615 | −0.97814 |
| H | −3.4936 | 1.25657 | 1.704095 | H | 3.4883 | 1.560308 | −1.48725 |
| C | −0.90943 | 1.898855 | 3.175048 | C | 0.889202 | 2.430693 | −2.8144 |
| H | −1.88865 | 2.284024 | 3.405918 | H | 1.865193 | 2.856288 | −2.97819 |
| C | 3.391869 | −1.95984 | −2.86466 | C | −3.36471 | −2.42767 | 2.494914 |
| H | 3.096179 | −0.96379 | −2.52145 | H | −3.07465 | −1.38611 | 2.326545 |
| H | 3.004538 | −2.06773 | −3.88582 | H | −2.9716 | −2.70723 | 3.480676 |
| C | 5.316621 | 0.173279 | −0.11315 | C | −5.31341 | 0.12608 | 0.150253 |
| H | 5.829235 | 0.904821 | −0.71646 | H | −5.82813 | 0.738468 | 0.872684 |
| C | 1.219544 | 2.659459 | −2.21837 | C | −1.22307 | 2.241515 | 2.639678 |
| C | −3.58414 | −0.11292 | 0.046042 | C | 3.574031 | −0.0745 | −0.08907 |
| C | 3.34461 | 2.487379 | 2.454747 | C | −3.36306 | 2.858886 | −1.98259 |
| H | 3.059293 | 2.328712 | 1.410382 | H | −3.07167 | 2.51962 | −0.98391 |
| H | 2.945429 | 3.467999 | 2.743621 | H | −2.96952 | 3.877153 | −2.095 |
| C | 6.037211 | −0.73693 | 0.649342 | C | −6.03126 | −0.64367 | −0.75587 |
| H | 7.122063 | −0.71153 | 0.637554 | H | −7.11619 | −0.62844 | −0.73669 |
| C | 2.580753 | 2.888843 | −2.39721 | C | −2.58493 | 2.42772 | 2.85914 |
| H | 2.95161 | 3.671756 | −3.04556 | H | −2.95794 | 3.084978 | 3.633562 |
| C | 3.446109 | 2.063081 | −1.70238 | C | −3.44808 | 1.728148 | 2.035124 |
| C | 4.869719 | 2.480834 | 2.582722 | C | −4.88875 | 2.867454 | −2.10309 |
| H | 5.241124 | 1.465004 | 2.402914 | H | −5.25506 | 1.834042 | −2.10218 |
| H | 5.155602 | 2.737597 | 3.61112 | H | −5.18113 | 3.298838 | −3.06939 |
| C | 4.917851 | −2.07653 | −2.86522 | C | −4.89021 | −2.55024 | 2.482027 |
| H | 5.215032 | −3.03946 | −3.30082 | H | −5.18119 | −3.57518 | 2.746819 |
| H | 5.278274 | −2.08122 | −1.82972 | H | −5.25602 | −2.37848 | 1.462794 |
| C | 5.537132 | 3.445345 | 1.599336 | C | −5.55493 | 3.641808 | −0.96317 |
| H | 5.19134 | 4.472879 | 1.757499 | H | −5.21381 | 4.682744 | −0.94033 |
| H | 5.305627 | 3.17381 | 0.564319 | H | −5.31738 | 3.194218 | 0.007271 |
| H | 6.625899 | 3.438477 | 1.711834 | H | −6.64421 | 3.649795 | −1.07056 |
| C | 5.585662 | −0.9302 | −3.62864 | C | −5.55867 | −1.55561 | 3.434254 |
| H | 5.342699 | 0.036923 | −3.17675 | H | −5.32233 | −0.52399 | 3.154179 |
| H | 5.25093 | −0.90207 | −4.67151 | H | −5.21824 | −1.7054 | 4.46484 |
| H | 6.675245 | −1.03461 | −3.63051 | H | −6.64778 | −1.6644 | 3.42333 |
| C | −5.91519 | −0.69644 | 0.55872 | C | 5.880397 | −0.57447 | −0.67676 |
| C | −5.44261 | 1.12737 | −0.96988 | C | 5.409981 | 0.953804 | 1.124548 |
| C | −5.52772 | −1.72039 | 1.428318 | C | 5.493366 | −1.45498 | −1.71601 |
| C | −7.28974 | −0.45597 | 0.379356 | C | 7.296209 | −0.37656 | −0.44116 |
| C | −6.82771 | 1.328946 | −1.1162 | C | 6.83861 | 1.119042 | 1.321315 |
| C | −4.57247 | 1.972136 | −1.66619 | C | 4.533449 | 1.673165 | 1.972498 |
| C | −6.48671 | −2.4845 | 2.100503 | C | 6.441942 | −2.09995 | −2.47545 |
| H | −4.47218 | −1.91579 | 1.574863 | H | 4.437395 | −1.61177 | −1.90277 |
| C | −8.24116 | −1.21208 | 1.046886 | C | 8.248326 | −1.03896 | −1.2208 |
| C | −7.32174 | 2.338964 | −1.92768 | C | 7.327623 | 1.965286 | 2.32013 |
| H | −3.50387 | 1.827326 | −1.56271 | H | 3.46439 | 1.5587 | 1.836485 |
| C | −5.06997 | 2.989955 | −2.48594 | C | 5.02875 | 2.502676 | 2.951569 |
| C | −7.84305 | −2.23542 | 1.914398 | C | 7.844659 | −1.89361 | −2.22961 |
| H | −6.15919 | −3.27391 | 2.769312 | H | 6.127741 | −2.77072 | −3.26741 |
| H | −9.2884 | −0.98669 | 0.874978 | H | 9.297654 | −0.86037 | −1.00845 |
| C | −6.44199 | 3.178361 | −2.62045 | C | 6.450633 | 2.656636 | 3.134664 |
| H | −8.39776 | 2.453232 | −2.00605 | H | 8.403475 | 2.057783 | 2.430658 |
| H | −4.37218 | 3.630779 | −3.01546 | H | 4.342233 | 3.045666 | 3.591699 |
| H | −8.592 | −2.8244 | 2.433132 | H | 8.583042 | −2.40757 | −2.83457 |
| H | −6.83492 | 3.966186 | −3.25436 | H | 6.830137 | 3.313052 | 3.909468 |
| N | −4.98551 | 0.092299 | −0.13865 | N | 4.985519 | 0.105011 | 0.123186 |
| O | −7.75358 | 0.534506 | −0.46683 | O | 7.728488 | 0.455324 | 0.540164 |

# Device Optimization


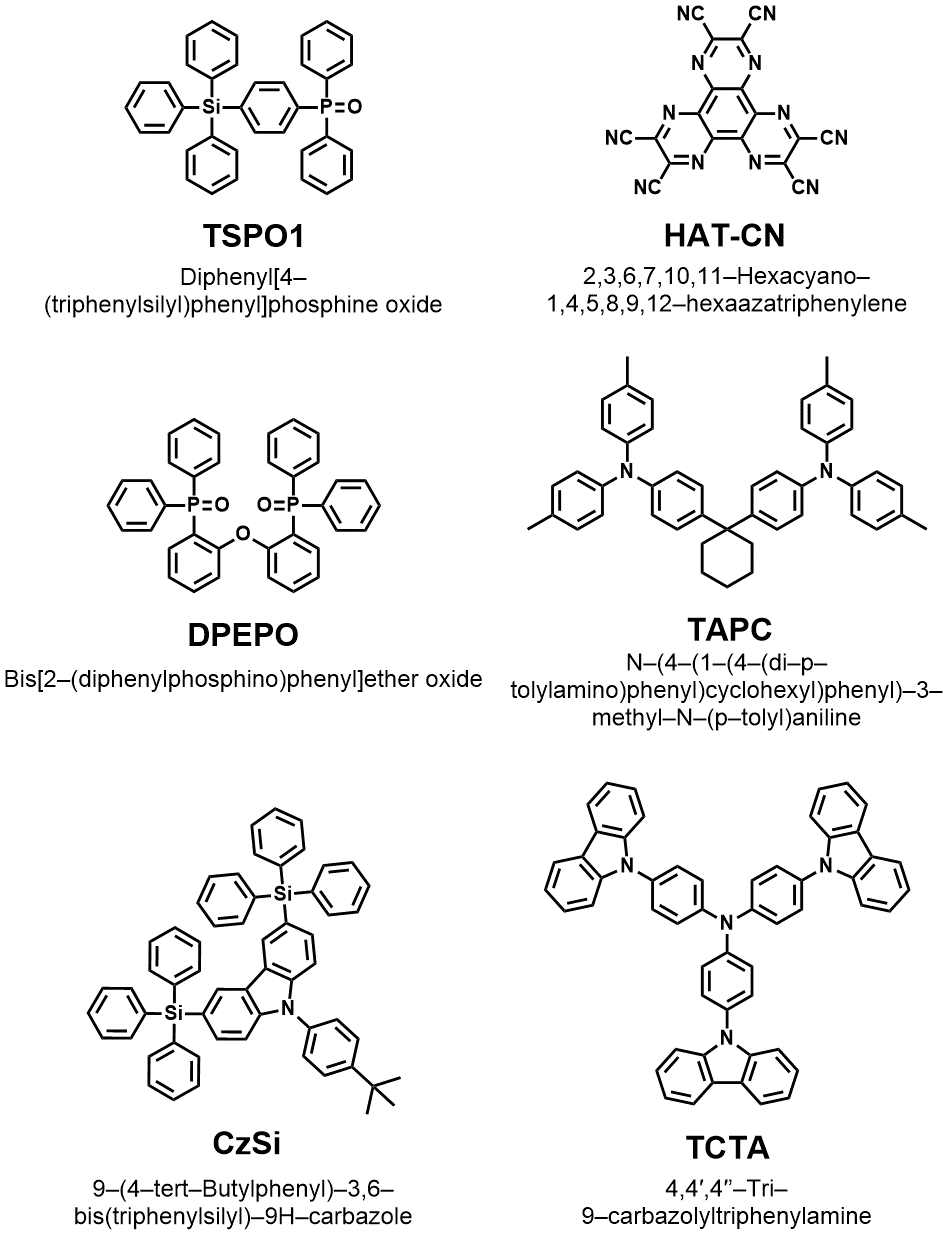


**Scheme S1.** Acronyms, IUPAC names and molecular structures of the materials used in the studied OLED devices.


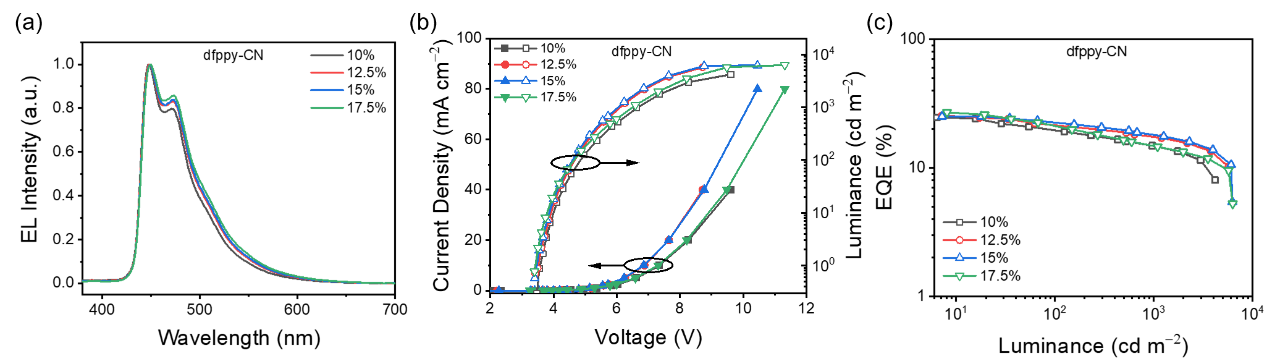


**Figure S18.** (a) EL spectra; (b) J-V-L characteristics; (c) EQE vs. luminance plot of Ph-OLED featuring **dfppy-CN**.


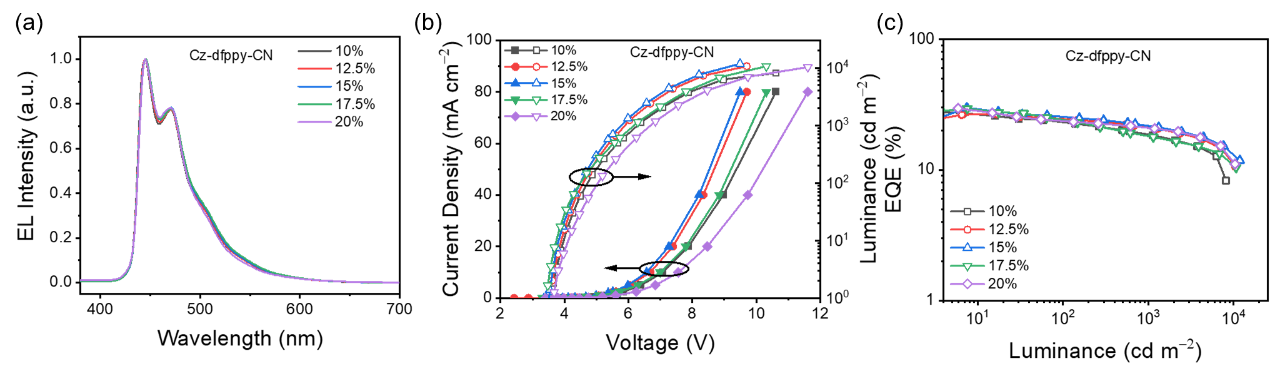


**Figure S19.** (a) EL spectra; (b) J-V-L characteristics; (c) EQE vs. luminance plot of Ph-OLED featuring **Cz-dfppy-CN**.


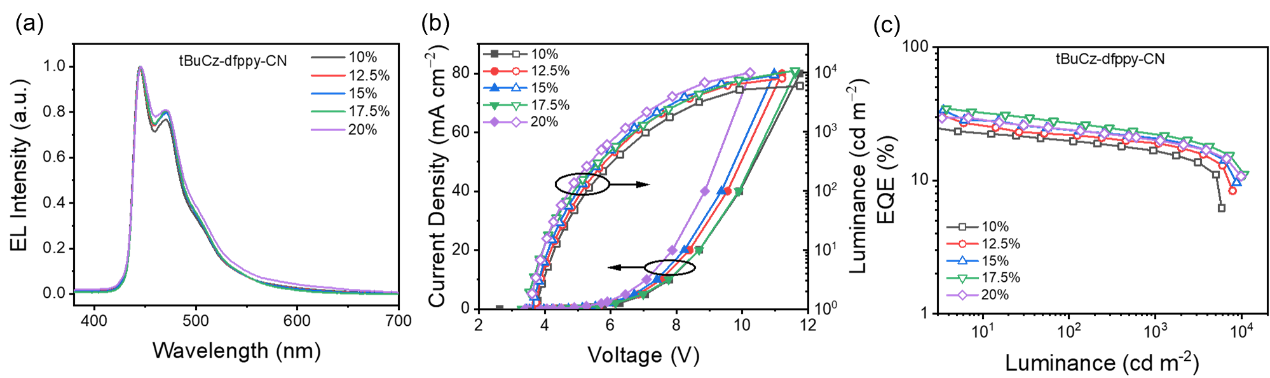


**Figure S20.** (a) EL spectra; (b) J-V-L characteristics; (c) EQE vs. luminance plot of Ph-OLED featuring **tBuCz-dfppy-CN**.


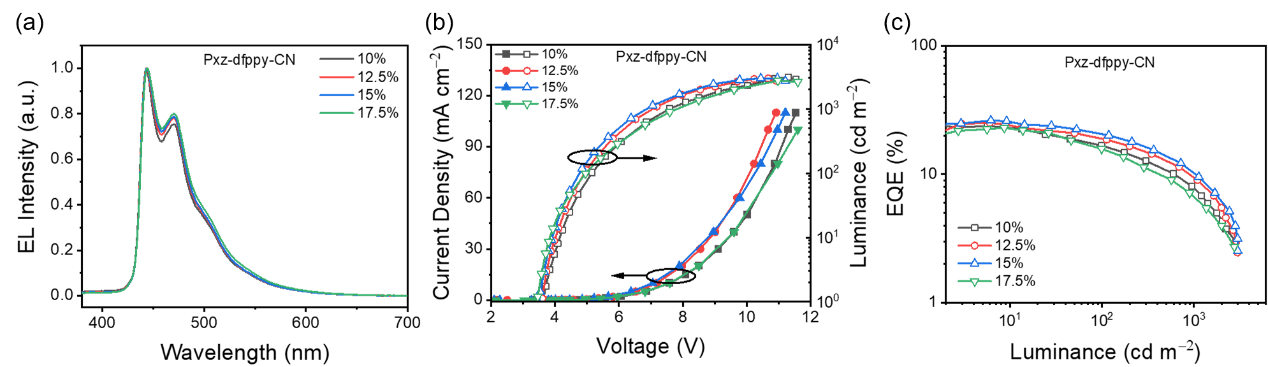


**Figure S21.** (a) EL spectra; (b) J-V-L characteristics; (c) EQE vs. luminance plot of Ph-OLED featuring **Pxz-dfppy-CN**.

**Table S8.** Summarized Ph-OLED device optimization results.

| Doping Conc.  in CzSi | V_on_^a^  [V] | L_max_  [cd m^−2^] | max. EQE/CE/PE  [%/cd A^−1^/Im W^−1^] | EQE/CE/PE  @1000 cd m^−2^ | λ_EL_  [nm] | FWHM ^c^  [nm] | CIE ^d^  [x, y] |
| --- | --- | --- | --- | --- | --- | --- | --- |
| dfppy-CN |  |  |  |  |  |  |  |
| 10wt% | 3.6 | 4176 | 24.2/31.4/24.1 | 15.2/19.2/9.1 | 449/471 | 51.2 | 0.142, 0.160 |
| 12.5wt% | 3.55 | 5700 | 24.5/34.5/26.8 | 17.9/24.5/12.7 | 450/472 | 54.1 | 0.144, 0.166 |
| 15wt% | 3.5 | 6222 | 25.0/35.9/28.2 | 18.6/26.0/13.6 | 448/474 | 55.0 | 0.144, 0.171 |
| 17.5wt% (Device I) | 3.45 | 6285 | 25.8/40.4/33.9 | 15.4/22.2/10.8 | 450/473 | 56.0 | 0.145, 0.178 |
| Cz-dfppy-CN |  |  |  |  |  |  |  |
| 10%wt | 3.6 | 8140 | 27.3/33.5/27.1 | 19.1/23.1/11.6 | 444/470 | 48.4 | 0.143, 0.133 |
| 12.5%wt | 3.55 | 10492 | 26.5/32.6/26.9 | 21.6/26.2/14.0 | 445/470 | 48.5 | 0.142, 0.136 |
| 15%wt | 3.5 | 11805 | 27.8/34.9/27.8 | 22.1/27.3/15.0 | 445/470 | 49.3 | 0.143, 0.137 |
| 17.5%wt (Device II) | 3.4 | 10677 | 29.5/37.8/32.2 | 18.4/23.3/11.9 | 445/471 | 49.5 | 0.143, 0.135 |
| 20%wt | 3.55 | 10314 | 27.3/29.7/22.0 | 20.8/23.6/10.8 | 445/471 | 48.6 | 0.141,0.143 |
| tBuCz-dfppy-CN |  |  |  |  |  |  |  |
| 10%wt | 3.6 | 5860 | 22.8/25.8/19.8 | 16.7/18.9/8.4 | 444/470 | 47.4 | 0.142, 0.138 |
| 12.5%wt | 3.7 | 7878 | 26.2/30.0/23.4 | 19.1/21.8/10.2 | 445/470 | 49.3 | 0.142, 0.145 |
| 15%wt | 3.6 | 8865 | 27.5/30.8/24.4 | 20.1/23.1/11.1 | 445/470 | 48.4 | 0.141, 0.144 |
| 17.5wt% (Device III) | 3.5 | 10710 | 30.7/36.7/28.7 | 21.2/25.2/11.2 | 445/471 | 49.1 | 0.140, 0.148 |
| 20%wt | 3.6 | 9868 | 29.5/33.9/27.6 | 19.8/22.8/11.1 | 445/470 | 51.2 | 0.144, 0.143 |
| Pxz-dfppy-CN |  |  |  |  |  |  |  |
| 10%wt | 3.7 | 3986 | 21.9/26.0/19.9 | 8.3/10.0/4.1 | 443/470 | 46.4 | 0.142, 0.141 |
| 12.5%wt | 3.6 | 3235 | 22.3/26.4/21.0 | 9.7/11.6/5.3 | 444/471 | 48.4 | 0.143, 0.138 |
| 15wt% (Device IV) | 3.5 | 3297 | 24.3/28.4/23.1 | 10.2/12.1/5.6 | 444/471 | 48.8 | 0.143, 0.142 |
| 17.5%wt | 3.5 | 2985 | 23.6/28.4/23.5 | 7.5/9.2/3.8 | 444/470 | 50.3 | 0.143, 0.141 |


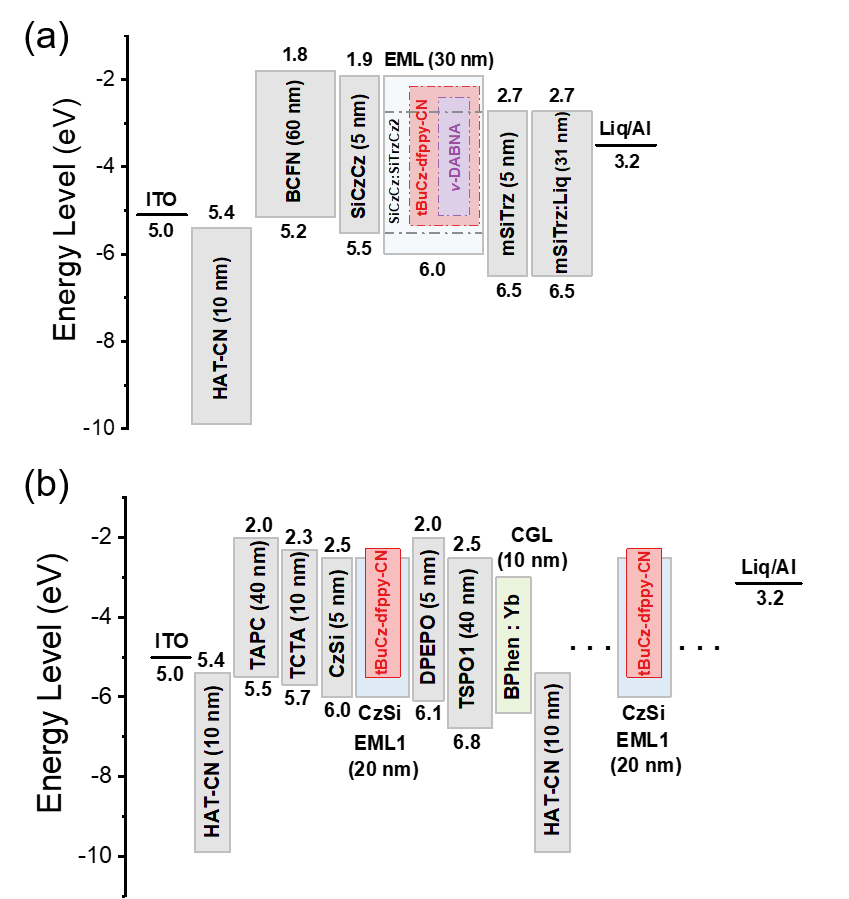


**Figure S22.** Device strucutre of (a) hyper-OLED (Device VI) and (b) tandem OLED (Device V).


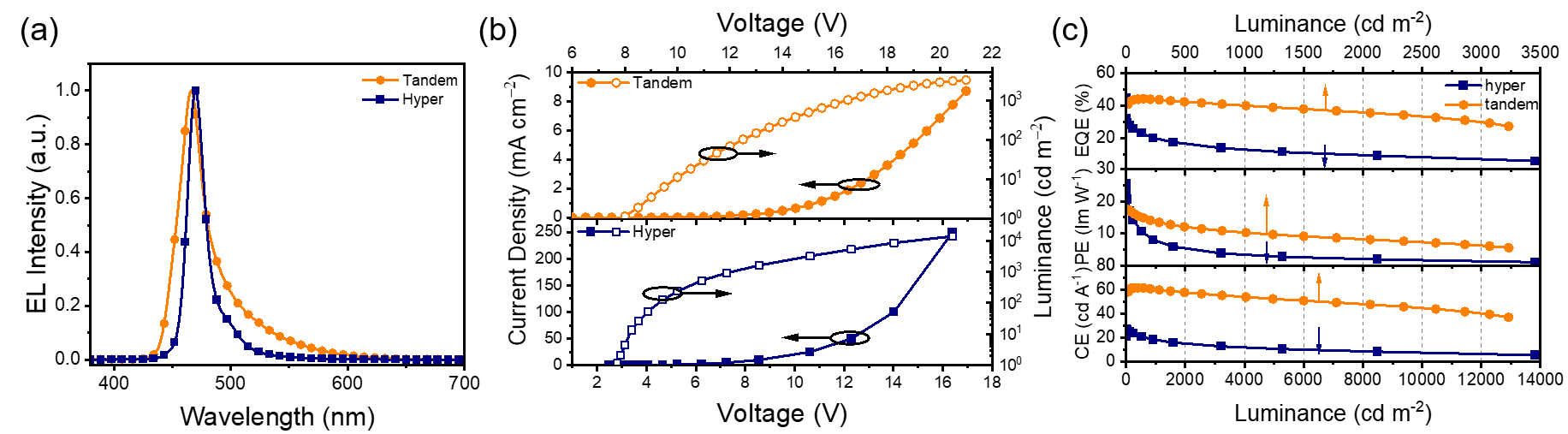


**Figure S23.** (a) EL spectra, (b) J-V-L characteristics and (c) Efficiency vs. Luminance plot of tandem device (Device V) and hyper device (Device VI).

**Figure S24.** Operational lifetime of hyper-OLED using **tBuCz-dfppy-CN** as the sensitizer.

# Experimental Section

**Materials**. All reagents and solvents were commercially available and were employed without further purification.

**Characterization Methods**. Bruker 400, 500 or 600 MHz NMR spectrometers were used to record the ^1^H, ^19^F and ^13^C NMR spectra of the studied complexes in CDCl_3_ and DMSO-*d_6_* solution. The chemical shifts (δ) of the signals are given in ppm and referenced to the internal standard tetramethylsilane (TMS). The signals splitting is abbreviated as follows: s = singlet; d = doublet; t = triplet; q = quartet; m = multiplet. All coupling constants (J) are given in Hertz (Hz). Electrospray ionization (ESI) mass spectrum was recorded on a Thermo Scientific Exactive Benchtop LC/MS Orbitrap Mass Spectrometer. The absorption and phosphorescence spectra were performed using a UV-vis spectrophotometer (Cary 5000 UV–vis–NIR, Agilent, USA) and a spectrofluorometer (Edinburgh Instruments Ltd FS5), respectively. The emitting dipole orientation of a doped film was determined by angle-resolved *p*-polarized PL measurements in a Hamamatsu’s established molecular orientation characteristic measurement system (C14234-11, Hamamatsu Photonics). Electrochemical measurements were performed with a PalmSens4 electrochemical work station with platinum-carbon as working electrode, platinum wire as the counter electrode, and a saturated calomel electrode (SCE) in saturated KCl aqueous solution as the reference electrode. The cyclic voltammogram was referenced to the ferrocene/ferrocenium couple at a scan rate of 100 mV s^-1^. Thermogravimetric analysis (TGA) was performed on a Mettler TGA2 thermogravimeter by measuring the weight loss from 25 ℃ to 100 ℃ at a rate of 10 ℃/min under nitrogen. After 15 minutes, heating from 100 ℃ to 800 ℃ at a rate of 10 ℃/min under nitrogen.

**Device Fabrication**. The ITO (indium-tin oxide) coated glass substrates were first cleaned in deionized water, acetone, and ethanol, then dried in an oven and treated by ultraviolet ozone exposure for 20 min. Device fabrication were performed with a FS- 450(Suzhou Fangsheng) chamber. All organic layers were thermally evaporated at a rate of 0.5-1.5 Å s^-1^ at a pressure of ca. 7.5 × 10^-7^ Torr. A Liq layer (2.5 nm) was deposited at a rate of 0.2 Å s^-1^. The Al cathode was deposited at a rate of 4 Å s^-1^; the active area of the diode segments was 3×3 mm^2^. The PHOLEDs devices performance including EL spectra, current density-voltage-luminance (*J-V-L*) curves and Commission Internationale de L'Eclairage (CIE) coordinates were characterized by a Keithley 2400 semiconductor characterization system.

# Material Synthesis


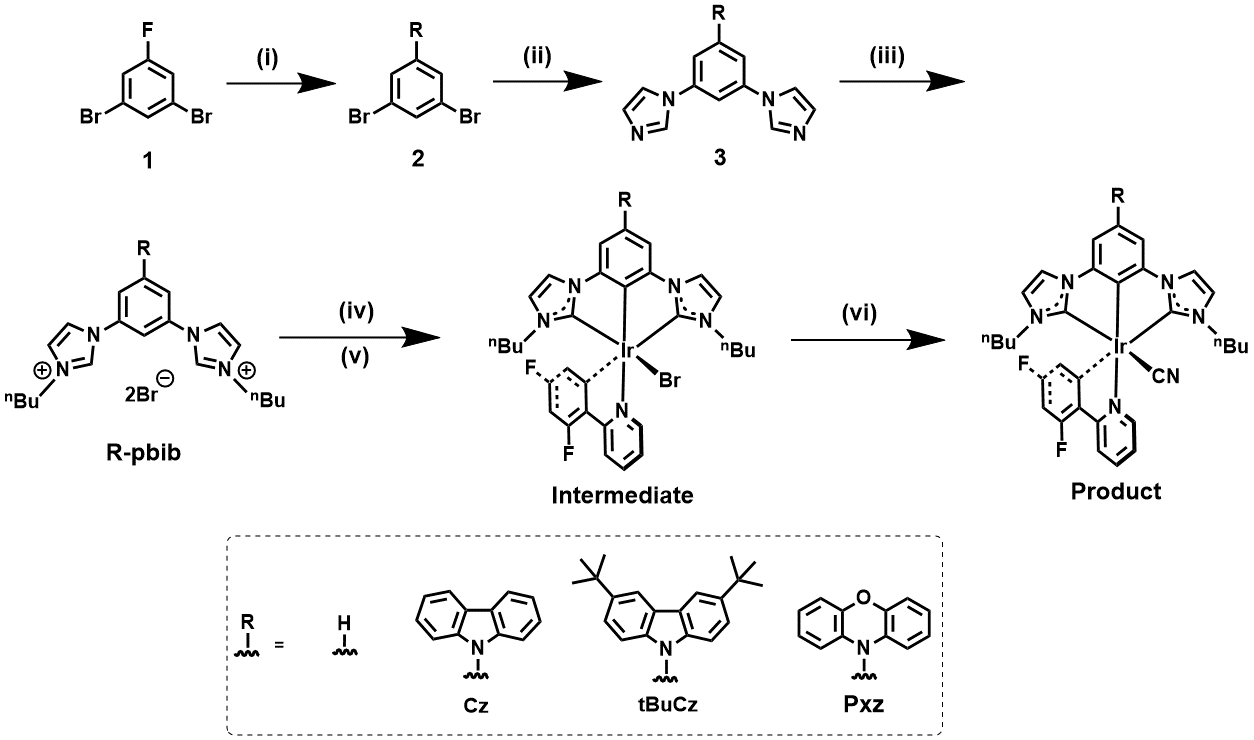


**Scheme S2.** General synthesis procedure of the [3+2+1] Ir(III) complexes. (i) Cs_2_CO_3_, (R = Cz, tBuCz and Pxz), DMF, 150 ℃ 48h; (ii) CuO, K_2_CO_3_, DMSO, 150 ℃; (iii) MeCN, 90 ℃; (iv) [Ir(cod)Cl]_2_, MeCN, 90 ℃; (v) dfppy, propionic acid, 150 ℃; (vi) AgCN, DMF, 100 ℃.

Synthesis of **9-(3,5-dibromophenyl)-9H-carbazole**:

1,3-Dibromo-5-fluorobenzene (4.0 g, 15.8 mmol, 1.0 eq), 9H-carbazole (3.2 g, 19.1 mmol, 1.2 eq), Cesium carbonate (7.7 g, 31.6 mmol, 1.5 eq) were refluxed with 100 ml DMF at a temperature of 150 ℃ for 48 h under nitrogen atmosphere. The solid in the mixture was filtered off and the liquid is washed with water and extracted with DCM. The organic phase was then dried with anhydrous MgSO_4_ and concentrated under reduced pressure. The crude product was purified through column chromatography on silica gel using petroleum ether as eluent to obtain the desired product 9-(3,5-dibromophenyl)-9H-carbazole as white powder (5.2 g, 82%). ^1^H NMR (500 MHz, DMSO-*d_6_*) δ 8.25 (d, J = 7.7 Hz, 2H), 8.03 (t, J = 1.7 Hz, 1H), 7.92 (d, J = 1.7 Hz, 2H), 7.47 (ddd, J = 8.1, 6.9, 1.2 Hz, 2H), 7.42 (d, J = 8.1 Hz, 2H), 7.35 – 7.29 (m, 2H).

Synthesis of **9-(3,5-di(1H-imidazol-1-yl)phenyl)-9H-carbazole**:

9-(3,5-Dibromophenyl)-9H-carbazole (2.5 g, 6.27 mmol, 1.0 eq), imidazole (1.07 g, 15.68 mmol, 2.5 eq), potassium carbonate (2.17 g, 15.68 mmol, 2.5 eq) and Cupric oxide (0.5 g, 20%) was dissolved in 50 ml DMSO then refluxed at a temperature of 150 ℃ for 48 h under nitrogen atmosphere. The mixture was then filtered to remove the solid residue and the filtrate was washed with water and extracted with DCM. The organic phase was collected and dried with anhydrous MgSO_4_. The solution was concentrated under reduced pressure and petroleum ether was added and filtered to obtain 9-(3,5-di(1H-imidazol-1-yl)phenyl)-9H-carbazole white solid (1.33 g, 56%). ^1^H NMR (400 MHz, DMSO-*d_6_*) δ 8.56 (s, 2H), 8.28 (dd, J = 7.7, 1.1 Hz, 2H), 8.17 (t, J = 2.0 Hz, 1H), 8.05 (s, 2H), 8.01 (d, J = 2.0 Hz, 2H), 7.57 (d, J = 8.2 Hz, 2H), 7.47 (ddd, J = 8.3, 7.1, 1.2 Hz, 2H), 7.37 – 7.30 (m, 2H), 7.17 (s, 2H).

Synthesis of **1,1'-(5-(9H-carbazol-9-yl)-1,3-phenylene)bis(3-butyl-1H-imidazol-3-ium) dibromide (Cz-pbib)**:

9-(3,5-Di(1H-imidazol-1-yl)phenyl)-9H-carbazole (1.36 g, 3.62 mmol, 1.0 eq) and 1-bromobutane (2.5g, 18.2 mmol, 5.0 eq) were dissolved in 50 ml acetonitrile and refluxed at a temperature of 100 ℃ under nitrogen atmosphere for 24 h. After cooling to room temperature, the volatiles were removed under reduced pressure, then diethyl ether was added and filtered to afford the product as white solid (2.25 g, 91%). ^1^H NMR (400 MHz, DMSO-*d_6_*) δ 10.15 (s, 2H), 8.61 (p, J = 1.9 Hz, 2H), 8.59 – 8.55 (m, 1H), 8.41 (d, J = 1.9 Hz, 2H), 8.32 (dt, J = 7.8, 1.0 Hz, 2H), 8.16 (t, J = 1.8 Hz, 2H), 7.70 (d, J = 8.2 Hz, 2H), 7.55 – 7.48 (m, 2H), 7.39 (ddd, J = 8.0, 7.3, 0.9 Hz, 2H), 4.31 (t, J = 7.2 Hz, 4H), 1.97 – 1.84 (m, 4H), 1.42 – 1.29 (m, 4H), 0.94 (t, J = 7.4 Hz, 6H).

Synthesis of **3,6-di-tert-butyl-9-(3,5-dibromophenyl)-9H-carbazole**:

The synthetic protocol of **3,6-di-tert-butyl-9-(3,5-dibromophenyl)-9H-carbazole** is the same as that of **9-(3,5-di(1H-imidazol-1-yl)phenyl)-9H-carbazole** except using 3,6-di-*tert*-butyl-9H-carbazole as reactant. ^1^H NMR (400 MHz, DMSO-*d_6_*) δ 8.29 (d, J = 2.0 Hz, 2H), 7.97 (t, J = 1.7 Hz, 1H), 7.88 (d, J = 1.7 Hz, 2H), 7.51 (d, J = 2.0 Hz, 1H), 7.49 (d, J = 2.0 Hz, 1H), 7.35 (s, 1H), 7.33 (s, 1H), 1.41 (s, 18H).

Synthesis of **3,6-di-tert-butyl-9-(3,5-di(1H-imidazol-1-yl)phenyl)-9H-carbazole**:

The synthetic protocol of **3,6-di-tert-butyl-9-(3,5-di(1H-imidazol-1-yl)phenyl)-9H-carbazole** is the same as that of **9-(3,5-di(1H-imidazol-1-yl)phenyl)-9H-carbazole** as reactant. ^1^H NMR (400 MHz, DMSO-*d_6_*) δ 8.55 (s, 2H), 8.32 (s, 2H), 8.12 (t, J = 2.0 Hz, 1H), 8.04 (t, J = 1.4 Hz, 2H), 7.97 (d, J = 2.0 Hz, 2H), 7.54 – 7.46 (m, 4H), 7.16 (s, 2H), 1.43 (s, 18H).

Synthesis of **1,1'-(5-(3,6-di-tert-butyl-9H-carbazol-9-yl)-1,3-phenylene)bis(3-butyl-1H-imidazol-3-ium) (tBuCz-pbib)**:

The synthetic protocol of **1,1'-(5-(3,6-di-tert-butyl-9H-carbazol-9-yl)-1,3-phenylene)bis(3-butyl-1H-imidazol-3-ium)** is the same as that of **1,1'-(5-(9H-carbazol-9-yl)-1,3-phenylene)bis(3-butyl-1H-imidazol-3-ium) dibromide** except using 3,6-di-*tert*-butyl-9-(3,5-di(1H-imidazol-1-yl)phenyl)-9H-carbazole as reagent. ^1^H NMR (400 MHz, DMSO-*d_6_*) δ 10.13 (s, 2H), 8.59 (s, 2H), 8.51 (s, 1H), 8.39 (d, J = 2.0 Hz, 2H), 8.36 (d, J = 1.9 Hz, 2H), 8.15 (t, J = 1.9 Hz, 2H), 7.63 (s, 1H), 7.61 (s, 1H), 7.54 (d, J = 2.0 Hz, 1H), 7.51 (d, J = 2.0 Hz, 1H), 4.31 (t, J = 7.3 Hz, 4H), 1.97 – 1.86 (m, 4H), 1.44 (s, 18H), 1.36 (dt, J = 14.7, 7.4 Hz, 4H), 0.95 (t, J = 7.4 Hz, 6H).

Synthesis of **10-(3,5-dibromophenyl)-10H-phenoxazine**:

The synthetic protocol of **10-(3,5-dibromophenyl)-10H-phenoxazine** is the same as that of **9-(3,5-dibromophenyl)-9H-carbazole** except using 10H-phenoxazine as reagent. ^1^H NMR (500 MHz, DMSO-*d_6_*) δ 8.05 (t, J = 1.8 Hz, 1H), 7.80 (d, J = 1.8 Hz, 2H), 6.77 – 6.73 (m, 2H), 6.73 – 6.71 (m, 2H), 6.71 – 6.68 (m, 2H), 5.95 – 5.89 (m, 2H).

Synthesis of **10-(3,5-di(1H-imidazol-1-yl)phenyl)-10H-phenoxazine**:

The synthetic protocol of **10-(3,5-di(1H-imidazol-1-yl)phenyl)-10H-phenoxazine** is the same as that of **9-(3,5-di(1H-imidazol-1-yl)phenyl)-9H-carbazole** except using **10-(3,5-dibromophenyl)-10H-phenoxazine** as reagent. ^1^H NMR (400 MHz, DMSO-*d_6_*) δ 8.56 (s, 2H), 8.22 (t, J = 2.0 Hz, 1H), 8.04 (s, 2H), 7.87 (d, J = 2.0 Hz, 2H), 7.16 (s, 2H), 6.80 – 6.76 (m, 2H), 6.74 – 6.71 (m, 2H), 6.71 – 6.67 (m, 2H), 6.14 – 6.02 (m, 2H).

Synthesis of **1,1'-(5-(10H-phenoxazin-10-yl)-1,3-phenylene)bis(3-butyl-1H-imidazol-3-ium) dibromide (Pxz-pbib):**

The synthetic protocol is the same as that of **1,1'-(5-(9H-carbazol-9-yl)-1,3-phenylene)bis(3-butyl-1H-imidazol-3-ium) dibromide** except using **10-(3,5-di(1H-imidazol-1-yl)phenyl)-10H-phenoxazine** as reagent. ^1^H NMR (400 MHz, DMSO-*d_6_*) δ 10.08 (s, 2H), 8.58 (s, 1H), 8.53 (s, 2H), 8.25 (d, J = 2.0 Hz, 2H), 8.13 (t, J = 1.9 Hz, 2H), 6.85 (dd, J = 7.8, 1.7 Hz, 2H), 6.79 (td, J = 7.6, 1.7 Hz, 2H), 6.74 (td, J = 7.6, 1.8 Hz, 2H), 6.12 (dd, J = 7.8, 1.6 Hz, 2H), 4.28 (t, J = 7.2 Hz, 4H), 1.96 – 1.83 (m, 4H), 1.41 – 1.27 (m, 4H), 0.94 (t, J = 7.3 Hz, 6H).

**General Synthesis of Ir(III) complexes:**

Under the protection of nitrogen, [Ir(COD)Cl]_2_ (300 mg, 0.447 mmol, 1.0 eq) and tridentate chelates (**Cz-pbib**, **tBuCz-pbib**, **Pxz-pbib**) (0.894 mmol, 2.0 eq) were added to a mixture of triethylamine (1 ml) and acetonitrile (15 ml) in a dried Schlenk tube. The suspension was heated at 90 ℃ for 12 h. After cooling to ambient temperature, the solvent was removed by rotary evaporation to yield yellow solid intermediate. Then the yellow solid was dissolved in propionic acid (15 ml). The bidentate ligand, 2-(2,4-difluorophenyl)pyridine (dfppy) (170 mg, 0.894 mmol, 2.0 eq) and triethylamine (1 ml) were added to the solution and was heated at 150 ℃ for 24 h under nitrogen atmosphere. The solution was then cooled to ambient temperature before the solvent was removed by rotary evaporation. The crude product was purified by column chromatography. The intermediate product was then recrystallized in DCM/diethyl ether.

Under the protection of nitrogen, the intermediate product and AgCN (2 eq) were dissolved in 20 ml DMF and was heated at 100 ℃ for 1 h. Then the solvent was removed under reduced pressure to afford the crude product, which was then purified by column chromatography to yield the final product. Then the final product was recrystallized in DCM/diethyl ether.

Synthesis of **Cz-dfppy-Br:**

Under the protection of nitrogen, [Ir(COD)Cl]_2_ (300 mg, 0.447 mmol, 1.0 eq) and **Cz-pbib** (581 mg, 0.894 mmol, 2.0 eq) were added to a mixture of triethylamine (1 ml) and acetonitrile (15 ml) in a dried Schlenk tube. The suspension was heated at 90 ℃ for 12 h. After cooling to ambient temperature, the solvent was removed by rotary evaporation to yield yellow solid intermediate. Then the yellow solid was dissolved in propionic acid (15 ml). The bidentate ligand, **2-(2,4-difluorophenyl)pyridine (dfppy)** (171 mg, 0.894 mmol, 2.0 eq) and triethylamine (1 ml) were added to the solution and was heated at 150 ℃ for 24 h under nitrogen atmosphere. The solution was then cooled to ambient temperature before the solvent was removed by rotary evaporation. The crude product was purified by column chromatography (eluent = DCM/PE 5:1 v/v), then recrystallized in DCM/diethyl ether to afford the expected product **Cz-dfppy-Br**(195 mg, 46%). ^1^H NMR (400 MHz, Chloroform-d) δ 10.56 (dt, J = 5.1, 1.4 Hz, 1H), 8.42 (d, J = 8.5 Hz, 1H), 8.19 (d, J = 7.8 Hz, 2H), 7.90 (dd, J = 8.4, 6.8 Hz, 1H), 7.54 (t, J = 8.3 Hz, 2H), 7.47 (s, 2H), 7.41 – 7.35 (m, 3H), 7.33 (s, 2H), 7.31 (s, 1H), 6.76 (d, J = 2.1 Hz, 2H), 6.31 (ddd, J = 13.1, 8.9, 2.5 Hz, 1H), 5.66 (dd, J = 9.4, 2.5 Hz, 1H), 3.37 – 3.28 (m, 2H), 3.28 – 3.18 (m, 2H), 1.50 – 1.35 (m, 2H), 1.22 – 1.09 (m, 2H), 0.96 – 0.85 (m, 2H), 0.85 – 0.76 (m, 2H), 0.73 (t, J = 6.8 Hz, 6H).

Synthesis of **Cz-dfppy-CN:**

Under the protection of nitrogen, **Cz-dfppy-Br** (195 mg, 0.206 mmol) and AgCN (55 mg, 0.412 mmol, 2 eq) were dissolved in 20 ml DMF and was heated at 100 ℃ for 1 h. Then the solvent was removed under reduced pressure to afford the crude product, which was then purified by column chromatography (eluent = DCM:EA 20:1) and recrystallized in DCM/diethyl ether to afford the expected product **Cz-dfppy-CN** (132 mg, 72%). ^1^H NMR (400 MHz, Chloroform-d) δ 10.36 – 10.27 (m, 1H), 8.43 (d, J = 9.3 Hz, 1H), 8.20 (d, J = 7.7 Hz, 2H), 7.93 (t, J = 7.8 Hz, 1H), 7.53 – 7.49 (m, 2H), 7.49 – 7.44 (m, 2H), 7.43 (d, J = 2.1 Hz, 2H), 7.34 (d, J = 1.6 Hz, 1H), 7.33 – 7.30 (m, 4H), 6.80 (d, J = 2.1 Hz, 2H), 6.33 (ddd, J = 13.2, 8.9, 2.5 Hz, 1H), 5.65 (dd, J = 8.5, 2.4 Hz, 1H), 3.32 (ddd, J = 13.4, 9.8, 6.1 Hz, 2H), 3.27 – 3.17 (m, 2H), 1.45 (qt, J = 10.0, 5.7 Hz, 2H), 1.19 (ddd, J = 17.2, 11.4, 7.1 Hz, 2H), 1.00 – 0.79 (m, 4H), 0.74 (t, J = 7.0 Hz, 6H).

Synthesis of **tBuCz-dfppy-Br:**

Under the protection of nitrogen, [Ir(COD)Cl]_2_ (300 mg, 0.447 mmol, 1.0 eq) and **tBuCz-pbib** (681 mg, 0.894 mmol, 2.0 eq) were added to a mixture of triethylamine (1 ml) and acetonitrile (15 ml) in a dried Schlenk tube. The suspension was heated at 90 ℃ for 12 h. After cooling to ambient temperature, the solvent was removed by rotary evaporation to yield yellow solid intermediate. Then the yellow solid was dissolved in propionic acid (15 ml). The bidentate ligand **dfppy** (171 mg, 0.894 mmol, 2.0 eq) and triethylamine (1 ml) were added to the solution and was heated at 150 ℃ for 24 h under nitrogen atmosphere. The solution was then cooled to ambient temperature before the solvent was removed by rotary evaporation. The crude product was purified by column chromatography (eluent = DCM/PE 5:1 v/v), then recrystallized in DCM/diethyl ether to afford the expected product **tBuCz-dfppy-Br** (362 mg, 76%). ^1^H NMR (400 MHz, Chloroform-d) δ 10.57 (d, J = 5.6 Hz, 1H), 8.41 (d, J = 8.3 Hz, 1H), 8.19 (s, 2H), 7.89 (t, J = 7.9 Hz, 1H), 7.50 (s, 3H), 7.39 (d, J = 1.9 Hz, 1H), 7.36 (d, J = 6.3 Hz, 1H), 7.33 (s, 1H), 6.75 (d, J = 2.2 Hz, 2H), 6.35 – 6.26 (m, 1H), 5.63 (d, J = 8.9 Hz, 1H), 3.39 – 3.27 (m, 2H), 3.27 – 3.16 (m, 2H), 1.50 (s, 18H), 1.46 – 1.36 (m, 2H), 1.22 – 1.07 (m, 2H), 0.94 – 0.84 (m, 2H), 0.84 – 0.75 (m, 2H), 0.73 (t, J = 6.7 Hz, 3H).

Synthesis of **tBuCz-dfppy-CN:**

Under the protection of nitrogen, **tBuCz-dfppy-Br** (362 mg, 0.341 mmol, 1 eq) and AgCN (91 mg, 0.682 mmol, 2.0 eq) were dissolved in 20 ml DMF and was heated at 100 ℃ for 1 h. Then the solvent was removed under reduced pressure to afford the crude product, which was then purified by column chromatography (eluent = DCM:EA 20:1) and recrystallized in DCM/diethyl ether to afford the expected product **tBuCz-dfppy-CN** (278 mg, 81%). ^1^H NMR (400 MHz, Chloroform-d) δ 10.32 (d, J = 5.6 Hz, 1H), 8.43 (d, J = 8.6 Hz, 1H), 8.19 (d, J = 1.9 Hz, 2H), 7.93 (t, J = 7.8 Hz, 1H), 7.53 (d, J = 1.9 Hz, 1H), 7.51 (d, J = 2.0 Hz, 1H), 7.45 (s, 1H), 7.43 (s, 1H), 7.41 (d, J = 2.1 Hz, 2H), 7.33 (d, J = 6.4 Hz, 1H), 7.31 (s, 2H), 6.79 (d, J = 2.1 Hz, 2H), 6.32 (ddd, J = 11.6, 8.9, 2.5 Hz, 1H), 5.62 (dd, J = 8.5, 2.5 Hz, 1H), 3.38 – 3.27 (m, 2H), 3.27 – 3.15 (m, 2H), 1.50 (s, 18H), 1.47 – 1.38 (m, 2H), 1.18 (dt, J = 15.7, 7.3 Hz, 2H), 0.96 – 0.88 (m, 2H), 0.87 – 0.81 (m, 2H), 0.74 (t, J = 7.0 Hz, 6H).

Synthesis of **Pxz-dfppy-Br:**

Under the protection of nitrogen, [Ir(COD)Cl]_2_ (300 mg, 0.447 mmol, 1.0 eq) and **Pxz-pbib** (595 mg, 0.894 mmol, 2.0 eq) were added to a mixture of triethylamine (1 ml) and acetonitrile (15 ml) in a dried Schlenk tube. The suspension was heated at 90 ℃ for 12 h. After cooling to ambient temperature, the solvent was removed by rotary evaporation to yield yellow solid intermediate. Then the yellow solid was dissolved in propionic acid (15 ml). The bidentate ligand **dfppy** (171 mg, 0.894 mmol, 2.0 eq) and triethylamine (1 ml) were added to the solution and was heated at 150 ℃ for 24 h under nitrogen atmosphere. The solution was then cooled to ambient temperature before the solvent was removed by rotary evaporation. The crude product was purified by column chromatography (eluent = DCM/PE 5:1 v/v), then recrystallized in DCM/diethyl ether to afford the expected product **Pxz-dfppy-Br** (220 mg, 51%). ^1^H NMR (400 MHz, Chloroform-d) δ 10.54 (d, J = 5.4 Hz, 1H), 8.40 (d, J = 8.6 Hz, 1H), 7.93 – 7.85 (m, 1H), 7.40 (d, J = 2.1 Hz, 2H), 7.36 (ddd, J = 7.2, 5.6, 1.3 Hz, 1H), 7.13 (s, 2H), 6.75 (d, J = 2.0 Hz, 2H), 6.72 (s, 2H), 6.65 (s, 3H), 6.35 – 6.12 (m, 3H), 5.54 (dd, J = 9.4, 2.5 Hz, 1H), 3.39 – 3.26 (m, 2H), 3.26 – 3.14 (m, 2H), 1.47 – 1.35 (m, 2H), 1.21 – 1.11 (m, 2H), 0.91 – 0.84 (m, 2H), 0.84 – 0.76 (m, 2H), 0.72 (t, J = 6.9 Hz, 6H).

Synthesis of **Pxz-dfppy-CN:**

Under the protection of nitrogen, **Pxz-dfppy-Br** (220 mg, 0.228 mmol, 1 eq) and AgCN (61 mg, 0. mmol, 2.0 eq) were dissolved in 20 ml DMF and was heated at 100 ℃ for 1 h. Then the solvent was removed under reduced pressure to afford the crude product, which was then purified by column chromatography (eluent = DCM:EA 20:1) and recrystallized in DCM/diethyl ether to afford the expected product **Pxz-dfppy-CN** (143 mg, 68%). ^1^H NMR (400 MHz, Chloroform-d) δ 10.26 (d, J = 5.5 Hz, 1H), 8.42 (d, J = 8.1 Hz, 1H), 7.93 (t, J = 7.9 Hz, 1H), 7.43 (d, J = 2.1 Hz, 2H), 7.36 – 7.30 (m, 1H), 7.14 (s, 2H), 6.80 (d, J = 2.1 Hz, 2H), 6.72 (d, J = 4.3 Hz, 2H), 6.68 (d, J = 6.4 Hz, 4H), 6.35 – 6.26 (m, 1H), 6.19 (s, 2H), 5.51 (dd, J = 8.7, 2.5 Hz, 1H), 3.36 – 3.27 (m, 2H), 3.26 – 3.16 (m, 2H), 1.49 – 1.39 (m, 2H), 1.22 – 1.13 (m, 2H), 0.96 – 0.87 (m, 2H), 0.86 – 0.78 (m, 2H), 0.74 (t, J = 7.0 Hz, 6H).

# NMR Spectra


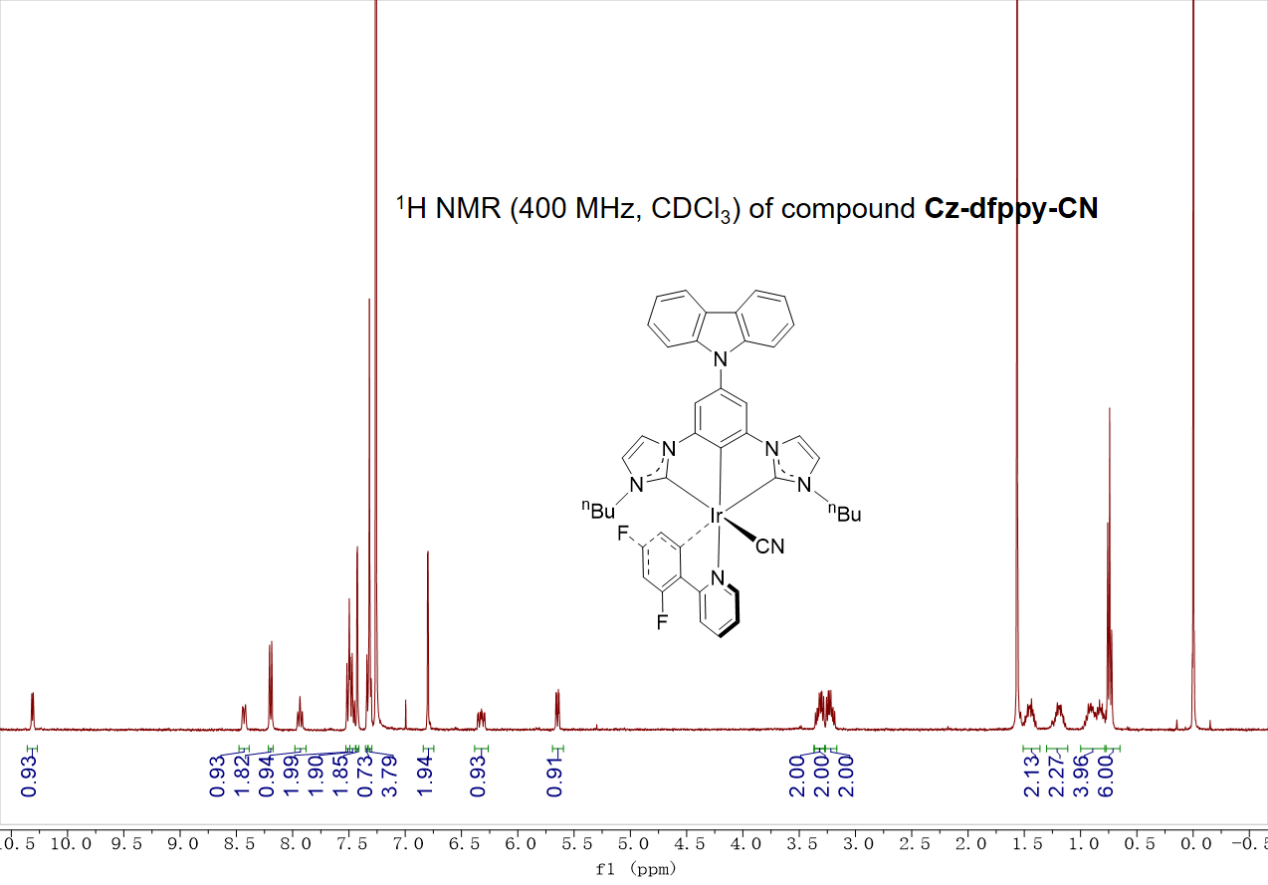


**Figure S25.** ^1^H NMR (400 MHz. CDCl3) of compound **Cz-dfppy-CN.**


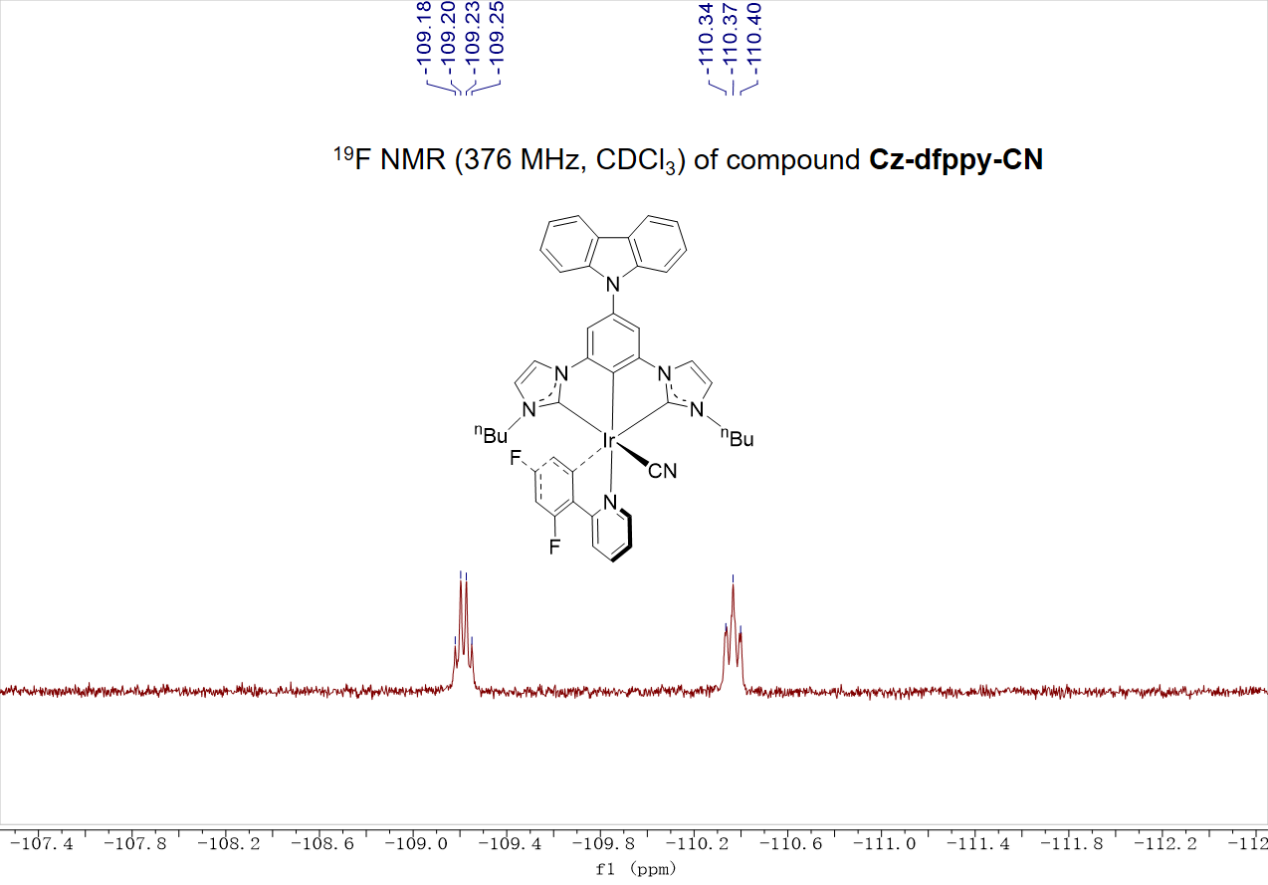


**Figure S26.** ^19^F NMR (376 MHz, CDCl3) of compound **Cz-dfppy-CN.**


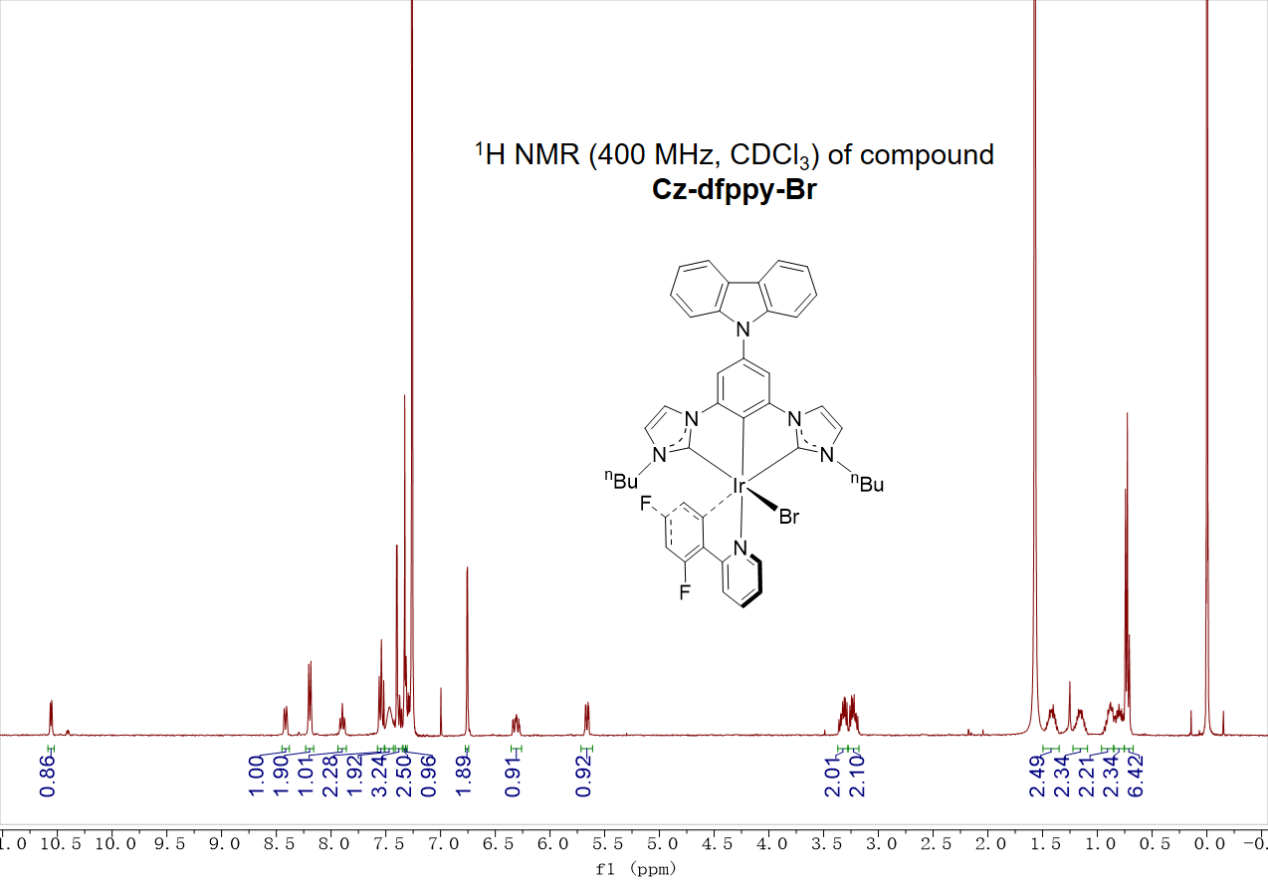


**Figure S27.** ^1^H NMR (400 MHz, CDCl3) of compound **Cz-dfppy-Br.**


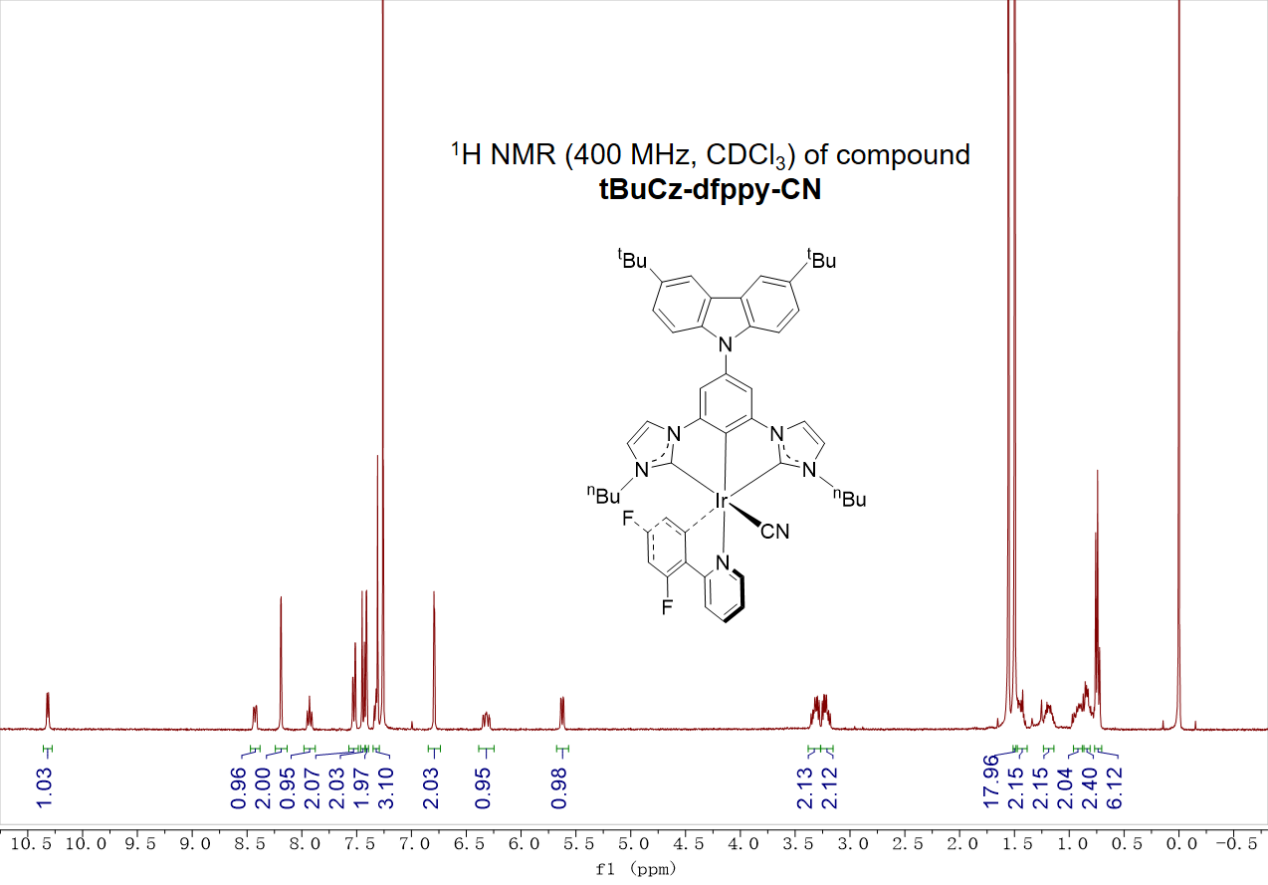


**Figure S28.** ^1^H NMR (400 MHz. CDCl3) of compound **tBuCz-dfppy-CN.**


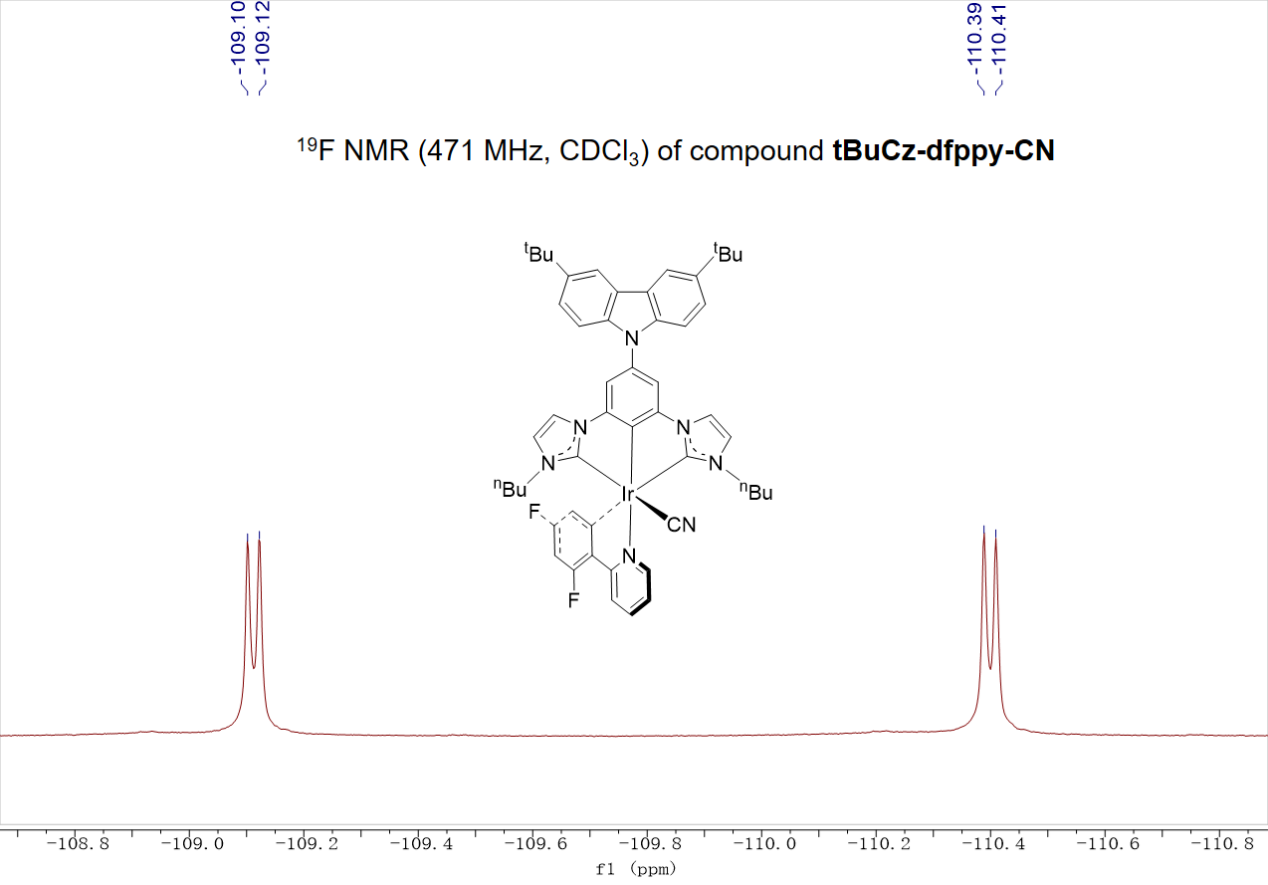


**Figure S29.** ^19^F NMR (471 MHz, CDCl3) of compound **tBuCz-dfppy-CN.**


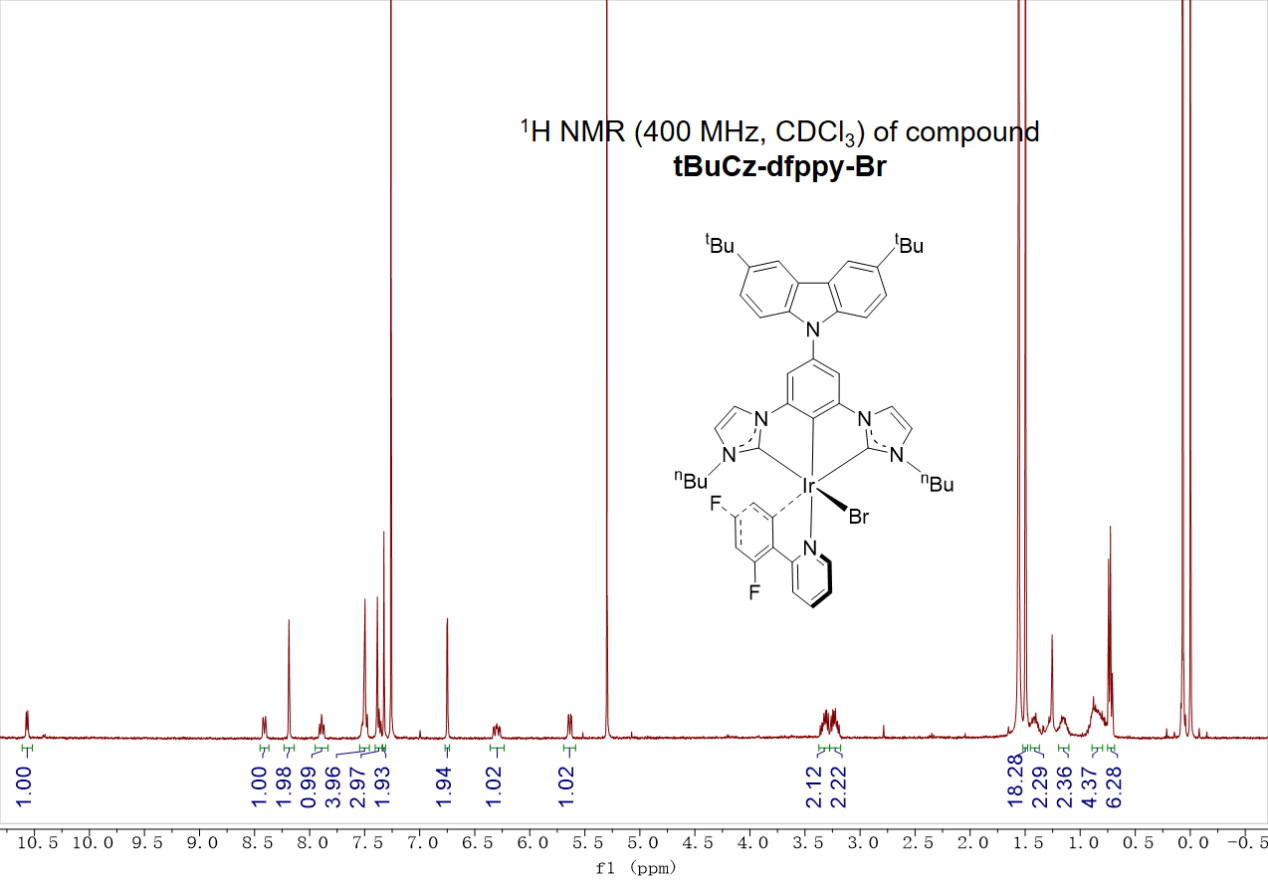


**Figure S30.** ^1^H NMR (400 MHz, CDCl3) of compound **tBuCz-dfppy-CN.**


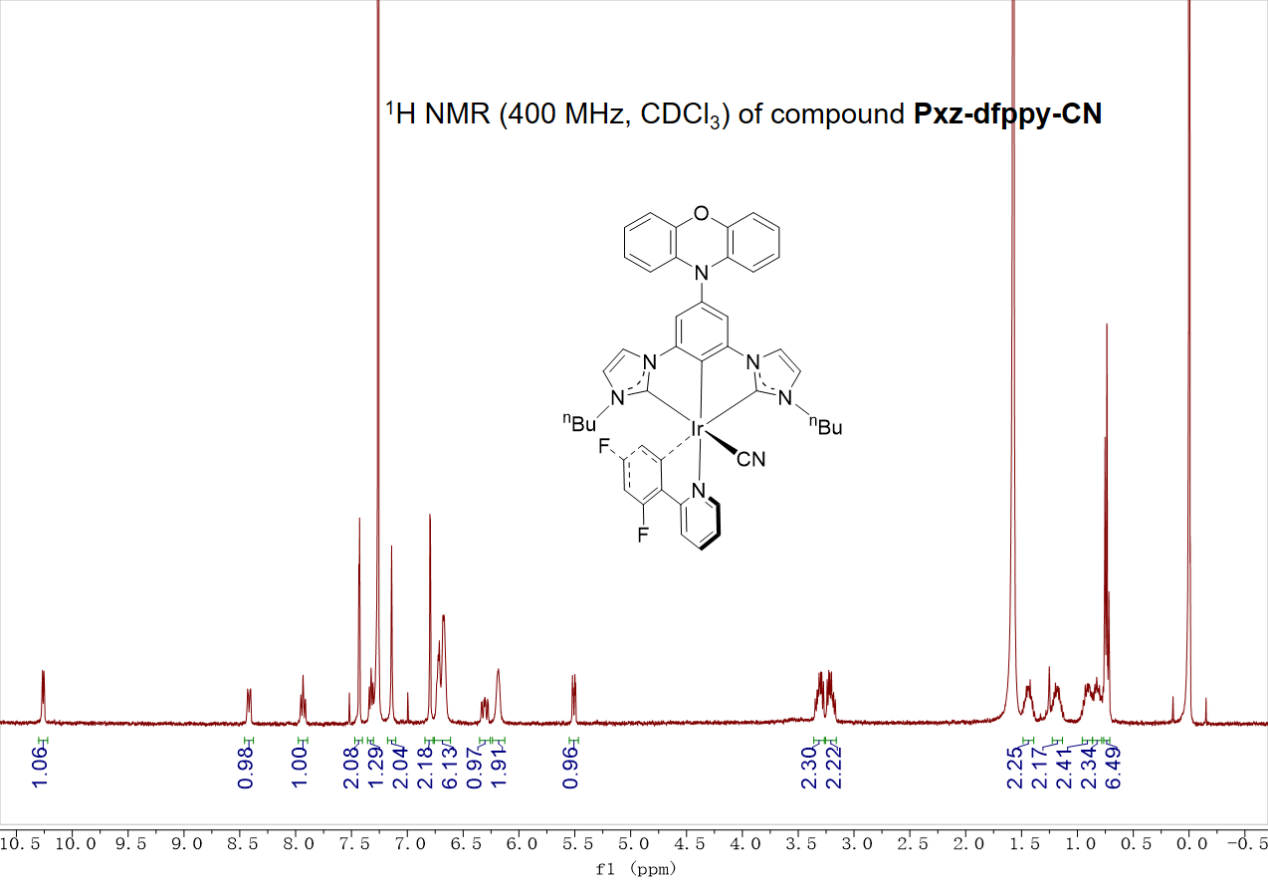


**Figure S31.** ^1^H NMR (400 MHz, CDCl3) of compound **Pxz-dfppy-CN.**


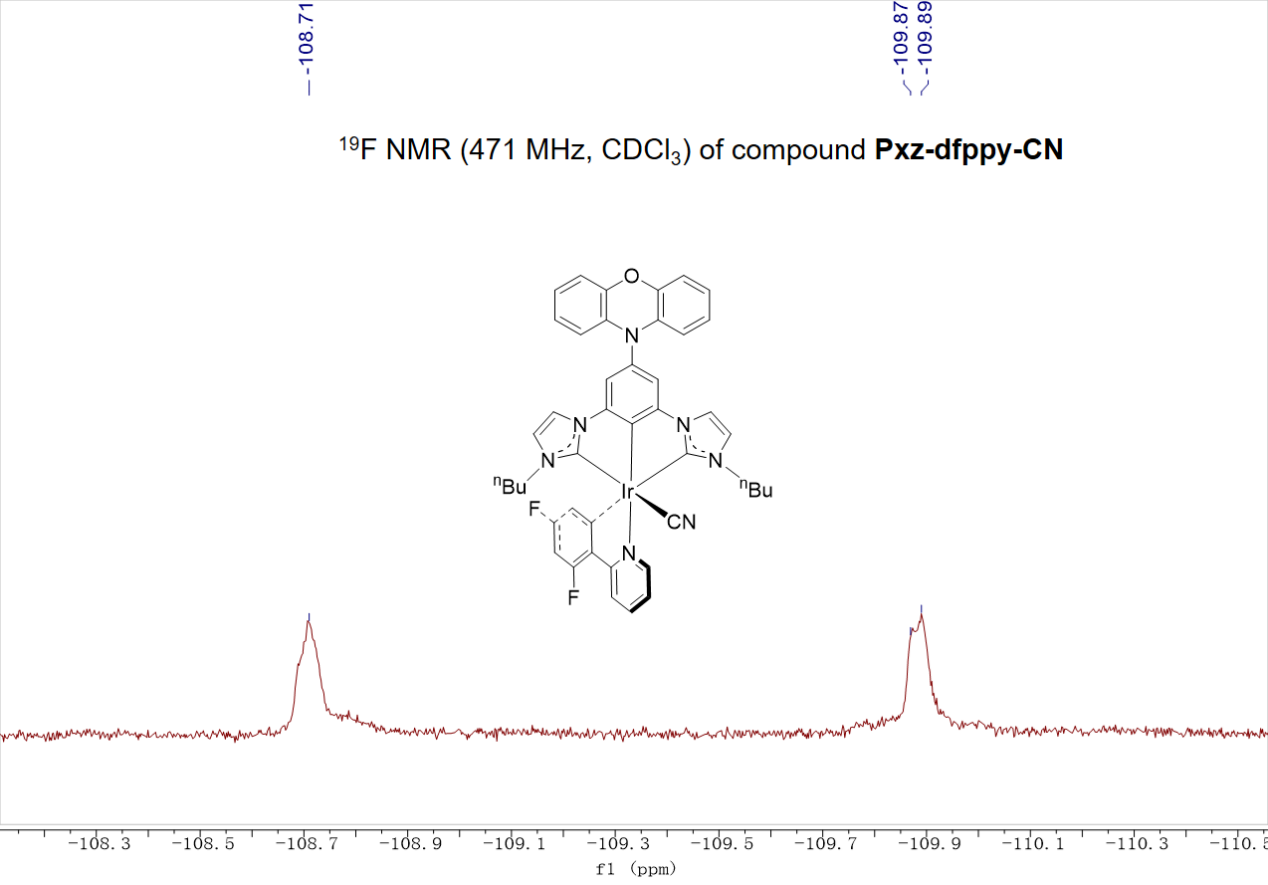


**Figure S32.** ^19^F NMR (471 MHz, CDCl3) of compound **Pxz-dfppy-CN.**


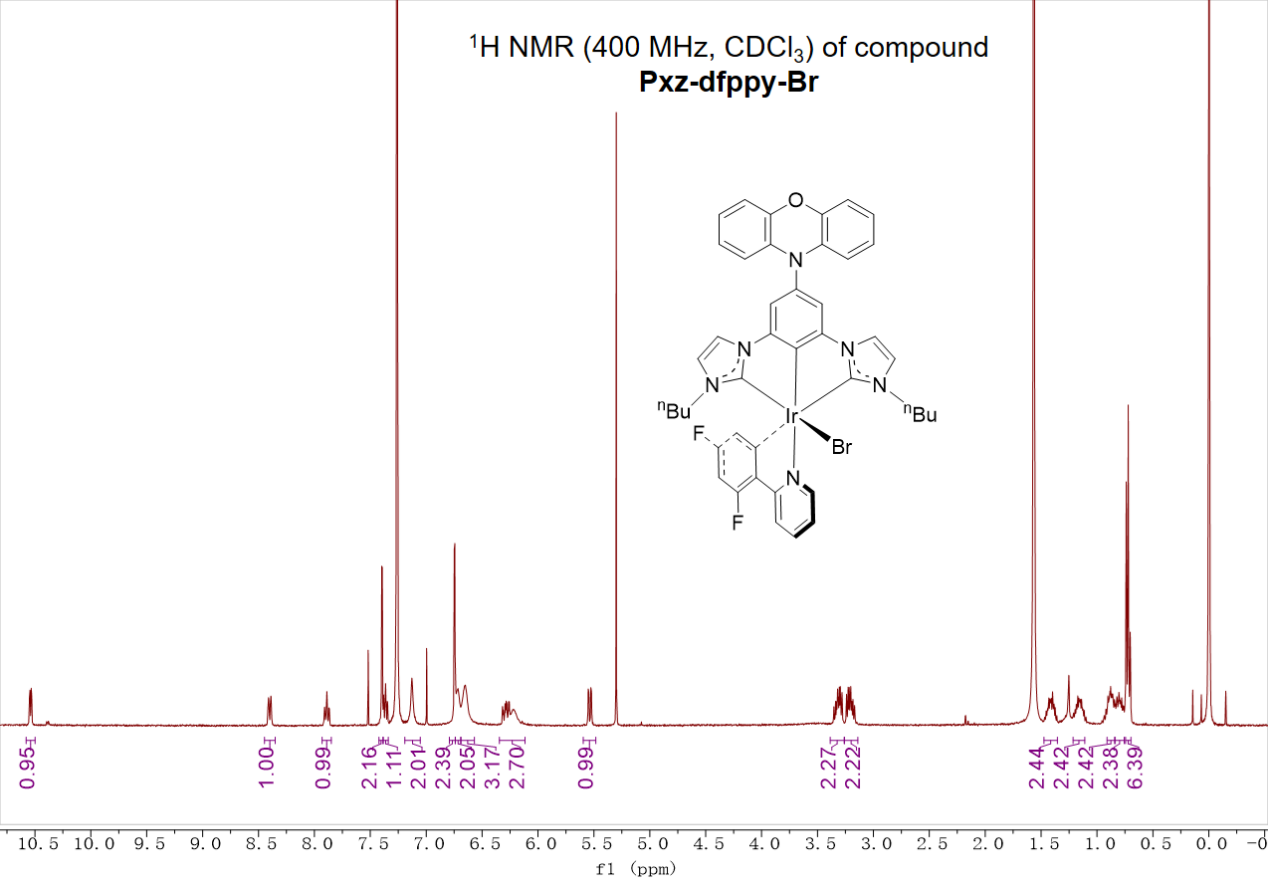


**Figure S33.** ^1^H NMR (400 MHz, CDCl3) of compound **Pxz-dfppy-Br.**


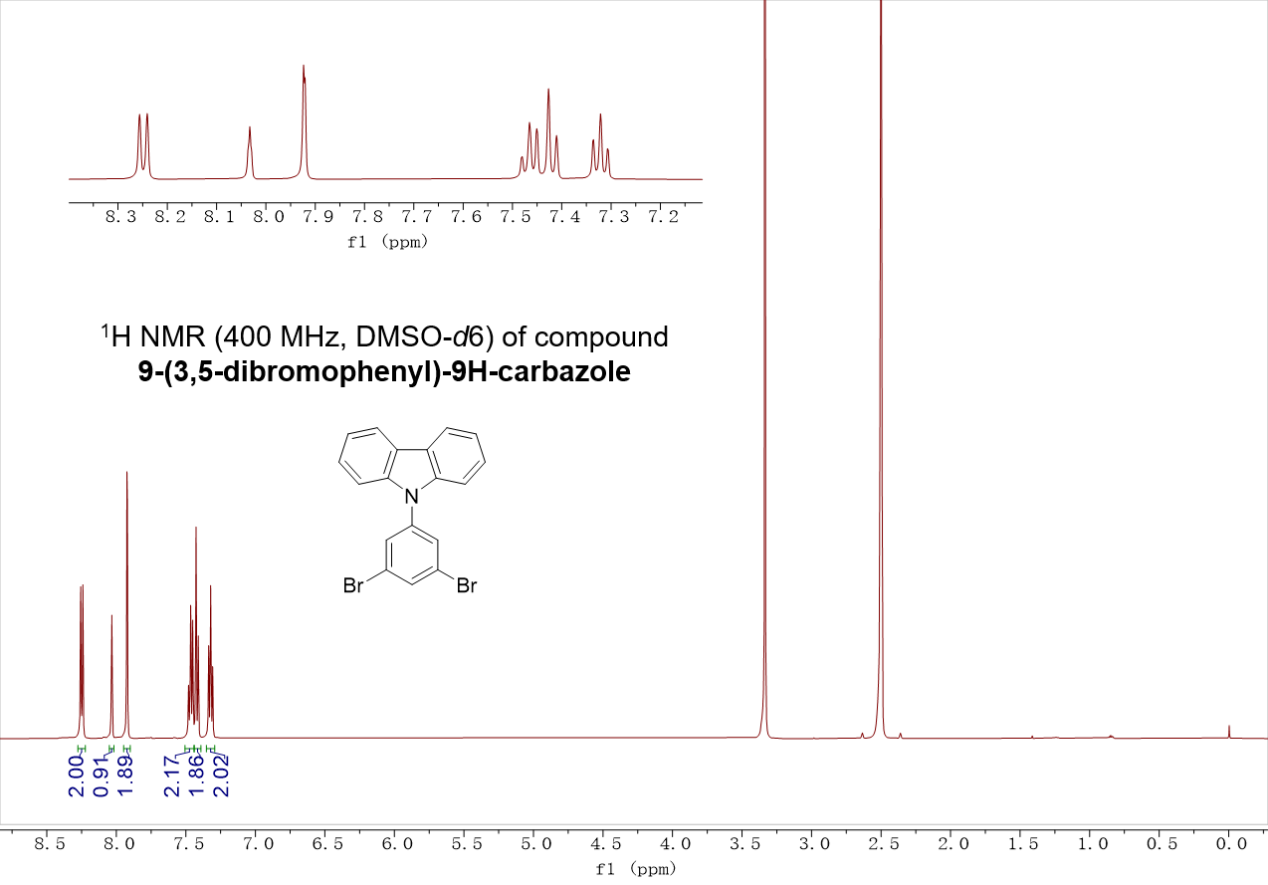


**Figure S34.** ^1^H NMR (400 MHz, DMSO−*d_6_*) of compound 9−(3,5−dibromophenyl)−9H−carbazole.


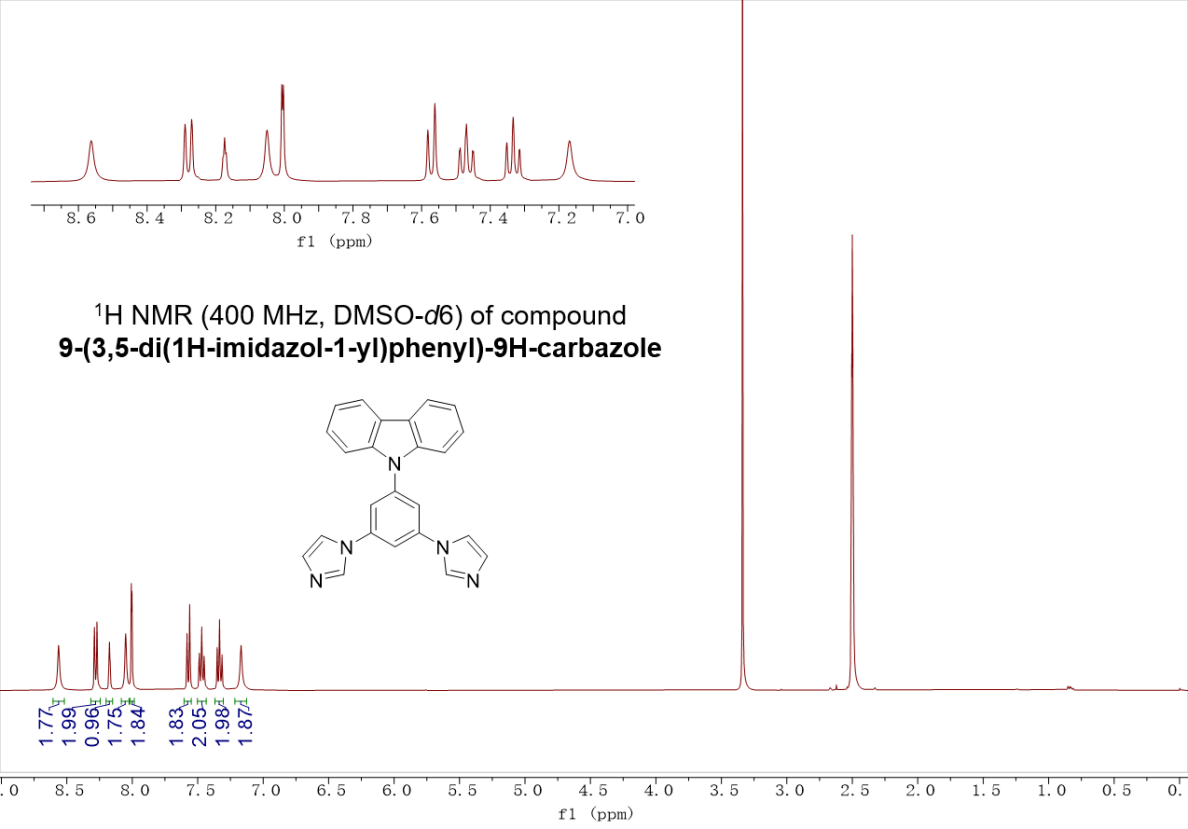


**Figure S35.** ^1^H NMR (400 MHz, DMSO−*d_6_*) of compound 9−(3,5−di(1H−imidazol−1−yl)phenyl)−9H−carbazole.


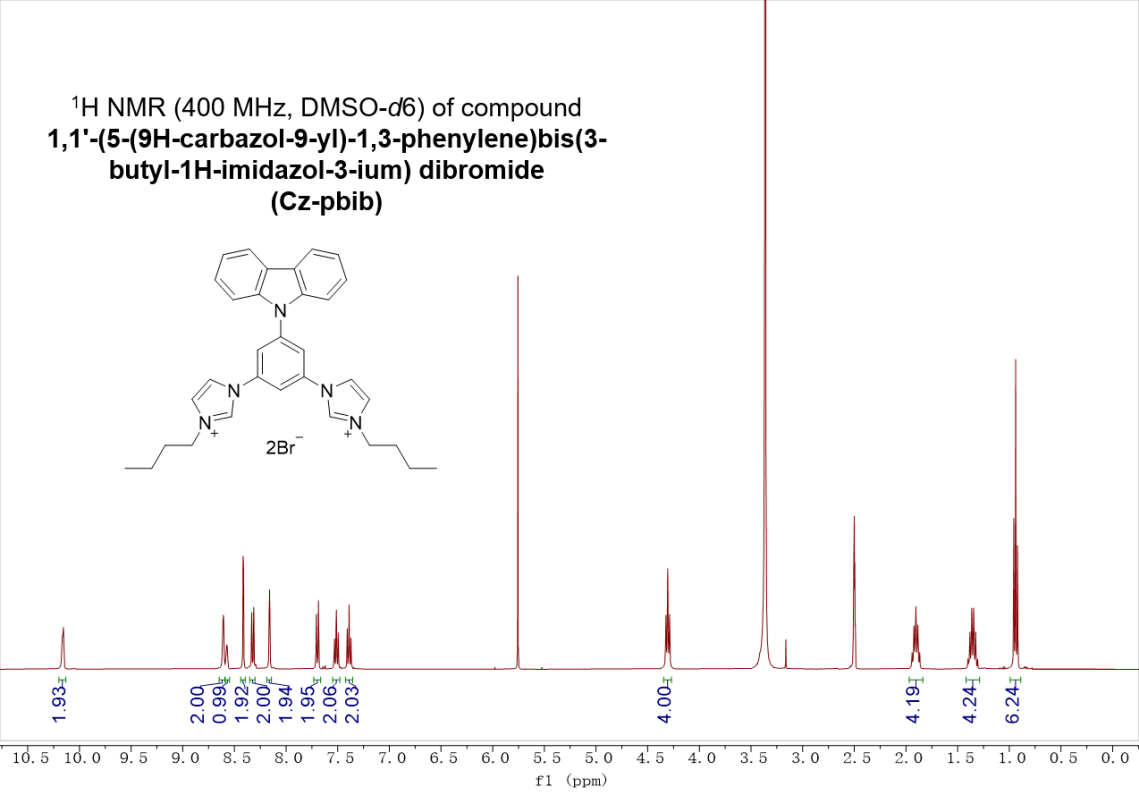


**Figure S36.** ^1^H NMR (400 MHz, DMSO−*d_6_*) of compound 1,1'−(5−(9H−carbazol−9−yl)−1,3−phenylene)bis(3−butyl−1H−imidazol−3−ium) dibromide (**Cz−pbib**).


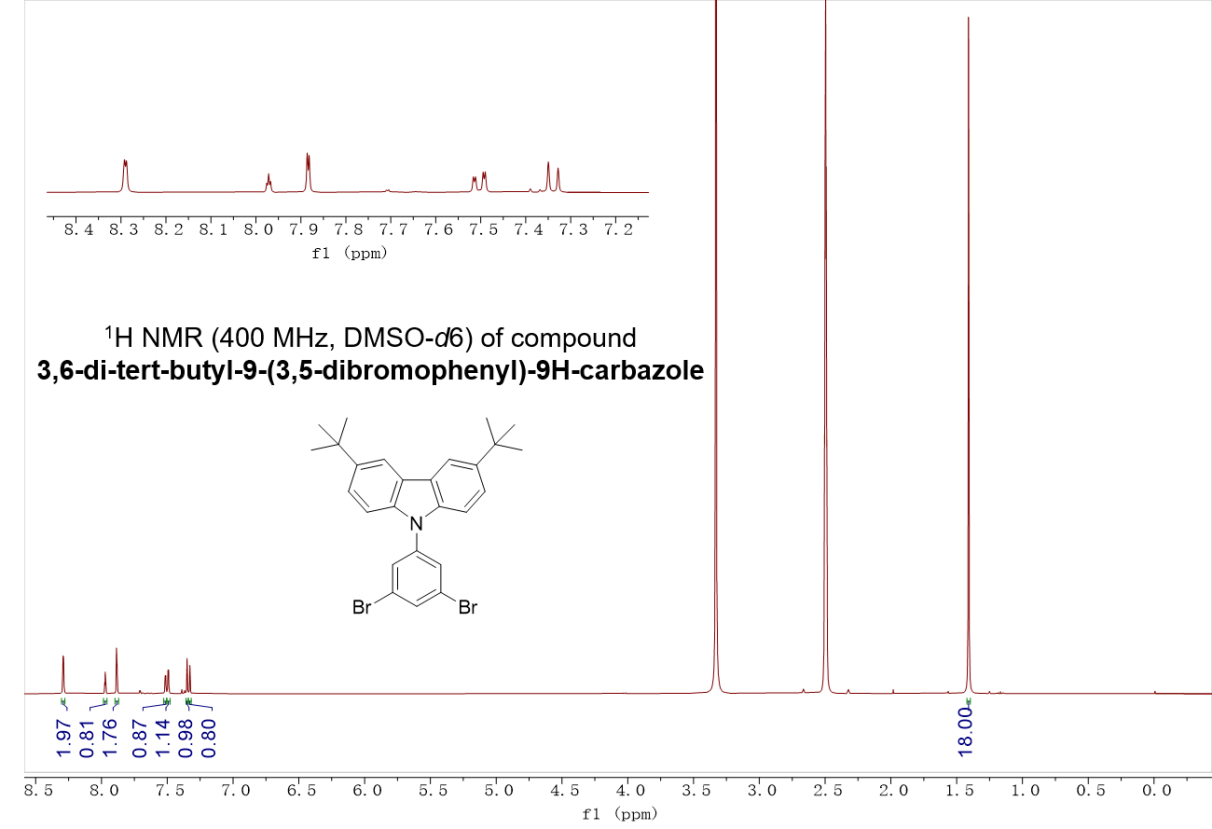


**Figure S37.** ^1^H NMR (400 MHz, DMSO−*d_6_*) of compound 3,6−di−tert−butyl−9−(3,5−dibromophenyl)−9H−carbazole.


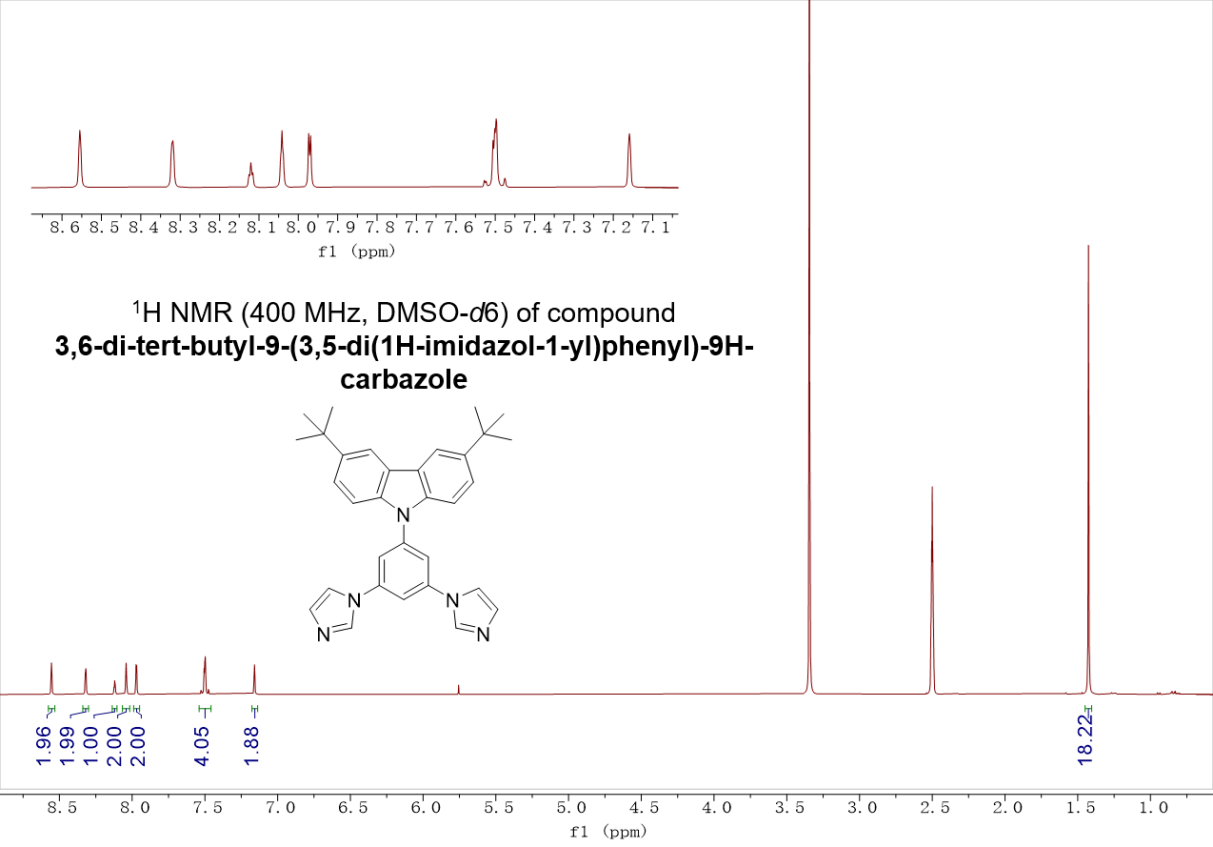


**Figure S38.** ^1^H NMR (400 MHz, DMSO−*d_6_*) of compound 3,6−di−tert−butyl−9−(3,5−di(1H−imidazol−1−yl)phenyl)−9H−carbazole.


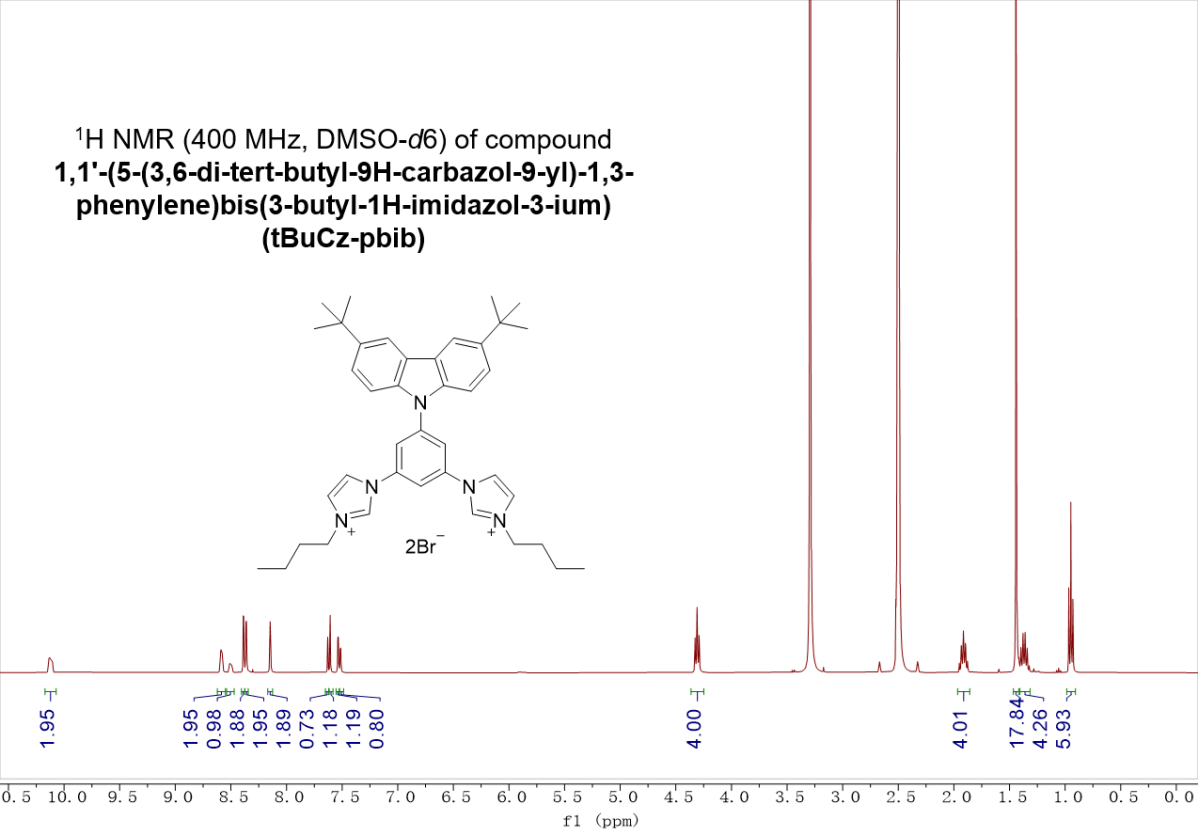


**Figure S39.** ^1^H NMR (400 MHz, DMSO−*d_6_*) of compound 1,1'−(5−(3,6−di−tert−butyl−9H−carbazol−9−yl)−1,3−phenylene)bis(3−butyl−1H−imidazol−3−ium) (**tBuCz−pbib**).


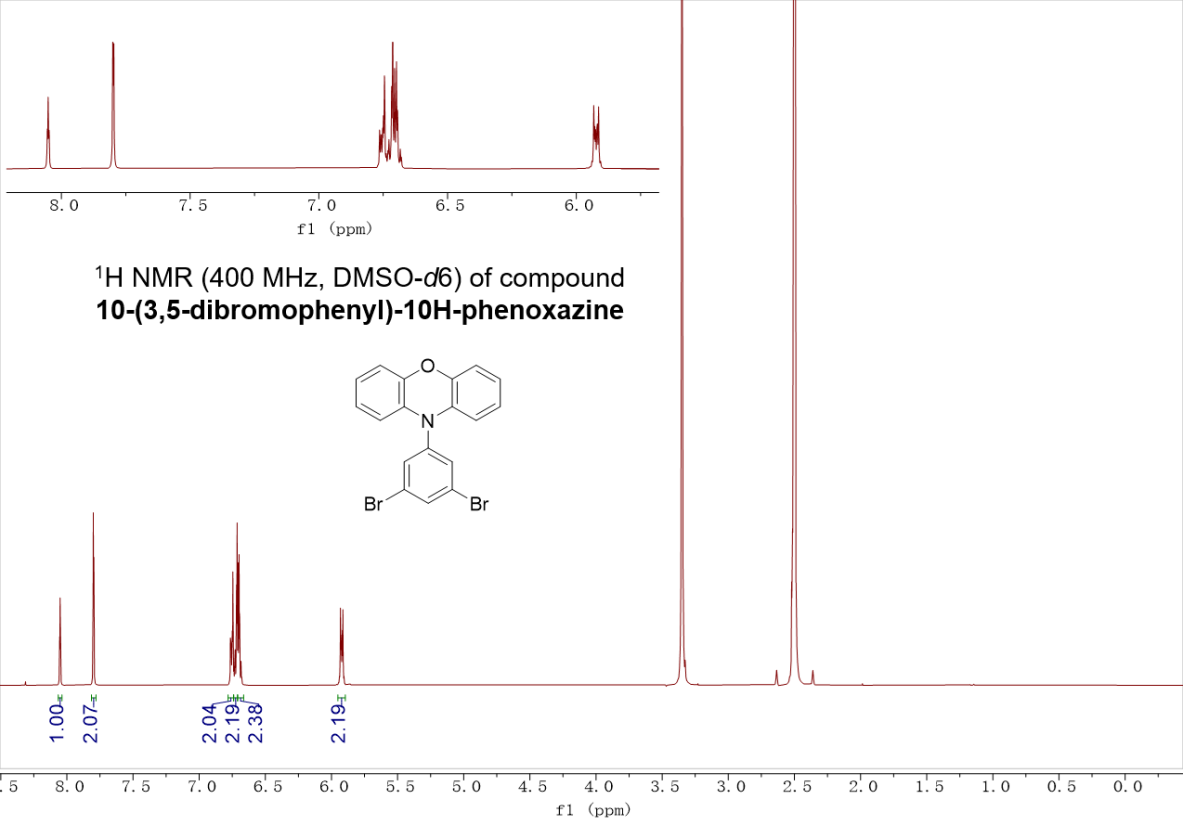


**Figure S40.** ^1^H NMR (400 MHz, DMSO−*d_6_*) of compound 10−(3,5−dibromophenyl)−10H−phenoxazine.


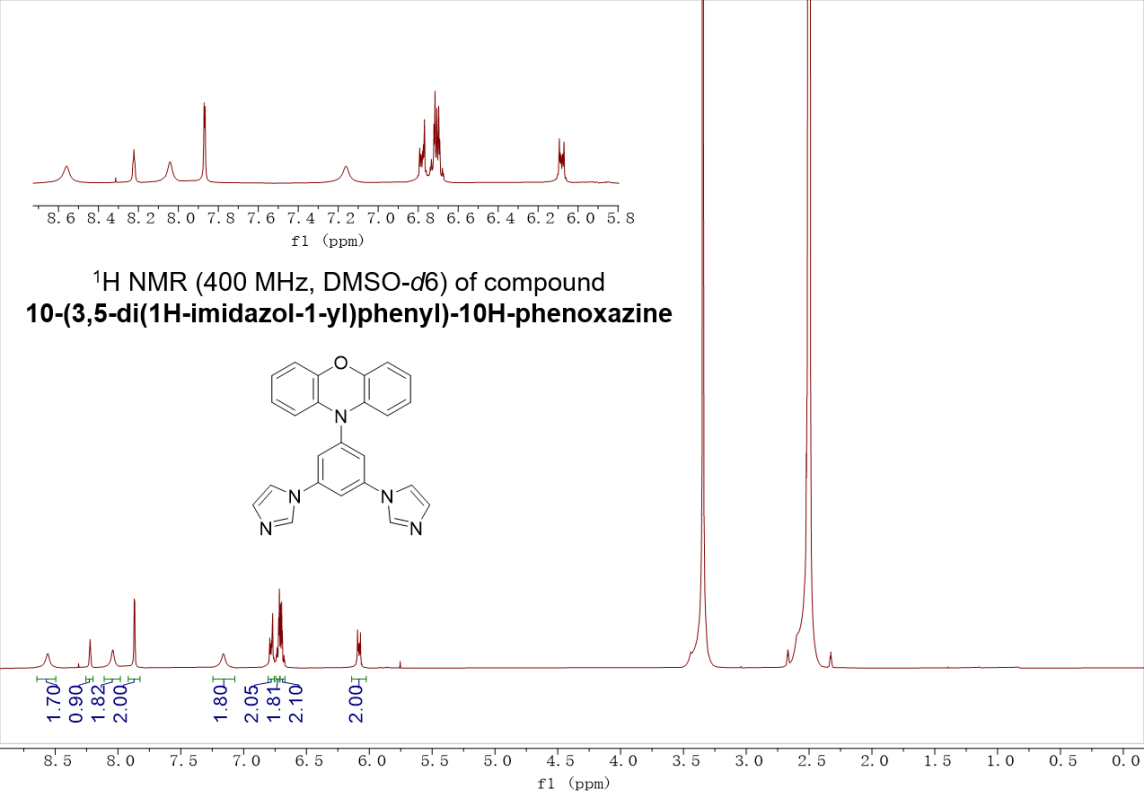


**Figure S41.** ^1^H NMR (400 MHz, DMSO−*d_6_*) of compound 10−(3,5−di(1H−imidazol−1−yl)phenyl)−10H−phenoxazine.


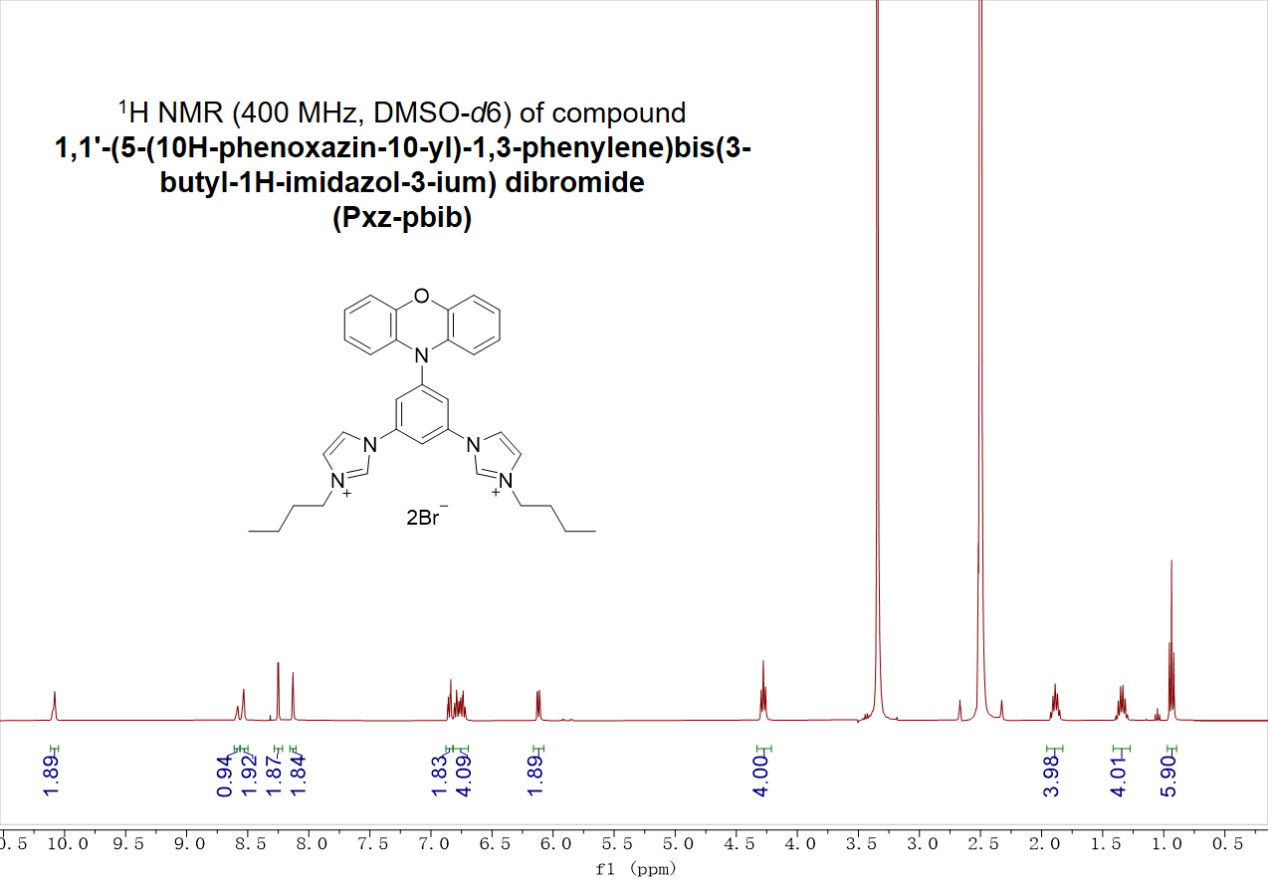


**Figure S42.** ^1^H NMR (400 MHz, DMSO−*d_6_*) of compound 1,1'−(5−(10H−phenoxazin−10−yl)−1,3−phenylene)bis(3−butyl−1H−imidazol−3−ium) dibromide (**Pxz−pbib**).

# ES-MS of Ir(III) complexes


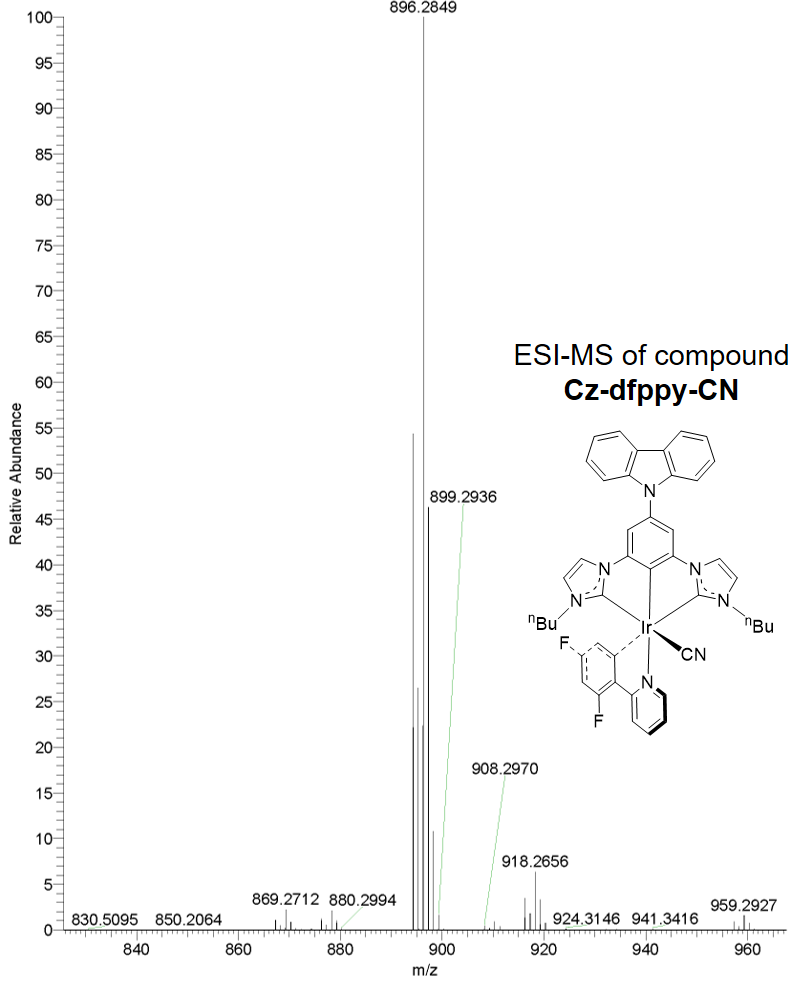


**Figure S43.** ESI−MS of compound **Cz-dfppy-CN.**


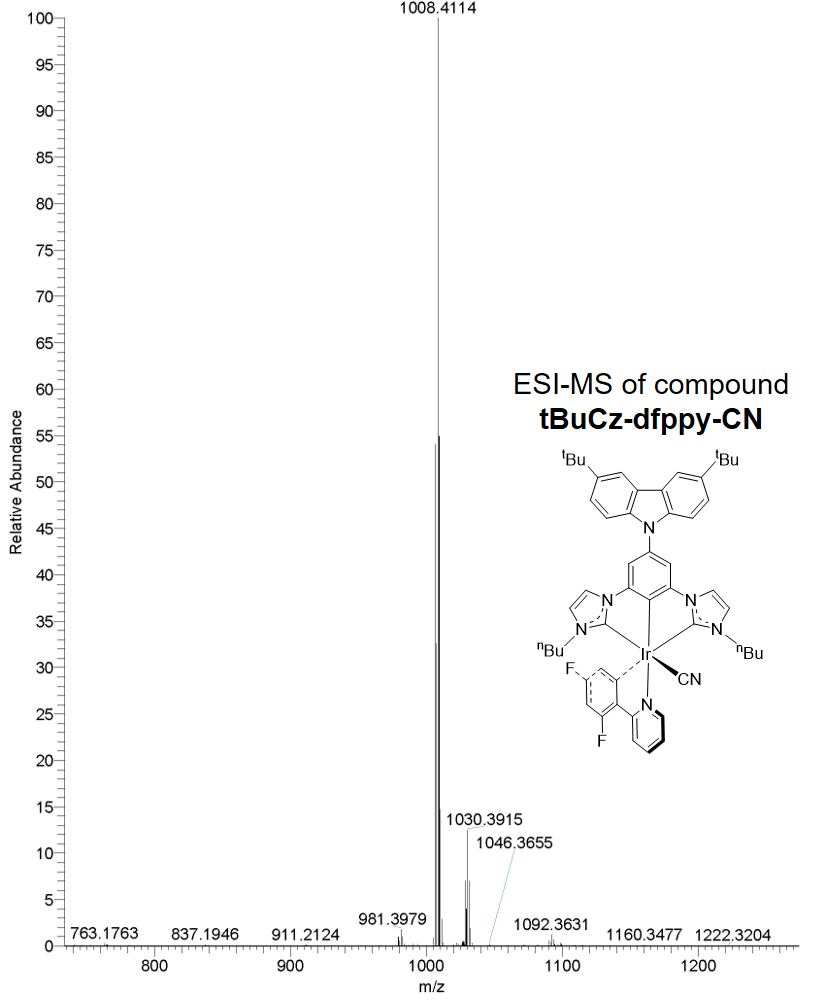


**Figure S44.** ESI−MS of compound **tBuCz-dfppy-CN.**


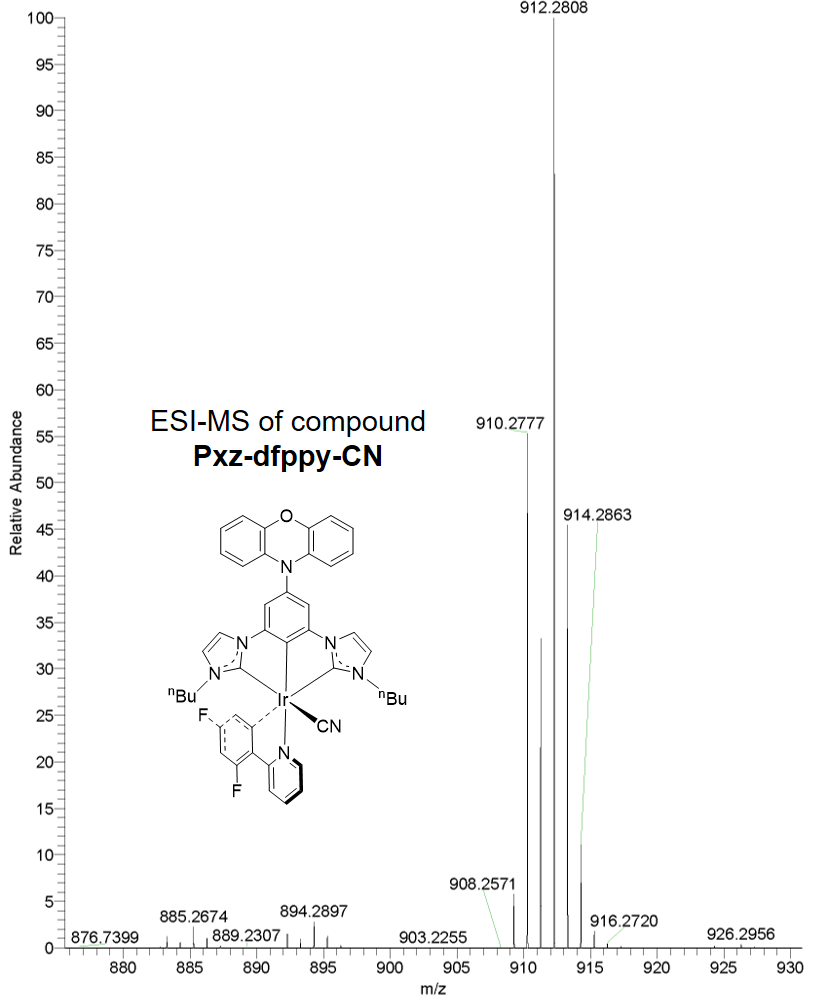


**Figure S45.** ESI−MS of compound **Pxz-dfppy-CN.**

# Reference

[1] M. J. Frisch, G. W. Trucks, H. B. Schlegel, G. E. Scuseria, M. A. Robb, J. R. Cheeseman, G. Scalmani, V. Barone, G. A. Petersson, H. Nakatsuji, X. Li, M. Caricato, A. V. Marenich, J. Bloino, B. G. Janesko, R. Gomperts, B. Mennucci, H. P. Hratchian, J. V. Ortiz, A. F. Izmaylov, J. L. Sonnenberg, Williams, F. Ding, F. Lipparini, F. Egidi, J. Goings, B. Peng, A. Petrone, T. Henderson, D. Ranasinghe, V. G. Zakrzewski, J. Gao, N. Rega, G. Zheng, W. Liang, M. Hada, M. Ehara, K. Toyota, R. Fukuda, J. Hasegawa, M. Ishida, T. Nakajima, Y. Honda, O. Kitao, H. Nakai, T. Vreven, K. Throssell, J. A. Montgomery Jr., J. E. Peralta, F. Ogliaro, M. J. Bearpark, J. J. Heyd, E. N. Brothers, K. N. Kudin, V. N. Staroverov, T. A. Keith, R. Kobayashi, J. Normand, K. Raghavachari, A. P. Rendell, J. C. Burant, S. S. Iyengar, J. Tomasi, M. Cossi, J. M. Millam, M. Klene, C. Adamo, R. Cammi, J. W. Ochterski, R. L. Martin, K. Morokuma, O. Farkas, J. B. Foresman, D. J. Fox, Gaussian, Inc., Wallingford, CT 2016.

[2] P. Hohenberg, W. Kohn, *Physical Review* **1964**, 136, B864.

[3] W. Kohn, L. J. Sham, *Physical Review* **1965**, 140, A1133.

[4] C. Lee, W. Yang, R. G. Parr, *Physical Review B* **1988**, 37, 785.

[5] A. D. Becke, *The Journal of Chemical Physics* **1993**, 98, 5648.

[6] P. J. Stephens, F. J. Devlin, C. F. Chabalowski, M. J. Frisch, *The Journal of Physical Chemistry* **1994**, 98, 11623.

[7] V. Barone, M. Cossi, *The Journal of Physical Chemistry A* **1998**, 102, 1995.

[8] M. Cossi, N. Rega, G. Scalmani, V. Barone, *Journal of Computational Chemistry* **2003**, 24, 669.

[9] E. Runge, E. K. U. Gross, *Physical Review Letters* **1984**, 52, 997.

[10] M. E. Casida, *Journal of Molecular Structure: THEOCHEM* **2009**, 914, 3.

[11] P. J. Hay, W. R. Wadt, *The Journal of Chemical Physics* **1985**, 82, 299.

[12] P. J. Hay, W. R. Wadt, *The Journal of Chemical Physics* **1985**, 82, 270.

[13] W. R. Wadt, P. J. Hay, *The Journal of Chemical Physics* **1985**, 82, 284.

[14] A. W. Ehlers, M. Böhme, S. Dapprich, A. Gobbi, A. Höllwarth, V. Jonas, K. F. Köhler, R. Stegmann, A. Veldkamp, G. Frenking, *Chemical Physics Letters* **1993**, 208, 111.

[15] P. C. Hariharan, J. A. Pople, *Theoretica chimica acta* **1973**, 28, 213.

[16] M. M. Francl, W. J. Pietro, W. J. Hehre, J. S. Binkley, M. S. Gordon, D. J. DeFrees, J. A. Pople, *The Journal of Chemical Physics* **1982**, 77, 3654.

[17] W. J. Hehre, R. Ditchfield, J. A. Pople, *The Journal of Chemical Physics* **2003**, 56, 2257.

[18] T. Lu, F. Chen, *Journal of Computational Chemistry* **2012**, 33, 580.

[19] J. Zhang, T. Lu, *Physical Chemistry Chemical Physics* **2021**, 23, 20323.
